# PPARG contributes to urothelial integrity in the murine ureter by activating the expression of *Shh* and superficial cell-specific genes

**DOI:** 10.1242/dev.204324

**Published:** 2025-04-17

**Authors:** Carsten Rudat, Philipp Straube, Jan Hegermann, Mark-Oliver Trowe, Hauke Thiesler, Herbert Hildebrandt, Lisa Witt, Andreas Kispert

**Affiliations:** ^1^ Institute of Molecular Biology, Hannover Medical School, 30625 Hannover, Germany; ^2^ Institute of Functional and Applied Anatomy, Research Core Unit Electron Microscopy, Hannover Medical School, 30625 Hannover, Germany; ^3^ Institute of Clinical Biochemistry, Hannover Medical School, 30625 Hannover, Germany; ^4^ Center for Systems Neuroscience Hannover, Hannover, Germany

**Keywords:** Ureter, Urothelium, *Pparg*, Shh, Quiescence, Superficial cell, Differentiation

## Abstract

The urothelium is a stratified epithelium with an important barrier function in the urinary drainage system. The differentiation and maintenance of the three major urothelial cell types (basal, intermediate and superficial cells) is incompletely understood. Here, we show that mice with a conditional deletion of the transcription factor gene peroxisome proliferator activated receptor gamma (*Pparg*) in the ureteric epithelium have a dilated ureter at postnatal stages with a urothelium consisting of a layer of undifferentiated luminal cells and a layer of proliferating basal cells. Molecular analysis of fetal stages revealed that the expression of a large number of genes is not activated in superficial cells and that of a few genes, including *Shh*, is not activated in intermediate and basal cells. Pharmacological activation of SHH signaling in explant cultures of perinatal *Pparg*-deficient ureters reduced ureteral width and urothelial cell number to normal levels, increased the number of intermediate cells and slightly reduced basal cell proliferation. Our data suggest that PPARG independently activates the expression of structural genes in superficial cells and of *Shh* in basal and intermediate cells, and that both functions contribute to urothelial integrity.

## Teaser

**Summary:** PPARG is required for *Shh* expression in the basal and intermediate cell layer of the mouse ureteric epithelium and for expression of many genes in the superficial layer, ensuring the formation of a three-layered, quiescent urothelium.

## INTRODUCTION

In the main organs of the mammalian urinary drainage system, the ureters and the bladder, a highly specialized stratified epithelium, the urothelium, lines the luminal surfaces. The urothelium acts primarily as a physical barrier, preventing the uncontrolled exchange of substances between the urine and interstitial fluids. Central to this function is the luminal layer of umbrella or superficial (S) cells, which are interconnected by tight junctions. S cells are large and highly expandable post-mitotic cells with two or more nuclei. They are characterized by the synthesis of specialized glycoproteins, uroplakins (UPKs), which assemble into semi-crystalline plaques on the apical surface. S cells are underlain by small intermediate (I) cells that form layers with one to several tiers. A layer of cuboidal basal (B) cells connects the urothelium to the basement membrane and the fibromuscular wall (Arrighi, 2015; Castillo-Martin et al., 2010; Dalghi et al., 2020). In addition to shape and location, urothelial cells can be molecularly distinguished by the combinatorial expression of cytokeratin 5 (KRT5), an isoform of the transcription factor p63 (ΔNP63) and UPKs. B cells are KRT5^+^ΔNP63^+^UPK1B^−^, I cells are KRT5^−^ΔNP63^+^UPK1B^+^ and S cells are KRT5^−^ΔNP63^−^UPK1B^+^, with levels of UPK1B being significantly lower in I cells than in S cells (Bohnenpoll et al., 2017a; Gandhi et al., 2013).

The urothelial cytoarchitecture results from highly coordinated proliferation, patterning and differentiation processes acting on epithelial progenitors (the cloaca in the case of the bladder, or the stalk region of the ureteric bud in the case of the ureter), starting in the mouse around embryonic day (E) 10.5. After proliferative expansion of these mono-layered epithelial primordia, stratification and I cell differentiation occur. Two days later, luminal cells express UPKs, and after another 2 days, B cell differentiation begins. I cells continue to proliferate between these two layers. Under homeostatic conditions, the urothelium is largely quiescent with a limited replenishment of B cells and S cells by I cells (Bohnenpoll et al., 2017a; Gandhi et al., 2013). In response to injury or infection, S cells are shed and replaced by I cells (Gandhi et al., 2013). When S cells and I cells are depleted, a small subset of B cells, characterized by expression of KRT14 and possibly KRT15, may act as progenitors for all urothelial cells (Papafotiou et al., 2016; Tai et al., 2013).

The regulation of urothelial differentiation is poorly understood but several studies have implicated the ligand-dependent transcription factor peroxisome proliferator activated receptor gamma (PPARG) in this program. PPARG was originally characterized as a member of a small subset of the nuclear receptor superfamily with important roles in lipid metabolism. Peroxisome proliferator activated receptors (PPARs) form heterodimers with retinoid X receptors (RXRs) and bind to specific peroxisome proliferator response elements (PPREs) in the promoters of target genes. Upon binding of specific ligands, these heterodimeric complexes bind to coactivator complexes to activate gene transcription (for reviews, see Blanquart et al., 2003; Desvergne and Wahli, 1999; Varga et al., 2011).

Expression of PPARG protein was first described in the presumptive urothelium of the mouse urogenital sinus and in the mature urothelium of mice, rabbits and humans (Guan et al., 1997; Jain et al., 1998; Kawakami et al., 2002). Activation of PPARG by high-affinity agonists in cultures of normal urothelial cells suppresses the growth of these cells (Nakashiro et al., 2001), and induces gene expression changes associated with S-cell differentiation (Varley et al., 2006, 2004a). Conditional deletion of *Pparg* in progenitors of the urothelium of the mouse ureter results in increased proliferation of B cells and a lack of UPK and KRT20 expression in S cells at postnatal stages (Weiss et al., 2013). Conditional deletion of *Pparg* in bladder urothelial progenitors leads to squamous differentiation of B cells, loss of I cells and a failure of S-cell differentiation in adult mice. The latter phenotype is associated with mitochondrial defects and alterations in lipid metabolism (Liu et al., 2019).

While these studies support a role for PPARG in differentiation of S cells and maintenance of B cells in the developing ureter and bladder, it is unclear whether these functions are linked and what the targets of PPARG transcriptional activity are in the different urothelial cell types. Understanding the precise molecular function of this transcription factor is important because *PPARG* variants are frequently associated with urothelial cancer in humans (Rochel et al., 2019; Tate et al., 2021). Here, we used conditional gene targeting in combination with transcriptional profiling, data mining and pharmacological rescue experiments to address *Pparg* function in the mouse ureter. Our results show that PPARG activates the expression of *Shh* in B cells and I cells, as well as structural S cell genes, and that both gene programs cooperate to maintain urothelial integrity.

## RESULTS

### PPARG is differentially expressed in urothelial cells of the ureter

**Fig. 1.**
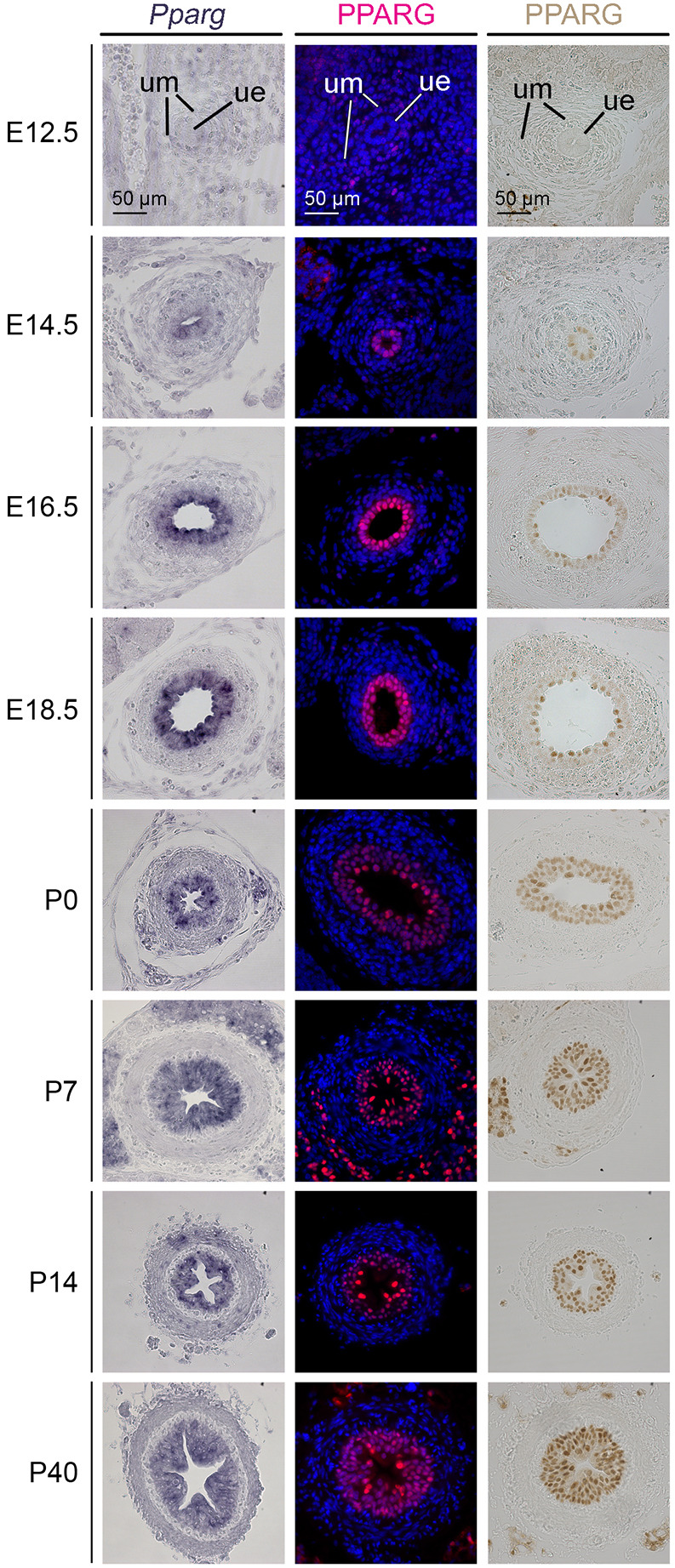
**PPARG is differentially expressed in urothelial cells.** Expression analysis on sections of the proximal ureter region derived from wild-type mice at prenatal and postnatal stages for *Pparg* mRNA by RNA *in situ* hybridization (first column) and for PPARG protein by immunofluorescence (second column) and immunohistochemistry (third column). Cell nuclei are counterstained with DAPI (second column). *n*≥3 for each stage and assay. ue, ureteric epithelium; um, ureteric mesenchyme.

To obtain a detailed profile of *Pparg* expression during the development of the murine ureter, we performed RNA *in situ* hybridization analysis on transverse ureter sections from wild-type mice at different prenatal and postnatal stages. *Pparg* expression started at E14.5 in the ureteric epithelium (UE), which was still mono-layered at this stage. At E16.5, when the UE was two-layered, and from E18.5 to postnatal day (P) 40, when the urothelium was three-layered, expression of *Pparg* increased and was found in all epithelial cells (Fig. 1, first column). Immunofluorescence analysis detected nuclear PPARG protein in approximately half of the cells of the UE at E14.5. At later stages, PPARG protein was found in all urothelial cells. Expression appeared to be enhanced in the luminal cell layer (Fig. 1, second column). Since differences in expression levels can be masked by saturation of the amplification procedure in the immunofluorescence assay, we additionally used immunohistochemical detection. This method confirmed that PPARG expression in the luminal layer is increased compared to the basal and intermediate layer from E16.5 onwards (Fig. 1, third column).

### Loss of *Pparg* affects urothelial differentiation

To investigate the role of *Pparg* in the UE, we used a conditional gene inactivation approach with an allele of *Pparg* in which exons 2 and 3 are flanked by *loxP* sites, and a *Pax2-cre* line that mediates recombination in the nephric duct, the UE and the renal collecting duct system (Bohnenpoll et al., 2017a; He et al., 2003; Trowe et al., 2011).

Using the exon2/3 region and the 3′-UTR of a *Pparg* cDNA (reference transcript: NM011146.3) as probes in RNA *in situ* hybridization analysis, we detected expression of a *Pparg* mRNA lacking the exon2/3 region in the UE of E16.5 and E18.5 *Pax2-cre/+;Pparg^fl/fl^* (*Pparg-cKO*) embryos. At E18.5, expression of the mutant transcript was increased compared to the control, indicating a compensatory upregulation (Fig. S1A). Importantly, immunohistochemical analysis using a monoclonal antibody raised against the N-terminal PPARG region detected a protein at E16.5 and E18.5 in the control but not in the mutant UE (Fig. S1B). This is consistent with the prediction that cre-mediated deletion of exon2/3 from the floxed *Pparg* allele results in a truncated protein lacking the N-terminal region, including parts of the DNA-binding domain, and thus a non-functional protein (He et al., 2003).

**Fig. 2.**
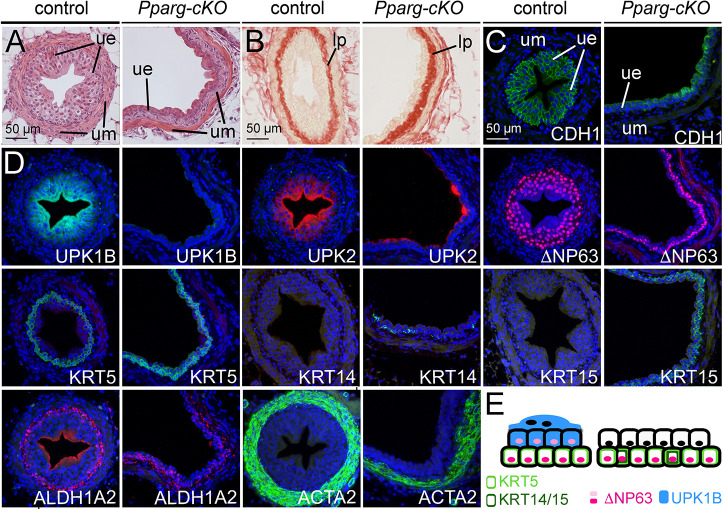
***Pparg-cKO* ureters are dilated and show urothelial stratification and differentiation defects at P40.** (A-D) Histological analysis by Hematoxylin and Eosin (A) and Sirius Red (B) staining, and immunofluorescence analysis of epithelial stratification (CDH1) (C) and cytodifferentiation of the urothelium (UPK1B, UPK2: S cells and I cells; ΔNP63: B cells and I cells; KRTs: B cells) and the ureteric mesenchyme (ALDH1A2: lamina propria; ACTA2: smooth muscle cells) (D) on transverse sections of the proximal region of P40 control and *Pparg-cKO* ureters. Nuclei are counterstained with DAPI. *n*≥3 for each assay, marker and genotype. lp, lamina propria; ue, ureteric epithelium; um, ureteric mesenchyme. (E) Scheme summarizing the stratification defects and the molecular alterations with respect to KRT5, KRT14, KRT15, ΔNP63 and UPK1B expression in the *Pparg-cKO* urothelium.

*Pparg-cKO* embryos and postnatal animals were found at the expected Mendelian ratio at all stages analyzed (Table S1). The morphological appearance of E18.5 isolated urogenital systems and of P40 kidneys with ureters of mutant mice was normal (Fig. S2). However, histological analysis of *Pparg-cKO* ureters at P40 revealed a greatly enlarged tubular lumen with an irregular outline (Fig. 2A,B, Fig. S3, Table S2). The urothelium had a highly eosinophilic appearance (Fig. 2A). Expression of CDH1, a marker for basolateral membranes in epithelial cells, was irregular with a partial apical localization. In addition, the mutant urothelium was two-layered and not three-layered as in the control at this stage (Fig. 2C). Cells in the luminal layer of the mutant urothelium were reduced in size compared to control luminal cells (Fig. 2C, Fig. S4, Table S3). Expression of UPK1B and UPK2, which weakly labeled I cells and strongly labeled S cells in control tissue, was absent (UPK1B) or restricted to a few luminal cells (UPK2) in the mutant. ΔNP63, a marker for B cells and I cells, was restricted to the basal layer in the mutant urothelium, which was also positive for the B cell marker KRT5. KRT14 and KRT15, which have been associated with a regenerative response in the bladder (Papafotiou et al., 2016; Tai et al., 2013), were ectopically expressed in B cells of *Pparg-cKO* ureters (Fig. 2D). KRT6 and KRT10, markers for squamous differentiation (Liu et al., 2019), were detected in neither control nor mutant urothelium (Fig. S5A). The expression of smooth muscle cell (SMC) markers (ACTA2, TAGLN, CKM) appeared irregular and slightly reduced in the mutant ureter, the expression of the lamina propria marker ALDH1A2 was normal (Fig. 2D, Fig. S5B). In conclusion, P40 *Pparg-cKO* ureters have a two-layered urothelium with undifferentiated luminal cells and differentiated B cells. The latter ectopically express KRT14 and KRT15 (Fig. 2E). The SMC layer also appears to be affected, possibly related to the dilation of the ureteral lumen.

### Urothelial defects in *Pparg-cKO* ureters begin around E16.5

**Fig. 3.**
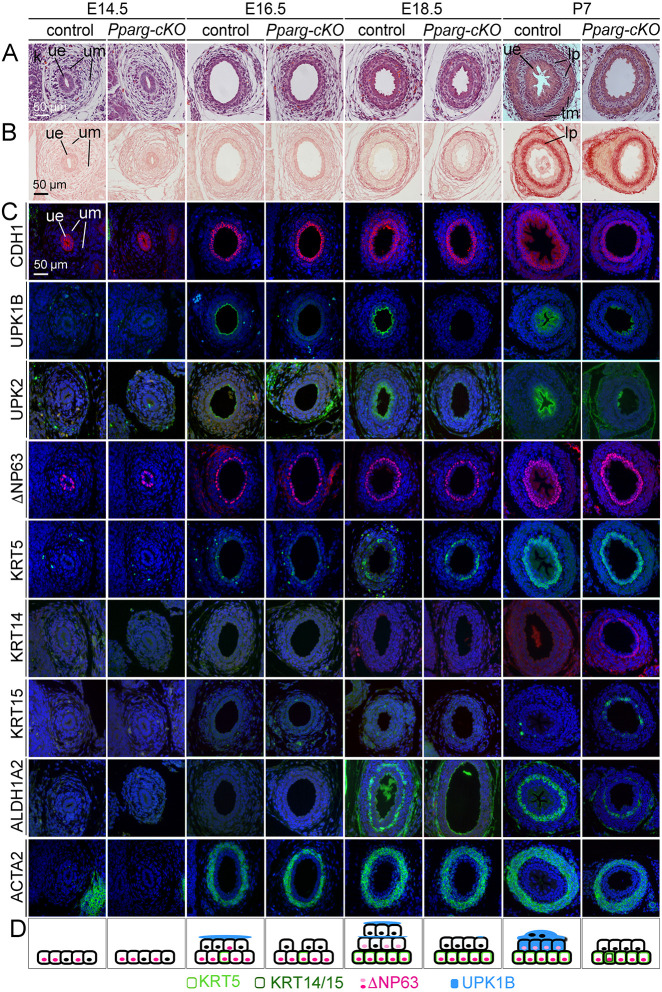
**Urothelial defects in *Pparg-cKO* ureters begin at E16.5 and progressively worsen thereafter.** Developmental time course of ureteral defects in *Pparg-cKO* embryos analyzed on sections of the proximal ureter region. (A,B) Histological analysis by Hematoxylin and Eosin (A) and Sirius Red (B) staining. (C) Immunofluorescence analysis of epithelial stratification (CDH1), cytodifferentiation of the urothelium (UPK1B, UPK2: S cells and I cells; ΔNP63: B cells and I cells; KRTs: B cells) and of the ureteric mesenchyme (ALDH1A2: lamina propria; ACTA2: smooth muscle cells). Cell nuclei are counterstained with DAPI. *n*≥3 for each stain and marker per genotype. k, kidney; lp, lamina propria; tm, tunica muscularis; ue, ureteric epithelium; um, ureteric mesenchyme. (D) Scheme summarizing the stratification defects and the molecular alterations with respect to KRT5, KRT14, KRT15, ΔNP63 and UPK1B expression in the development of the *Pparg-cKO* urothelium.

To define the onset and progression of these cellular defects, we compared *Pparg-cKO* and control ureters at fetal stages (E14.5, E16.5, E18.5) and at P7. Histological staining revealed a thinning of the urothelium in *Pparg-cKO* mice at P7, whereas the ureteral lumen and the mesenchymal compartment appeared unaffected at all stages analyzed (Fig. 3A,B, Fig. S3, Table S2). CDH1 expression in combination with nuclear counterstaining showed that the urothelium of *Pparg-cKO* ureters remained two-layered from E18.5 onwards (Fig. 3C). The cells in the luminal cell layer were significantly smaller than in the control at E18.5 and P7 (Fig. 3C, Fig. S4, Table S3). In the control ureter, UPK1B and UPK2 expression was activated in luminal cells at E16.5, strongly labeling S cells and more weakly labeling I cells at later stages. In the *Pparg-cKO* ureter, only a few luminal cells were positive for these two markers at P7. Expression of ΔNP63 started in the control ureter at E14.5 and continued at later stages with high levels in B cells and at lower levels in I cells. In *Pparg-cKO* mice, expression of ΔNP63 was normally activated at E14.5, but was restricted to the B cell layer from E16.5. KRT5 expression was not affected in the urothelium of the *Pparg-cKO* ureter: expression started in a few basal cells at E16.5, labeled about half of the cells in this layer at E18.5, and was found in all B cells at P7 as in the control (Fig. 3C). Expression of squamous epithelial markers (KRT6, KRT10) was not found in either control or mutant UE (Fig. S5C). Expression of both KRT14 and KRT15 was ectopically activated in B cells of the *Pparg-cKO* ureter at P7. In the control, ALDH1A2 expression was activated in suburothelial mesenchymal cells at E18.5 and homogeneously labeled the lamina propria at P7. In the *Pparg-cKO* ureter, ALDH1A2 expression was detected in only a few suburothelial mesenchymal cells at P7. Expression of SMC markers started at E16.5 and continued indistinguishably in the mesenchymal wall of control and *Pparg-cKO* ureters at E18.5 and P7 (Figs 3C, Fig. 5D). *Pparg-cKO* ureters explanted at P0 and cultured for 6 days showed no defects in the onset, frequency and intensity of contractions, (further) excluding structural and functional alterations in the peristaltic machinery in *Pparg-cKO* ureters (Fig. S6, Table S4). (Note that in this and some later experiments we used P0 ureters instead of E18.5 ureters due to a change in the German animal protection legislation.)

Given a previous report that loss of *Pparg* in the bladder urothelium affects mitochondrial structure in the luminal layer, we performed an ultrastructural analysis at E18.5. In *Pparg-cKO* ureters, the mitochondrial endowment appeared unaffected. However, consistent with impaired S cell differentiation, hinges, known to separate urothelial plaques, were absent from the surface, which was instead covered with a large number of small vesicular bodies. In addition, fusiform vesicles, a compartment for intracellular transport of urothelial plaques, were small and oval-shaped in the luminal cells of *Pparg-cKO* ureters (Fig. S7).

We conclude that in the *Pparg-cKO* ureter, the UE fails to activate S cell differentiation at E16.5 and generate I cells for further stratification from E18.5, but ectopically expresses KRT14 and KRT15 at postnatal stages (Fig. 3D, schematic). The delayed activation of ALDH1A2 suggests a non-cell-autonomous effect of epithelial loss of *Pparg* on lamina propria development.

### Apoptosis is not affected in *Pparg-cKO* ureters, but increased proliferation occurs in the basal cell layer after birth

**Fig. 4.**
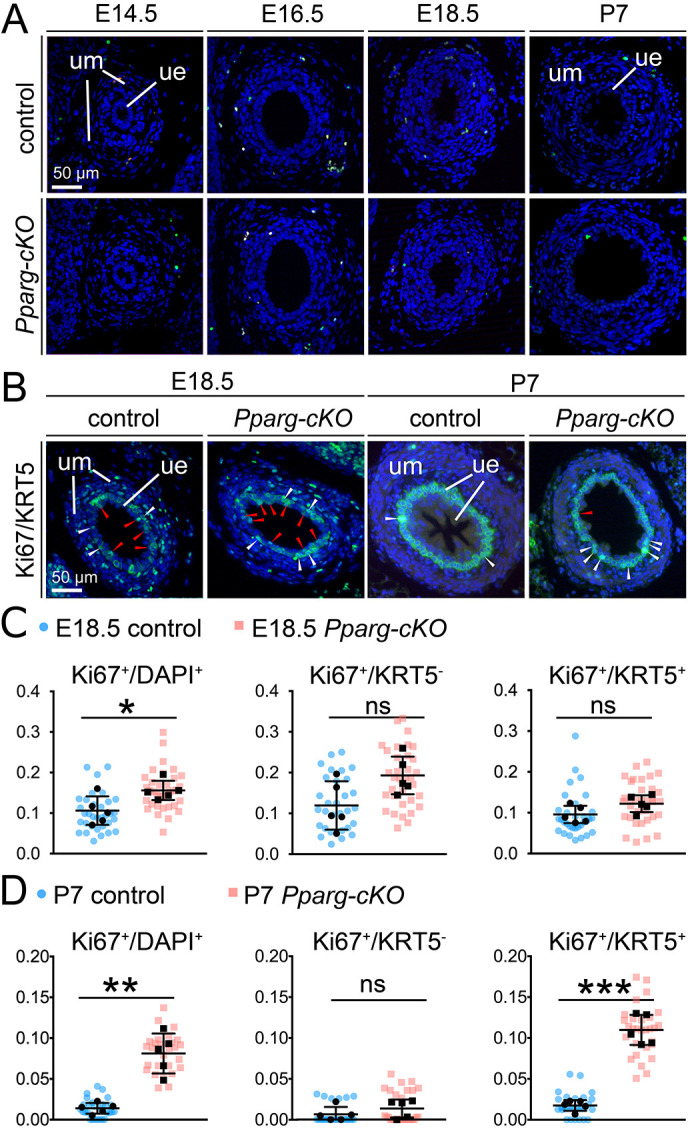
**Increased proliferation in the *Pparg-cKO* urothelium at E18.5 and P7.** (A) Immunofluorescence analysis of apoptosis (green) by the TUNEL assay on proximal ureter sections from control and *Pparg-cKO* mice. Nuclei are counterstained with DAPI (blue). *n*=3. (B) Analysis of urothelial cell proliferation by Ki67 staining (green) on transverse proximal ureter sections of E18.5 and P7 control (*n*=5) and *Pparg-cKO* (*n*=5) mice. Cells in the basal layer are marked by KRT5 expression (green); non-basal/luminal cells are KRT5^−^. Differential localization of Ki67 in the nucleus and of KRT5 in the subcortical cytoplasm allows the identification of proliferating cells in the basal layer (Ki67^+^KRT5^+^; white arrowheads) and in the non-basal/luminal cell layer (Ki67^+^KRT5^−^; red arrowheads). (C,D) Quantification of Ki67^+^ cells in basal and non-basal/luminal cell layers of E18.5 (C) and P7 (D) control and *Pparg-cKO* ureters. The ratio of Ki67^+^ cells to total DAPI^+^ cells in the urothelium, of Ki67^+^ cells to KRT5^−^ (non-basal/luminal) cells, and of Ki67^+^ to KRT5^+^ basal cells are shown. Values are expressed as mean±sd. ns, not significant; *P<0.05; **P<0.01; ***P<0.001 (two-tailed Student's *t*-test with Welch's correction). Individual sections are shown as color-coded data points (blue circles for controls; pink squares for *Pparg-cKO*). For source data and statistics, see Table S5. ue, ureteric epithelium; um, ureteric mesenchyme.

We next examined whether the cellular changes in the mutant urothelium were preceded and/or accompanied by alterations in apoptosis and/or proliferation. The terminal deoxynucleotidyl transferase dUTP nick end labeling (TUNEL) assay did not detect any changes in programmed cell death in *Pparg-cKO* ureters at fetal stages (E14.5, E16.5, E18.5) and at P7 (Fig. 4A). Proliferation was detected by the Ki67 antigen (Mki67). In the control ureter, urothelial proliferation reached approximately 10% at E18.5 and decreased to 1% at P7. In the *Pparg-cKO* urothelium, proliferation was 15% at E18.5 and 8% at P7. Increased proliferation throughout the mutant urothelium was detected at E18.5. At P7, luminal cells were quiescent in both mutant and control urothelium, but proliferation of (KRT5^+^) basal cells was greatly increased to 12% in the mutant urothelium compared to 1% in the control (Fig. 4B-D, Table S5).

### *Pparg* is required for the expression of S cell-specific genes but also for some B cell and I cell genes, including *Shh*

**Fig. 5.**
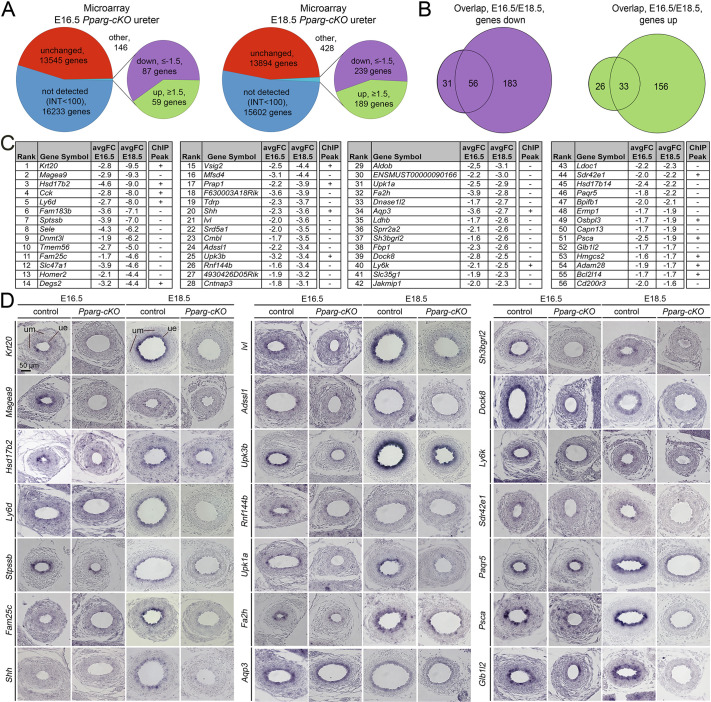
***Pparg* is required for the expression of *Shh* and many S cell-specific genes in the ureteric epithelium.** (A) Pie charts summarizing the results of the microarray analysis of E16.5 and E18.5 control and *Pparg-cKO* ureters. (B) Bio-Venn diagrams showing the overlap of genes with decreased and increased expression in the microarrays of E16.5 and E18.5 *Pparg-cKO* ureters. (C) List of 56 genes with decreased expression [fold change (FC)≤-1.5] in the microarray analysis of both E16.5 and E18.5 *Pparg-cKO* ureters. Genes are ordered by average fold change (avgFC) at E18.5. The ChIP peak column indicates the presence (+) or absence (−) of a PPARG-binding peak in a recent ChIP-Seq experiment. (D) RNA *in situ* hybridization analysis on sections of the proximal ureter from control and *Pparg-cKO* embryos at E16.5 and E18.5 for candidates from the microarray analysis shown in C. Genes are again ordered according to average fold change at E18.5. *n*≥3 for each probe, stage and genotype. ue, ureteric epithelium; um, ureteric mesenchyme.

To identify, in an unbiased manner, the molecular changes that may cause the stratification and differentiation defects in *Pparg-cKO* ureters, we performed microarray-based gene expression profiling at E16.5 and E18.5. Using an intensity threshold of 100 to reduce expression noise and fold changes of at least 1.5 to detect robust expression changes in the two individual arrays, we detected 59 genes with increased expression and 87 genes with decreased expression in mutant ureters at E16.5, and 189 genes with increased expression and 239 genes with decreased expression at E18.5 (Fig. 5A, Tables S6-S9; GSE254236, GSE254237). Assuming that the expression of PPARG-regulated genes is altered at both stages, we overlapped the two arrays and found a common set of 33 genes with increased expression and 56 genes with decreased expression (Fig. 5B, Tables S10 and S11). Functional annotation using the DAVID software tool (Huang et al., 2009) revealed an enrichment of gene ontology (GO) terms related to altered biosynthetic pathways and metabolism in both pools (Tables S12 and S13). Manual inspection of the list of upregulated genes identified major urinary proteins and keratins (*Krt4*, *Krt6a/b*). *Krt14* expression was upregulated (E16.5: +1.4; E18.5: +1.9) as well as *Krt15* (E16.5: +1.4; E18.5: +1.5). Inspection of the list of commonly downregulated genes identified several genes previously assigned to S cells, including *Krt20*, *Upk3b* and *Upk1a*, but also *Shh,* which encodes a signal restricted to B cells and I cells (Harnden et al., 1995; Yu et al., 2002, 1990) (Fig. 5C). In a recent report, Sanchez and colleagues performed chromatin immunoprecipitation with sequencing (ChIP-Seq) experiments for PPARG in cultured urothelial cells (Sanchez et al., 2021). Using this resource, we found that 17 out of the 56 commonly downregulated genes, including *Krt20* and *Shh*, were assigned peaks for PPARG binding (Fig. 5C, Fig. S8), indicating that our transcriptional profiling enriched direct targets of PPARG transcriptional activation.

To (further) dissect the cell-type specificity of PPARG function, we performed RNA *in situ* hybridization analysis for all putative direct targets and for many of the additional downregulated genes for which we were able to generate probes. Most of the candidates, including *Krt20*, *Hsd17b2*, *Ly6d*, *Sptssb*, *Fam25c* (also known as *Fam25a*), *Adssl1*, *Rnf144b*, *Upk1a*, *Sh3bgrl2*, *Sdr42e1*, *Ly6k* and *Psca,* were specifically expressed in S cells in control ureters at E16.5 and/or at E18.5 and were strongly reduced or absent in the mutant. *Ivl*, *Upk3b, Fa2h, Paqr5* and *Glb1l2* were strongly expressed in S cells and I cells in control ureters at these stages, and appeared at reduced levels in the mutant ureter. *Shh* expression in B cells and I cells was lost in the mutant. Pan-urothelial expression of *Magea9* and of *Dock8* was strongly reduced, while *Aqp3* was weakly affected in the mutant (Fig. 5D). We did not detect specific signals for *Cck*, *Ermp1*, *Jakmip1* and *Slc47a1* in control and *Pparg-cKO* ureters at E16.5 and E18.5 (Fig. S9).

Consistent with the marginal changes in the microarrays, RNA *in situ* hybridization did not detect expression changes of transcription factor genes that have previously been implicated in urothelial differentiation, such as *Elf5*, *Foxa1*, *Klf5*, *Trp63* and *Grhl3* in E16.5 and E18.5 *Pparg-cKO* ureters (Bell et al., 2011; Varley et al., 2009; Weiss et al., 2013; Wu et al., 2015; Yu et al., 2009) (Fig. S10A). GRHL3 expression was found in luminal cells of E16.5 and E18.5 *Pparg-cKO* ureters, as in the control (Fig. S10B). *Fabp4*, a direct target gene of PPARG in adipose tissue and the bladder urothelium (Liu et al., 2019; Rival et al., 2004), was not expressed in the UE of E16.5 and E18.5 control and *Pparg-cKO* embryos (Fig. S11). We conclude that *Pparg* is required for the expression of a unique set of genes in the developing urothelium of the ureter, namely *Shh* and a few other genes in the cells of the basal layer, and a large number of S cell-specific genes in the luminal cells.

### Reconstitution of SHH signaling partially rescues the cellular defects in the *Pparg-cKO* ureter

To investigate whether the loss of *Shh* expression in *Pparg-cKO* ureters translates into a reduction of SHH signaling, we analyzed the expression of *Ptch1*, a bona fide direct target of this pathway, as well as the effector gene *Aldh1a2* (Deuper et al., 2022; Ingham and McMahon, 2001; Straube et al., 2025). At E18.5 and P7, the expression of both genes was reduced in peri-urothelial mesenchymal cells, i.e. the lamina propria, in *Pparg-cKO* ureters, confirming reduction of SHH signaling (Fig. S12).

Given the important role of SHH signaling in mesenchymal and epithelial proliferation and differentiation programs in the embryonic and early fetal ureter, i.e. from E11.5 to E14.5 (Bohnenpoll et al., 2017b; Yu et al., 2002), we examined whether individual inhibition of SHH signaling would recapitulate some of the cellular changes in postnatal *Pparg-cKO* ureters. To this end, we explanted E18.5 wild-type ureters and cultured them for 6 days in the presence of the SHH signaling inhibitor cyclopamine at a concentration of 10 µM, which was previously determined to effectively inhibit SHH signaling (Bohnenpoll et al., 2017b; Incardona et al., 1998; Meuser et al., 2022; Straube et al., 2025). We observed a loss of *Ptch1* and ALDH1A2 expression, recapitulating the situation in *Pparg-cKO* ureters at E18.5 and (partially) at P7 (Fig. S13A,B). However, neither the expression of cyto-differentiation markers (ΔNP63, KRT5, UPK1B, UPK2) and KRT15 (KRT14 expression was not reliably detected in the culture setting) nor the urothelial cell number or the ratio of luminal cells, intermediate cells and basal cells was affected at the culture endpoint (Fig. S13B-D, Table S14). Urothelial proliferation was slightly decreased (Fig. S13E, Table S14). This indicates that the individual loss of SHH signaling in wild-type ureters does not affect urothelial development and/or maintenance in a major fashion at late fetal and/or early postnatal stages.

**Fig. 6.**
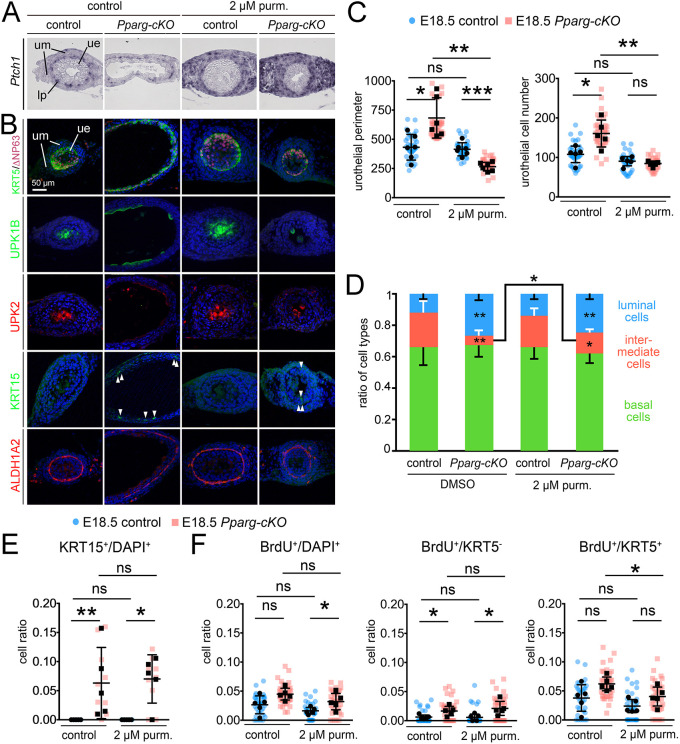
**Restoration of SHH signaling partially rescues urothelial defects in explant cultures of E18.5 *Pparg-cKO* ureters.** Control and *Pparg-cKO* ureters were explanted at E18.5 and cultured for 6 days in the absence or presence of 2 µM purmorphamine (purm.). (A) RNA *in situ* hybridization analysis on sections of the proximal ureter for expression of *Ptch1*. (B) Immunofluorescence analysis on adjacent sections for markers of B cells (KRT5), B cells and I cells (P63), S cells (UPK1B, UPK2), regenerative response (KRT15) and the lamina propria (ALDH1A2). Arrowheads indicate KRT15^+^ cells in the basal layer of the mutant ureter. (C-F) Quantification of the luminal circumference and the total urothelial cell number (C), of the ratio of luminal cells (unstained), intermediate cells (KRT5^−^P63^+^) and basal cells (KRT5^+^P63^+^) (D), of the ratio of KRT15^+^ cells to total urothelial cells as detected by DAPI staining (E) and of proliferation by the BrdU assay in total urothelial cells (BrdU^+^/DAPI^+^), in non-basal cells (BrdU^+^/KRT5^−^) and in basal cells (BrdU^+^/KRT5^+^) (F). Values are expressed as mean±sd. ns, not significant; *P<0.05, **P<0.01; ***P<0.001. A two-sided Welch's *t*-test was used for comparison of mutant results in C and D. A Mann-Whitney test was used in E. For all other data, we used a two-tailed Student's *t*-test. Individual sections are shown as color-coded data points (blue circles for controls; pink squares for *Pparg-cKO*). See Table S15 for source data and statistics. lp, lamina propria; ue, ureteric epithelium; um, ureteric mesenchyme.

We next assessed whether reconstitution of SHH signaling could rescue some of the observed cellular changes in *Pparg-cKO* ureters. To this end, we cultured E18.5 wild-type and *Pparg-cKO* ureters for 6 days in the presence of the SHH signaling agonist purmorphamine (Meuser et al., 2022; Wu et al., 2004), and performed histological and molecular analyses (Fig. 6).

Explant cultures of E18.5 *Pparg-cKO* ureters faithfully recapitulated the *in vivo* changes*. Ptch1* expression was decreased (Fig. 6A); the luminal circumference and urothelial cell number were greatly increased. The urothelium was only two-layered, lacked S cell differentiation and showed KRT15^+^ cells in the basal layer (Fig. 6B,C). Proliferation in the luminal cell layer was slightly increased. Treatment of mutant ureters with 2 µM purmorphamine led to strong *Ptch1* expression throughout the ureteric mesenchyme (Fig. 6A). Ureteral lumen, urothelial cell number and B cell proliferation were reduced to control levels. The number of I cells was increased, but the luminal (S) cell differentiation defect and KRT15 expression in the basal cell layer appeared unaltered in the mutant (Fig. 6B-F, Table S15). This indicates that loss of SHH signaling contributes to some of the cellular changes in postnatal *Pparg-cKO* ureters.

### Reconstitution of SHH signaling partially rescues the molecular changes in the *Pparg-cKO* ureter

**Fig. 7.**
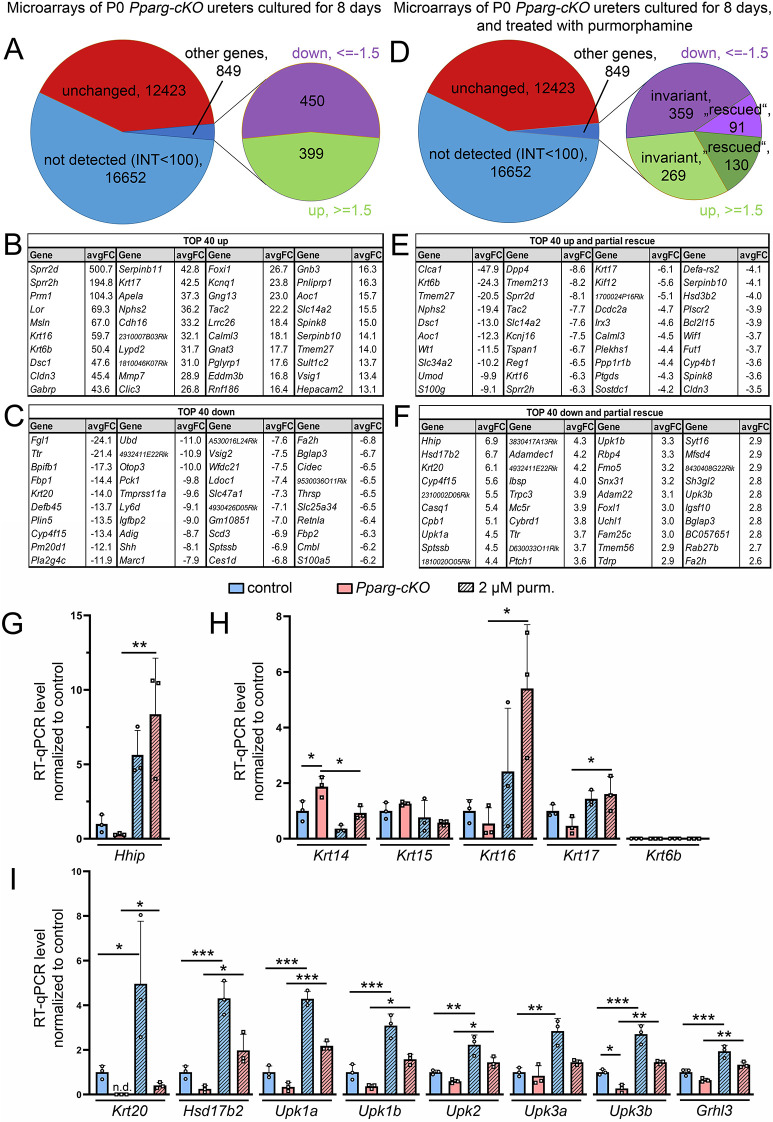
**Reconstitution of SHH signaling partially rescues the molecular changes in *Pparg-cKO* ureters.** (A-F) Control and *Pparg-cKO* ureters were explanted at P0 and cultured for 8 days with DMSO as a control (A-C) or with 2 µM purmorphamine (D-F) before transcriptional profiling by microarrays. (A,D) Pie charts summarizing the results of the microarray analyses. (B,C) List of 40 genes with strongly increased expression (B) and strongly decreased expression (C) in *Pparg-cKO* ureter explants cultured for 8 days with DMSO. (E,F) List of 40 genes, expression of which was most strongly ‘rescued’ by purmorphamine treatment both from the group of upregulated genes (E) and downregulated genes (F) in the microarray analysis of *Pparg-cKO* ureters treated with 2 µM purmorphamine. (G-I) RT-qPCR analysis for *Hhip* (G), various Krt genes (H), and S cell genes (I) in P0 control and *Pparg-cKO* ureter explants cultured for 8 days with DMSO (control) and 2 µM purmorphamine (purm.). *n*=3. Significant differences related to post-hoc test are indicated (*P<0.05; **P<0.01; ***P<0.001). For *Krt20* expression data, the Shapiro–Wilk test failed due to no detection within the *Pparg-cKO* control group. Therefore, separate evaluation of the P0 control with DMSO and 2 µM purmorphamine was performed by the two-tailed Student's *t*-test after log-normal transformation of primary data to meet the prerequisite of equal variances by *F*-test. See Table S24 for RT-qPCR source data and statistics.

To better identify the molecular programs, which are rescued by purmorphamine treatment in cultures of mutant ureters, we first profiled the transcriptional changes in 8-day cultures of P0 *Pparg-cKO* ureters by microarray analysis. Using an intensity threshold of 100 and fold changes of at least 1.5 in the two individual arrays, we detected 399 genes with increased expression and 450 genes with decreased expression in *Pparg-cKO* ureters (Fig. 7A-C, Tables S16 and S17; GSE270635). Functional annotation using DAVID (Huang et al., 2009) revealed for the pool of upregulated genes an enrichment of GO terms related to cell junctions and cell adhesion and to cornification and keratinization. In the pool of downregulated genes, numerous terms related to lipid biosynthesis were enriched (Tables S18 and S19). Notably, both pools were significantly larger than the pools of deregulated genes found for E16.5 and E18.5 *Pparg-cKO* ureters, suggesting an accumulation of secondary changes related to compensatory mechanisms to maintain tissue integrity (upregulated genes) and to the loss of terminally differentiated S cells with their enormous biosynthetic activity (downregulated genes) over time.

Administration of purmorphamine resulted in profound transcriptional changes in (wild-type and) *Pparg-cKO* ureters (Fig. 7D-F, Tables S20 and S21; GSE270635). With respect to the 399 genes with upregulated expression in *Pparg-cKO* ureters, we found that 130 of these genes showed a decrease in expression, i.e. were partially ‘rescued’. Functional annotation of this gene set revealed enrichment of the terms ‘keratinization’ and ‘cornification’ related to decreased expression of keratins such as *Krt6b*, *Krt16*, *Krt17* (Fig. 7E, Table S22).

From the set of 450 genes with decreased expression in *Pparg-cKO* ureters, we found that purmorphamine led to increased expression of 91 of them. Functional annotation for these 91 genes revealed ‘apical plasma membrane urothelial plaques’ as a term related to increased expression of Upk genes*.* Notably, other S cell genes, including *Hsd17b2*, *Krt20, Sptssb*, *Ttr*, *Fam25c* and *Rab27b* also regained some expression, suggesting that S cell-specific gene expression is controlled by PPARG both directly and in a non-cell-autonomous manner via SHH signaling (Fig. 7F, Table S23).

Because (small) expression changes are difficult to detect by RNA *in situ* hybridization on sections of cultured ureters, we validated and quantified the expression (changes) of candidate genes by reverse transcription quantitative polymerase chain reaction (RT-qPCR). This assay confirmed the expression changes of *Hhip*, a direct target of SHH signaling (Chuang et al., 2003) (Fig. 7G, Table S24A), as well as of *Krt14* and *Krt15*. For other keratins, we could not detect expression (*Krt6b*) or confirm the expression changes, possibly due to very low overall expression levels (Fig. 7H, Table S24B). In contrast, RT-qPCR confirmed the expression changes of S cell structural genes (Upk genes, *Krt20*, *Hsd17b2*) as well as of *Grhl3*, including the upregulation by purmorphamine treatment in both control and *Pparg-cKO* ureters (Fig. 7I, Table S24C).

## DISCUSSION

### PPARG regulates S cell differentiation in the ureter

Previous *in vitro* studies described that Upk gene expression depends on nuclear activation of PPARG and that PPARG is a direct upstream regulator of Upk genes (Salma et al., 2004; Varley et al., 2009, 2004b). Furthermore, in mice with a conditional (*Hoxb7-cre* mediated) loss of *Pparg* in the UE, Upk gene and *Krt20* expression was found to be significantly reduced at P1 and in adult stages (Weiss et al., 2013).

Our independent conditional approach not only confirms that *Pparg* is required for the expression of these classical S cell markers, but also shows that the entire S cell differentiation program in the mouse ureter depends on *Pparg*. In *Pparg-cKO* ureters, a luminal cell layer was formed at E16.5, but the cells remained small and did not express UPKs at fetal and postnatal stages. Our transcriptional profiling of fetal ureters revealed a large set of genes, expression of which was not activated in the luminal layer of *Pparg-cKO* ureters. Comparison with a ChIP-Seq dataset of PPARG in a bladder urothelial cancer cell line showed that a large fraction of these genes contain PPARG-binding sites (Sanchez et al., 2021). Together with the strong expression of PPARG in the luminal cell layer from E16.5 onwards, this argues that PPARG drives differentiation of S cells by directly activating the transcription of Upk genes as well as of many other genes that collectively transform the small luminal precursors into large biosynthetically highly active cells.

The expression of some S cell-specific genes, including *Sptssb*, *Upk1b*, *Upk3b* and *Fa2h*, was reduced but not absent in the *Pparg-cKO* ureter, suggesting alternative activating inputs in expression of these genes. A strong candidate is the transcription factor GRHL3. In mice with a systemic loss of *Grhl3*, the cells of the luminal layer of the bladder epithelium (the ureter was not analyzed) were small and did not express UPKs and KRT20 (Yu et al., 2009). Transcriptional profiling of the mutant bladder revealed strongly reduced expression of *Upk2* and *Snx31* (a gene involved in endosomal recycling), whereas expression of other Upk genes and S cell markers was less affected (Yu et al., 2009). In *Pparg-cKO* ureters, the expression of *Upk2* and *Snx31* was only slightly affected at E16.5 and E18.5. Importantly, the expression of *Grhl3*/GRHL3 was normal in the *Pparg-cKO* urothelium at these stages. These results suggest that PPARG and GRHL3 are independently required for S cell differentiation in the developing ureter and bladder. Some S cell-specific genes (*Upk2*, *Snx31*) may be preferentially activated by GRHL3, some by PPARG (*Krt20*, *Hsd17b2*), while others (e.g. *Upk3b*) may be co-regulated by both transcriptional activators.

In mice with a conditional loss of *Pparg* in the bladder epithelium, *Grhl3* expression has been shown to be reduced in an RNA-sequencing analysis of adult mutant bladders (Liu et al., 2019). As *Grhl3* expression has not been validated at mRNA or protein levels in adults, or at fetal stages when secondary changes can be excluded, it remains unclear whether PPARG acts upstream of *Grhl3* in the bladder urothelium.

Work in normal human urothelial (NHU) cells has identified the transcription factor genes *FOXA1* and *IRF1* as direct targets and mediators of PPARG function (Varley et al., 2009). The expression of both transcription factor genes was unchanged in *Pparg-cKO* ureters, making it unlikely that they act as functional mediators of PPARG in the mouse ureter.

Analysis of mice with a conditional loss of *Pparg* in the bladder epithelium confirmed a requirement for *Pparg* in S cell differentiation, and suggested a primary function in the transcriptional control of mitochondrial biogenesis and fatty acid transport in this process (Liu et al., 2019). In our transcriptional profiling of fetal *Pparg-cKO* ureters, we did not find genes related to mitochondrial functions. Together with our finding that *Fabp4*, a direct transcriptional target of PPARG in adipose tissue and the bladder urothelium, is not expressed in the UE, this may indicate that PPARG regulates common but also distinct targets in S cell differentiation in the bladder and ureter. However, it should be noted that the profiling of the mutant bladders was performed in adults, and not at the fetal onset of PPARG function as in our study. Therefore, the transcriptional changes in the mutant bladder may not represent primary PPARG targets, but rather the long-term alterations associated with the absence of functional S cells. Circumstantial evidence for such a notion is provided by the ChIP-Seq data set for *PPARG* in urothelial carcinoma cells, which did not identify PPARG-binding sites in genes related to fatty acid metabolism and mitochondrial function (Sanchez et al., 2021).

### Loss of *Shh* expression contributes to B cell and I cell changes in *Pparg-cKO* ureters

Our detailed phenotypic characterization of *Pparg-cKO* ureters at both fetal and postnatal stages revealed not only a lack of differentiated S cells, but also severe alterations in B cell and I cell development. Stratification of the UE into a three-layered architecture did not occur, resulting in a complete absence of I cells after birth. Cells of the basal layer continued to proliferate and ectopically expressed markers of a potential regenerative response, KRT14 (and KRT15) (Papafotiou et al., 2016; Tai et al., 2013), in postnatal life. These findings extend results from previous conditional *Pparg* knockout studies that demonstrated B cell proliferation in mutant ureters at P1, and described a lack of I cells and an expansion of KRT14^+^ cells in adult bladders, respectively (Liu et al., 2019; Weiss et al., 2013).

It is known that the complete absence of S cells and I cells after severe injury results in the activation of KRT14 expression in B cells and their subsequent proliferative expansion (Mysorekar et al., 2009; Papafotiou et al., 2016). Similar changes have been found in mice deficient for *Upk1b*, *Upk2* and *Upk3a*, which fail to form urothelial plaques (Carpenter et al., 2016; Hu et al., 2000; Kong et al., 2004). Thus, the proliferative expansion of B cells in the *Pparg-cKO* urothelium is most likely secondary to the loss of differentiated, and thus functional, S cells.

Loss of S cells and I cells in an infection model leads to a rapid restoration of urothelial architecture by dedifferentiation of B cells into I cells, and subsequent differentiation of I cells into S cells (Wiessner et al., 2022). In *Upk1b*-deficient mice, the urothelium becomes hypercellular with multiple layers of B cells and I cells (Carpenter et al., 2016). In contrast, the *Pparg-cKO* urothelium failed to generate new I cells and remained two-layered into adulthood. Increased B cell proliferation likely led to the lateral expansion of the tissue.

Our experiments provide compelling evidence that this disparate response in the *Pparg-cKO* urothelium is caused by the loss of *Shh* expression. Activation of SHH signaling in cultures of perinatal *Pparg-cKO* ureters rescued the epithelial circumference, reduced urothelial cell number, decreased B cell proliferation and, importantly, increased I cell number, indicating a shift from horizontal expansion to vertical stratification. Support for such a role for SHH signaling comes from studies in urothelial carcinoma cells, in which SHH overexpression after PPARG inhibition resulted in a partial rescue of cell proliferation and migration/invasion (Sanchez et al., 2021).

Our transcriptional profiling provides a first indication of the programs that SHH signaling may affect in this condition. We found that reactivation of SHH signaling leads to a downregulation of some keratins, which are involved in the proliferative response (*Krt14*, *Krt15*) and/or squamous differentiation (*Krt6*, etc., although this needs further validation). It seems plausible that reduced levels of these intermediate filament proteins but also of cell adhesion and polarity regulators could restore the normal physical properties of B cells, and/or lead to reduced proliferation and an altered cell division plane. However, we also found that the expression of many S cell-specific genes (such as *Krt20*, Upk genes, *Fam25c*) was increased (independently of PPARG), suggesting that these genes may receive an independent activating input from SHH signaling, possibly via *Grhl3* upregulation. These findings complement previous reports that active SHH signaling limits tumor progression in the bladder (Kim et al., 2019; Shin et al., 2011), and suggest that restoration of SHH signaling may be a therapeutic option in urothelial carcinomas with inactivated *PPARG*.

Because urothelial SHH exclusively acts in the surrounding mesenchyme (Bohnenpoll et al., 2017b; Yu et al., 2002), it is likely that a mesenchymal signal transduces its function back to the urothelium. Based on the loss of ALDH1A2 expression in E18.5 *Pparg-cKO* ureters and reports that mesenchymal BMP4 mediates SHH signaling in the early ureter (Bohnenpoll et al., 2017b), retinoic acid and BMP4 are interesting candidates for such a relay function.

Complete pharmacological inhibition of SHH signaling in perinatal ureter cultures affected lamina propria formation, as recently reported (Straube et al., 2025), but did not result in urothelial differentiation defects and slightly decreased rather than increased B cell proliferation. This suggests that SHH signaling is not individually required for perinatal urothelial development and homeostasis. In bladder injury and infections as well as in *Upk1b*-deficient bladder urothelium, *Shh* expression is strongly upregulated (Carpenter et al., 2016; Kim et al., 2019). This suggests that signals from differentiated S cells normally limit *Shh* expression in B cells. Such signals may also prevent the dedifferentiation of B cells into I cells in homeostasis. In the absence of such inhibitory signals, i.e. when S cells are absent or non-functional, SHH signaling is enhanced and participates in the regenerative response.

We conclude that PPARG affects urothelial development and integrity in at least two ways. PPARG induces S cell differentiation from E16.5 onward by directly activating a large number of genes in the luminal cell layer. PPARG also directly activates *Shh* expression in B cells. In development, SHH signaling is possibly required for formation of the intermediate cell layer after E16.5. After injury and infection, enhanced SHH signaling attenuates the proliferative expansion of B cells, supports the formation of I cells, and enhances the differentiation of S cells, i.e. is part of the regenerative response to restore urothelial integrity.

### Ureter dilatation in adult *Pparg-cKO* mice

Similar to a previously reported conditional knockout model (Weiss et al., 2013), *Pparg-cKO* mice developed progressive bilateral ureteral dilatation with parenchymal thinning by P40. The hydroureter, as this condition is known, was not caused by SMC or pacemaker defects, as the expression of SMC markers was normal and peristalsis was unaffected in the perinatal stages. We also found no evidence of physical obstruction along the ureter and the vesicoureteric junction as a possible cause.

In ‘classical’ hydroureter, the accumulation of urine leads to a uniform expansion of the luminal circumference and tissue thinning. In *Pparg-cKO* ureters, the luminal circumference was uneven and folded, arguing against a fluid-mediated dilatation. Since the expression of numerous keratins and basal cell proliferation was greatly increased postnatally, it is possible that proliferative urothelial expansion is a cause and not a consequence of the luminal dilatation. However, lack of differentiated S cells may contribute to dilatation by disrupting urothelial integrity, widening of the ureteral orifice and causing vesicoureteral reflux, as observed in mice lacking Upk gene expression (Carpenter et al., 2016; Hu et al., 2000; Kong et al., 2004).

## MATERIALS AND METHODS

### Mouse strains and husbandry

For conditional deletion of *Pparg* in the UE, we combined the transgenic cre driver line *Tg(Pax2-cre)1AKis* (synonym: *Pax2-cre*) (Trowe et al., 2011) with a floxed allele of *Pparg* (*Pparg^tm2Rev^* ; synonym: *Pparg^fl^*). In this allele, *loxP* sites flank exons 2 and 3 of the *Pparg* gene (reference transcript: NM011146.3), which results in the generation of an N-terminally truncated non-functional PPARG protein after cre-mediated deletion (He et al., 2003). The mice were maintained on an NMRI outbred background. *Pax2-cre/+;Pparg^fl/fl^* (*Pparg-cKO*) mice were obtained from matings of *Pax2-cre/+;Pparg^f/l+^* males and *Pparg^fl/fl^* females; cre-negative littermates served as controls. Embryos for *Pparg* expression analyses were obtained from matings of NMRI wild-type mice.

Pregnancies were timed as E0.5 by vaginal plugs in the morning after mating. Pregnant females were sacrificed by cervical dislocation. Embryos and urogenital systems were dissected in PBS. Specimens were fixed in 4% paraformaldehyde in PBS, transferred to methanol and stored at −20°C prior to further processing. PCR genotyping was performed on genomic DNA prepared from biopsies (Table S25A).

Mice were housed in light- and temperature-controlled rooms at the central animal facility of the Hannover Medical School. The experiments were performed in accordance with the German Animal Welfare Legislation (§4, TierSchG). They were approved by the local Institutional Animal Care and Research Advisory Committee and authorized by the Lower Saxony State Office for Consumer Protection and Food Safety (reference number 42500/1H).

### Organ cultures

Ureters for explant cultures were dissected in L-15 Leibovitz medium (F1315, Biochrom) and cultured on 0.4 µm PET membrane ThinCert inserts (657641, Greiner Bio-One) at 37°C in 5% CO_2_ in organ culture medium (DMEM/F12) (21331-020, Thermo Fisher Scientific) supplemented with 100 units/ml penicillin, 100 μg/ml streptomycin (15140-122, Thermo Fisher Scientific), 1 mM sodium pyruvate (11360-039, Thermo Fisher Scientific), 1× MEM NEAA (11140-035, Thermo Fisher Scientific) and 1× GlutaMAX (35050-038, Thermo Fisher Scientific) at the air–liquid interface. Medium was changed every other day. For pharmacological manipulation of SHH signaling, we used cyclopamine (S1146, Selleck Chemicals GmbH) dissolved in DMF at a final concentration of 10 µM, and purmorphamine (540220, Merck) dissolved in DMSO at a final concentration of 2 µM. At the end of the culture period, the tissue was prefixed in methanol, 4% paraformaldehyde in PBS, transferred in methanol, and stored at −20°C prior to further processing.

### Histological, immunofluorescence and immunohistochemical analyses

Embryos, urogenital systems and explants were embedded in paraffin wax and sectioned at 5 µm. Hematoxylin/Eosin and Sirius Red staining were performed according to standard procedures. For antigen detection, primary antibody labeling was performed overnight at 4°C after antigen retrieval (5 min at 121°C; H-3300, Vector Laboratories), blocking of endogenous peroxidases with 6% H_2_O_2_ for 30 min, and incubation in blocking buffer provided from the kits (TNB) for 30 min. Primary antibodies were visualized with either biotinylated Fab fragments or fluorophore-conjugated secondary antibodies (Table S26). The TSA Tetramethylrhodamine Amplification Kit (1:100; NEL701001KT, PerkinElmer) was used for antibody amplification. Cell nuclei were counterstained with 4′,6-diamidino-2-phenylindole (DAPI; 6335.1, Carl Roth) according to the manufacturer's instructions. DAB substrate solution (NEL938001EA, PerkinElmer) was used for immunohistochemical detection of PPARG.

### Electron microscopy and toluidin staining on semi-thin sections

Electron microscopy was performed on Epon-embedded sections as described previously (Rudat et al., 2014).

### Cell proliferation and apoptosis assays

Cell proliferation rates in explant cultures (*n*=5 per condition and genotype) were assessed by the detection of incorporated 5-bromo-2'-deoxyuridine (BrdU) on 5 μm paraffin wax sections according to published protocols (Bussen et al., 2004). Explant cultures were grown for 3 h with 3.3 μg/ml BrdU in the culture medium. For each specimen, five sections of the proximal ureter were evaluated. The BrdU labeling index was defined as the number of BrdU-positive nuclei relative to the total number of nuclei as detected by DAPI counterstaining of the ureter. Data were expressed as mean±s.d.

Cell proliferation rates *in vivo* (*n*=5 per condition and genotype) were assessed by immunofluorescence detection of Ki67 on 5 μm paraffin wax sections. For each specimen, *n*=5 sections of the proximal ureter were evaluated. The Ki67 labeling index was defined as the number of Ki67-positive nuclei relative to the total number of nuclei as detected by DAPI counterstaining of the ureter. Data were expressed as mean±s.d.

Tissue apoptosis was assessed by TUNEL assay using the ApopTag Plus Fluorescein *In Situ* Apoptosis Detection Kit (S7111, Merck KGaA) on 5 μm paraffin wax sections from at least three specimens per genotype.

### RNA *in situ* hybridization analysis

RNA *in situ* hybridization analysis on 10-µm-thick paraffin wax sections using digoxigenin-labeled anti-sense probes was performed as described (Moorman et al., 2001). Primers for PCR amplification of new DNA templates for *in vitro* transcription of RNA probes are listed in Table S25B.

### Microarray experiments and data analysis

Total RNA was extracted from pools of ureters dissected from E16.5 (*n*≥9) and E18.5 (*n*≥11) sex-sorted control and *Pparg-cKO* embryos using the peqGOLD RNAPure reagent (732-3312, Peqlab). Total RNA was extracted from pools of sex-sorted P0 control and *Pparg-cKO* ureters cultured for 8 days with or without 2 µM purmorphamine using the RNeasy kit (QIAGEN) according to the manufacturer's recommendations. All RNA pools were sent to the Research Core Unit Transcriptomics of Hannover Medical School where labeled cRNA was synthesized and hybridized to the ‘Whole Mouse Genome Oligo Microarray V2’ (AMADID 026655, Agilent Technologies). Differentially expressed genes for each stage were identified by filtering normalized expression data using Excel (Microsoft Corp.) based on an intensity threshold of 100 and a 1.5-fold change. Microarray data were submitted to Gene Expression Omnibus under accession numbers GSE254236, GSE254237 and GSE270635.

For prediction of direct target genes of PPARG, we used data available in the Gene Expression Omnibus database (GSE166803) for 5637 urothelial carcinoma cells analyzed by chromatin-immunoprecipitation followed by deep sequencing for PPARG and IgG controls to investigate genome-wide PPARG occupancy (Sanchez et al., 2021). Genes upregulated in our microarray were manually compared in the Integrative Genomics Viewer [version 2.3.37(45); Robinson et al., 2011] with ChIP sequencing data (in human, hg38) based on the gene symbol.

### RT-qPCR

RNA extraction and RT-qPCR analysis for gene expression was performed on three independent pools of five ureters each of P0 control and *Pparg-cKO* ureter explants cultured for 8 days in presence of 0.1% DMSO (control) or 2 µM purmorphamine, as previously described (Bohnenpoll et al., 2017b; Incardona et al., 1998; Meuser et al., 2022; Straube et al., 2025). Primers are listed in Table S25C.

### Image documentation

Sections were photographed on a Leica DM5000 microscope using a Leica DFC300FX digital camera. Whole-mounts were photographed on a Leica M420 with a Fujix HC-300Z digital camera. All images were assembled into figures using Adobe Photoshop CS4.

### Statistics

Quantification of specific cell types and proliferating cells was performed on transverse proximal ureter sections, and marker-positive cells were counted within the urothelium. Nuclei were counter-stained with DAPI. Measurements and cell counts were performed using Fiji/ImageJ 2.3.0 (NIH) (Schindelin et al., 2012) and Adobe Photoshop CS4 software.

The circumference of the ureteral lumen was measured on scaled pictures with the Fiji/ImageJ line tool. The luminal cell area was measured on scaled pictures with the Fiji/ImageJ freehand selection tool, based on DAPI and CDH1 immunofluorescence staining (basolateral borders plus autofluorescence of the cell soma).

To determine the onset of ureter peristalsis, the presence of a peristaltic wave along the ureter was judged within a 1-min time interval. For quantification of the peristaltic frequency, the number of peristaltic waves along the ureter was counted within a 1-min time interval. For the assessment of contraction intensity of cultured ureters, 60 s videos of the explants were recorded with five frames per second on a Leica DM6000 microscope and Leica K3M digital camera and analyzed using Fiji/ImageJ 2.3.0 (NIH) (Schindelin et al., 2012). Contraction intensity was analyzed at mid ureter length by calculating the relative width of the ureter during contraction and relaxation. GraphPad Prism7 and Numbers version 6.1 were used to plot the respective graphs.

Cell proliferation rates were assessed by immunofluorescence detection for BrdU (culture) or Ki67 (*in vivo*). Cells were manually counted using the counting tool in Adobe Photoshop CS4. The labeling index was defined as the number of BrdU/Ki67-positive nuclei relative to the total number of nuclei as detected by DAPI counterstaining of the urothelium (total urothelium), KRT5^+^ cells (basal cells), KRT5^−^P63^+^ cells (intermediate cells), unstained cells (luminal cell nuclei) or KRT15^+^ cells (KRT15^+^ cells). All data were expressed as mean±s.d.

Statistical analyses of cell counts were performed using GraphPad Prism7 and Numbers version 6.1. The normal distribution of the data was tested using the Shapiro–Wilk normality test (normal distribution if *P*≥0.05); equality of variances was tested using the *F*-test (equal variances if *P*≥0.05). To compare two groups with normal distribution and equal variances, an unpaired, two-sided *t*-test was applied. The two-sided Welch's *t*-test was used if two groups with normal distribution had unequal variances. The two-sided Mann–Whitney *U*-test was used to compare two groups with non-parametric distribution. Results were expressed as mean±s.d. All significant differences are indicated (*P<0.05; **P<0.01; ***P<0.001; ****P<0.0001).

RT-qPCR data were evaluated using GraphPad Prism 8.4.37. Normality and equality of variances were assessed using the Shapiro–Wilk test and two-way ANOVA followed by Tukey's post-hoc test. Values are presented as arithmetic mean±s.d. *P*<0.05 was considered statistically significant. For details, see figure legends.

## Supplementary Material



10.1242/develop.204324_sup1 Supplementary information

Table S1. Genotype frequencies of animals derived from the breeding of *Pax2-cre/+;PParg^fl/+^* males and *Pparg^fl/fl^* females follows a Mendelian ratio.

Table S2. Quantification of the circumference of the tubular lumen in control and *Pparg-cKO* ureters at E18.5, P7 and P40.

Table S3. Quantification of the size of cells in the luminal cell layer in control and *Pparg-cKO* ureters at E18.5, P7 and P40.

Table S4. Statistics on the peristaltic activity of explants of P0 control and *Pparg-cKO* ureters cultured for 6 days.

Table S5. Cell proliferation analysis in the urothelium of control and *Pparg-cKO* ureters at E18.5 and P7.

Table S6. Genes with increased expression in microarrays of *Pparg-cKO* ureters at E16.5.

Table S7. Genes with decreased expression in microarrays of *Pparg-cKO* ureters at E16.5.

Table S8. Genes with increased expression in microarrays of *Pparg-cKO* ureters at E18.5.

Table S9. Genes with decreased expression in microarrays of *Pparg-cKO* ureters at E18.5.

Table S10. Overlap of genes with increased expression in *Pparg-cKO* ureters at E16.5 and E18.5.

Table S11. Overlap of genes with decreased expression in *Pparg-cKO* ureters at E16.5 and E18.5.

Table S12. Functional annotations of genes with increased expression in microarrays of both E16.5 and E18.5 *Pparg-cKO* ureters.

Table S13. Functional annotations of genes with decreased expression in microarrays of both E16.5 and E18.5 *Pparg-cKO* ureters.

Table S14. Inhibition of SHH signaling in E18.5+6d ureter explant cultures does not affect urothelial development.

Table S15. Restoration of SHH signaling partially rescues urothelial defects in explant cultures of E18.5+6d *Pparg-cKO* ureters.

Table S16. Genes with increased expression in microarrays of P0 *Pparg-cKO* ureters cultured for 8 days.

Table S17. Genes with decreased expression in microarrays of P0 *Pparg-cKO* ureters cultured for 8 days.

Table S18. Functional annotations of genes with increased expression in microarrays of P0 *Pparg-cKO* ureters cultured for 8 days.

Table S19. Functional annotations of genes with increased expression in microarrays of P0 *Pparg-cKO* ureters cultured for 8 days.

Table S20. Genes with increased expression in microarrays of P0 *Pparg-cKO* ureters cultured for 8 days that show responsiveness to purmorphamine treatment.

Table S21. Genes with decreased expression in microarrays of P0 *Pparg-cKO* ureters cultured for 8 days that show responsiveness to purmorphamine treatment.

Table S22. Functional annotations of genes with increased expression in microarrays of P0 *Pparg-cKO* ureters cultured for 8 days that show responsiveness to purmorphamine treatment.

Table S23. Functional annotations of genes with decreased expression in microarrays of P0 *Pparg-cKO* ureters cultured for 8 days that show responsiveness to purmorphamine treatment.

Table S24. RT-qPCR analysis of gene expression.

Table S25. List of primers used in this study.

Table S26. List of primary and secondary antibodies used in this study.

## References

[DEV204324C1] Arrighi, S. (2015). The urothelium: anatomy, review of the literature, perspectives for veterinary medicine. *Ann. Anat.* 198, 73-82. 10.1016/j.aanat.2014.11.001 25533627

[DEV204324C2] Bell, S. M., Zhang, L., Mendell, A., Xu, Y., Haitchi, H. M., Lessard, J. L. and Whitsett, J. A. (2011). Kruppel-like factor 5 is required for formation and differentiation of the bladder urothelium. *Dev. Biol.* 358, 79-90. 10.1016/j.ydbio.2011.07.020 21803035 PMC3180904

[DEV204324C3] Blanquart, C., Barbier, O., Fruchart, J. C., Staels, B. and Glineur, C. (2003). Peroxisome proliferator-activated receptors: regulation of transcriptional activities and roles in inflammation. *J. Steroid Biochem. Mol. Biol.* 85, 267-273. 10.1016/S0960-0760(03)00214-0 12943712

[DEV204324C4] Bohnenpoll, T., Feraric, S., Nattkemper, M., Weiss, A.-C., Rudat, C., Meuser, M., Trowe, M.-O. and Kispert, A. (2017a). Diversification of cell lineages in ureter development. *J. Am. Soc. Nephrol.* 28, 1792-1801. 10.1681/ASN.2016080849 28028137 PMC5461796

[DEV204324C5] Bohnenpoll, T., Wittern, A. B., Mamo, T. M., Weiss, A.-C., Rudat, C., Kleppa, M.-J., Schuster-Gossler, K., Wojahn, I., Ludtke, T. H.-W., Trowe, M.-O. et al. (2017b). A SHH-FOXF1-BMP4 signaling axis regulating growth and differentiation of epithelial and mesenchymal tissues in ureter development. *PLoS Genet.* 13, e1006951. 10.1371/journal.pgen.1006951 28797033 PMC5567910

[DEV204324C6] Bussen, M., Petry, M., Schuster-Gossler, K., Leitges, M., Gossler, A. and Kispert, A. (2004). The T-box transcription factor Tbx18 maintains the separation of anterior and posterior somite compartments. *Genes Dev.* 18, 1209-1221. 10.1101/gad.300104 15155583 PMC415645

[DEV204324C7] Carpenter, A. R., Becknell, M. B., Ching, C. B., Cuaresma, E. J., Chen, X., Hains, D. S. and McHugh, K. M. (2016). Uroplakin 1b is critical in urinary tract development and urothelial differentiation and homeostasis. *Kidney Int..* 89, 612-624. 10.1016/j.kint.2015.11.017 26880456 PMC4757817

[DEV204324C8] Castillo-Martin, M., Domingo-Domenech, J., Karni-Schmidt, O., Matos, T. and Cordon-Cardo, C. (2010). Molecular pathways of urothelial development and bladder tumorigenesis. *Urol. Oncol.* 28, 401-408. 10.1016/j.urolonc.2009.04.019 20610278

[DEV204324C9] Chuang, P.-T., Kawcak, T. N. and McMahon, A. P. (2003). Feedback control of mammalian Hedgehog signaling by the Hedgehog-binding protein, Hip1, modulates Fgf signaling during branching morphogenesis of the lung. *Genes Dev.* 17, 342-347. 10.1101/gad.1026303 12569124 PMC195990

[DEV204324C10] Dalghi, M. G., Montalbetti, N., Carattino, M. D. and Apodaca, G. (2020). The urothelium: life in a liquid environment. *Physiol. Rev.* 100, 1621-1705. 10.1152/physrev.00041.2019 32191559 PMC7717127

[DEV204324C11] Desvergne, B. and Wahli, W. (1999). Peroxisome proliferator-activated receptors: nuclear control of metabolism. *Endocr. Rev.* 20, 649-688. 10.1210/edrv.20.5.0380 10529898

[DEV204324C12] Deuper, L., Meuser, M., Thiesler, H., Jany, U. W. H., Rudat, C., Hildebrandt, H., Trowe, M.-O. and Kispert, A. (2022). Mesenchymal FGFR1 and FGFR2 control patterning of the ureteric mesenchyme by balancing SHH and BMP4 signaling. *Development* 149, dev200767. 10.1242/dev.200767 36094016

[DEV204324C13] Gandhi, D., Molotkov, A., Batourina, E., Schneider, K., Dan, H., Reiley, M., Laufer, E., Metzger, D., Liang, F., Liao, Y. et al. (2013). Retinoid signaling in progenitors controls specification and regeneration of the urothelium. *Dev. Cell* 26, 469-482. 10.1016/j.devcel.2013.07.017 23993789 PMC4024836

[DEV204324C14] Guan, Y., Zhang, Y., Davis, L. and Breyer, M. D. (1997). Expression of peroxisome proliferator-activated receptors in urinary tract of rabbits and humans. *Am. J. Physiol. Cell Physiol.* 273, F1013-F1022. 10.1152/ajprenal.1997.273.6.F1013 9435691

[DEV204324C15] Harnden, P., Allam, A., Joyce, A. D., Patel, A., Selby, P. and Southgate, J. (1995). Cytokeratin 20 expression by non-invasive transitional cell carcinomas: potential for distinguishing recurrent from non-recurrent disease. *Histopathology* 27, 169-174. 10.1111/j.1365-2559.1995.tb00025.x 8835265

[DEV204324C16] He, W., Barak, Y., Hevener, A., Olson, P., Liao, D., Le, J., Nelson, M., Ong, E., Olefsky, J. M. and Evans, R. M. (2003). Adipose-specific peroxisome proliferator-activated receptor gamma knockout causes insulin resistance in fat and liver but not in muscle. *Proc. Natl. Acad. Sci. USA* 100, 15712-15717. 10.1073/pnas.2536828100 14660788 PMC307633

[DEV204324C17] Hu, P., Deng, F.-M., Liang, F.-X., Hu, C.-M., Auerbach, A. B., Shapiro, E., Wu, X.-R., Kachar, B. and Sun, T.-T. (2000). Ablation of uroplakin III gene results in small urothelial plaques, urothelial leakage, and vesicoureteral reflux. *J. Cell Biol.* 151, 961-972. 10.1083/jcb.151.5.961 11085999 PMC2174354

[DEV204324C18] Huang, D. W., Sherman, B. T. and Lempicki, R. A. (2009). Systematic and integrative analysis of large gene lists using DAVID Bioinformatics Resources. *Nat. Protoc.* 4, 44-57. 10.1038/nprot.2008.211 19131956

[DEV204324C19] Incardona, J. P., Gaffield, W., Kapur, R. P. and Roelink, H. (1998). The teratogenic Veratrum alkaloid cyclopamine inhibits sonic hedgehog signal transduction. *Development* 125, 3553-3562. 10.1242/dev.125.18.3553 9716521

[DEV204324C20] Ingham, P. W. and McMahon, A. P. (2001). Hedgehog signaling in animal development: paradigms and principles. *Genes Dev.* 15, 3059-3087. 10.1101/gad.938601 11731473

[DEV204324C21] Jain, S., Pulikuri, S., Zhu, Y., Qi, C., Kanwar, Y. S., Yeldandi, A. V., Rao, M. S. and Reddy, J. K. (1998). Differential expression of the peroxisome proliferator-activated receptor gamma (PPARgamma) and its coactivators steroid receptor coactivator-1 and PPAR-binding protein PBP in the brown fat, urinary bladder, colon, and breast of the mouse. *Am. J. Pathol.* 153, 349-354. 10.1016/S0002-9440(10)65577-0 9708794 PMC1852994

[DEV204324C22] Kawakami, S., Arai, G., Hayashi, T., Fujii, Y., Xia, G., Kageyama, Y. and Kihara, K. (2002). PPARgamma ligands suppress proliferation of human urothelial basal cells in vitro. *J. Cell. Physiol.* 191, 310-319. 10.1002/jcp.10099 12012326

[DEV204324C23] Kim, S. E., Kim, Y., Kong, J. H., Kim, E., Choi, J. H., Yuk, H. D., Lee, H. S., Kim, H.-R., Lee, K.-H., Kang, M. et al. (2019). Epigenetic regulation of mammalian Hedgehog signaling to the stroma determines the molecular subtype of bladder cancer. *eLife* 8, e43024. 10.7554/eLife.43024 31036156 PMC6597241

[DEV204324C24] Kong, X.-T., Deng, F.-M., Hu, P., Liang, F.-X., Zhou, G., Auerbach, A. B., Genieser, N., Nelson, P. K., Robbins, E. S., Shapiro, E. et al. (2004). Roles of uroplakins in plaque formation, umbrella cell enlargement, and urinary tract diseases. *J. Cell Biol.* 167, 1195-1204. 10.1083/jcb.200406025 15611339 PMC2172608

[DEV204324C25] Liu, C., Tate, T., Batourina, E., Truschel, S. T., Potter, S., Adam, M., Xiang, T., Picard, M., Reiley, M., Schneider, K. et al. (2019). Pparg promotes differentiation and regulates mitochondrial gene expression in bladder epithelial cells. *Nat. Commun.* 10, 4589. 10.1038/s41467-019-12332-0 31597917 PMC6785552

[DEV204324C26] Meuser, M., Deuper, L., Rudat, C., Aydoğdu, N., Thiesler, H., Zarnovican, P., Hildebrandt, H., Trowe, M.-O. and Kispert, A. (2022). FGFR2 signaling enhances the SHH-BMP4 signaling axis in early ureter development. *Development* 149, dev200021. 10.1242/dev.200021 35020897

[DEV204324C27] Moorman, A. F. M., Houweling, A. C., de Boer, P. A. J. and Christoffels, V. M. (2001). Sensitive nonradioactive detection of mRNA in tissue sections: novel application of the whole-mount in situ hybridization protocol. *J. Histochem. Cytochem.* 49, 1-8. 10.1177/002215540104900101 11118473

[DEV204324C28] Mysorekar, I. U., Isaacson-Schmid, M., Walker, J. N., Mills, J. C. and Hultgren, S. J. (2009). Bone morphogenetic protein 4 signaling regulates epithelial renewal in the urinary tract in response to uropathogenic infection. *Cell Host Microbe* 5, 463-475. 10.1016/j.chom.2009.04.005 19454350 PMC2696285

[DEV204324C29] Nakashiro, K.-I., Hayashi, Y., Kita, A., Tamatani, T., Chlenski, A., Usuda, N., Hattori, K., Reddy, J. K. and Oyasu, R. (2001). Role of peroxisome proliferator-activated receptor gamma and its ligands in non-neoplastic and neoplastic human urothelial cells. *Am. J. Pathol.* 159, 591-597. 10.1016/S0002-9440(10)61730-0 11485917 PMC1850548

[DEV204324C30] Papafotiou, G., Paraskevopoulou, V., Vasilaki, E., Kanaki, Z., Paschalidis, N. and Klinakis, A. (2016). KRT14 marks a subpopulation of bladder basal cells with pivotal role in regeneration and tumorigenesis. *Nat. Commun.* 7, 11914. 10.1038/ncomms11914 27320313 PMC4915139

[DEV204324C31] Rival, Y., Stennevin, A., Puech, L., Rouquette, A., Cathala, C., Lestienne, F., Dupont-Passelaigue, E., Patoiseau, J.-F., Wurch, T. and Junquéro, D. (2004). Human adipocyte fatty acid-binding protein (aP2) gene promoter-driven reporter assay discriminates nonlipogenic peroxisome proliferator-activated receptor gamma ligands. *J. Pharmacol. Exp. Ther.* 311, 467-475. 10.1124/jpet.104.068254 15273253

[DEV204324C32] Robinson, J. T., Thorvaldsdóttir, H., Winckler, W., Guttman, M., Lander, E. S., Getz, G. and Mesirov, J. P. (2011). Integrative genomics viewer. *Nat. Biotechnol.* 29, 24-26. 10.1038/nbt.1754 21221095 PMC3346182

[DEV204324C33] Rochel, N., Krucker, C., Coutos-Thévenot, L., Osz, J., Zhang, R., Guyon, E., Zita, W., Vanthong, S., Hernandez, O. A., Bourguet, M. et al. (2019). Recurrent activating mutations of PPARγ associated with luminal bladder tumors. *Nat. Commun.* 10, 253. 10.1038/s41467-018-08157-y 30651555 PMC6335423

[DEV204324C34] Rudat, C., Grieskamp, T., Röhr, C., Airik, R., Wrede, C., Hegermann, J., Herrmann, B. G., Schuster-Gossler, K. and Kispert, A. (2014). Upk3b is dispensable for development and integrity of urothelium and mesothelium. *PLoS ONE* 9, e112112. 10.1371/journal.pone.0112112 25389758 PMC4229118

[DEV204324C35] Salma, N., Xiao, H., Mueller, E. and Imbalzano, A. N. (2004). Temporal recruitment of transcription factors and SWI/SNF chromatin-remodeling enzymes during adipogenic induction of the peroxisome proliferator-activated receptor gamma nuclear hormone receptor. *Mol. Cell. Biol.* 24, 4651-4663. 10.1128/MCB.24.11.4651-4663.2004 15143161 PMC416408

[DEV204324C36] Sanchez, D. J., Missiaen, R., Skuli, N., Steger, D. J. and Simon, M. C. (2021). Cell-intrinsic tumorigenic functions of PPARγ in bladder urothelial carcinoma. *Mol. Cancer Res.* 19, 598-611. 10.1158/1541-7786.MCR-20-0189 33431608 PMC8026526

[DEV204324C37] Schindelin, J., Arganda-Carreras, I., Frise, E., Kaynig, V., Longair, M., Pietzsch, T., Preibisch, S., Rueden, C., Saalfeld, S., Schmid, B. et al. (2012). Fiji: an open-source platform for biological-image analysis. *Nat. Methods* 9, 676-682. 10.1038/nmeth.2019 22743772 PMC3855844

[DEV204324C38] Shin, K., Lee, J., Guo, N., Kim, J., Lim, A., Qu, L., Mysorekar, I. U. and Beachy, P. A. (2011). Hedgehog/Wnt feedback supports regenerative proliferation of epithelial stem cells in bladder. *Nature* 472, 110-114. 10.1038/nature09851 21389986 PMC3676169

[DEV204324C39] Straube, P., Beckers, A., Jany, U. W. H., Bergmann, F., Lüdtke, T. H.-W., Rudat, C., Trowe, M.-O., Peters, I., Klopf, M. G., Mamo, T. M. et al. (2025). Interplay of SHH, WNT and BMP4 signaling regulates the development of the lamina propria in the murine ureter. *Development*, 152, dev.204214. 10.1242/dev.204214 PMC11829765 39817691

[DEV204324C40] Tai, G., Ranjzad, P., Marriage, F., Rehman, S., Denley, H., Dixon, J., Mitchell, K., Day, P. J. R. and Woolf, A. S. (2013). Cytokeratin 15 marks basal epithelia in developing ureters and is upregulated in a subset of urothelial cell carcinomas. *PLoS ONE* 8, e81167. 10.1371/journal.pone.0081167 24260555 PMC3832456

[DEV204324C41] Tate, T., Xiang, T., Wobker, S. E., Zhou, M., Chen, X., Kim, H., Batourina, E., Lin, C.-S., Kim, W. Y., Lu, C. et al. (2021). Pparg signaling controls bladder cancer subtype and immune exclusion. *Nat. Commun.* 12, 6160. 10.1038/s41467-021-26421-6 34697317 PMC8545976

[DEV204324C42] Trowe, M.-O., Maier, H., Petry, M., Schweizer, M., Schuster-Gossler, K. and Kispert, A. (2011). Impaired stria vascularis integrity upon loss of E-cadherin in basal cells. *Dev. Biol.* 359, 95-107. 10.1016/j.ydbio.2011.08.030 21925491

[DEV204324C43] Varga, T., Czimmerer, Z. and Nagy, L. (2011). PPARs are a unique set of fatty acid regulated transcription factors controlling both lipid metabolism and inflammation. *Biochim. Biophys. Acta* 1812, 1007-1022. 10.1016/j.bbadis.2011.02.014 21382489 PMC3117990

[DEV204324C44] Varley, C. L., Stahlschmid, T. J., Smith, B., Stower, M. and Southgate, J. (2004a). Activation of peroxisome proliferator-activated receptor-gamma reverses squamous metaplasia and induces transitional differentiation in normal human urothelial cells. *Am. J. Pathol.* 164, 1789-1798. 10.1016/S0002-9440(10)63737-6 15111325 PMC1615665

[DEV204324C45] Varley, C. L., Stahlschmidt, J., Lee, W.-C., Holder, J., Diggle, C., Selby, P. J., Trejdosiewicz, L. K. and Southgate, J. (2004b). Role of PPARgamma and EGFR signalling in the urothelial terminal differentiation programme. *J. Cell Sci.* 117, 2029-2036. 10.1242/jcs.01042 15054105

[DEV204324C46] Varley, C. L., Garthwaite, M. A. E., Cross, W., Hinley, J., Trejdosiewicz, L. K. and Southgate, J. (2006). PPARgamma-regulated tight junction development during human urothelial cytodifferentiation. *J. Cell. Physiol.* 208, 407-417. 10.1002/jcp.20676 16688762 PMC1522040

[DEV204324C47] Varley, C. L., Bacon, E. J., Holder, J. C. and Southgate, J. (2009). FOXA1 and IRF-1 intermediary transcriptional regulators of PPARgamma-induced urothelial cytodifferentiation. *Cell Death Differ.* 16, 103-114. 10.1038/cdd.2008.116 18688264

[DEV204324C48] Weiss, R. M., Guo, S., Shan, A., Shi, H., Romano, R.-A., Sinha, S., Cantley, L. G. and Guo, J. K. (2013). Brg1 determines urothelial cell fate during ureter development. *J. Am. Soc. Nephrol.* 24, 618-626. 10.1681/ASN.2012090902 23449535 PMC3609140

[DEV204324C49] Wiessner, G. B., Plumber, S. A., Xiang, T. and Mendelsohn, C. L. (2022). Development, regeneration and tumorigenesis of the urothelium. *Development* 149, dev198184. 10.1242/dev.198184 35521701 PMC10656457

[DEV204324C50] Wu, X., Walker, J., Zhang, J., Ding, S. and Schultz, P. G. (2004). Purmorphamine induces osteogenesis by activation of the hedgehog signaling pathway. *Chem. Biol.* 11, 1229-1238. 10.1016/j.chembiol.2004.06.010 15380183

[DEV204324C51] Wu, B., Cao, X., Liang, X., Zhang, X., Zhang, W., Sun, G. and Wang, D. (2015). Epigenetic regulation of Elf5 is associated with epithelial-mesenchymal transition in urothelial cancer. *PLoS ONE* 10, e0117510. 10.1371/journal.pone.0117510 25629735 PMC4309403

[DEV204324C52] Yu, J., Manabe, M., Wu, X. R., Xu, C., Surya, B. and Sun, T. T. (1990). Uroplakin I: a 27-kD protein associated with the asymmetric unit membrane of mammalian urothelium. *J. Cell Biol.* 111, 1207-1216. 10.1083/jcb.111.3.1207 1697295 PMC2116275

[DEV204324C53] Yu, J., Carroll, T. J. and McMahon, A. P. (2002). Sonic hedgehog regulates proliferation and differentiation of mesenchymal cells in the mouse metanephric kidney. *Development* 129, 5301-5312. 10.1242/dev.129.22.5301 12399320

[DEV204324C54] Yu, Z., Mannik, J., Soto, A., Lin, K. K. and Andersen, B. (2009). The epidermal differentiation-associated Grainyhead gene Get1/Grhl3 also regulates urothelial differentiation. *EMBO J.* 28, 1890-1903. 10.1038/emboj.2009.142 19494835 PMC2711180

