# Supplementary material for: PPARG contributes to urothelial integrity in the murine ureter by activating the expression of Shh and superficial cell-specific genes
Source: Development. 2025 Apr 17;152(8):dev204324. doi: 10.1242/dev.204324 (PMC12045629; doi:10.1242/dev.204324)
Supplement: Supplementary information [file develop-152-204324-s1.pdf]

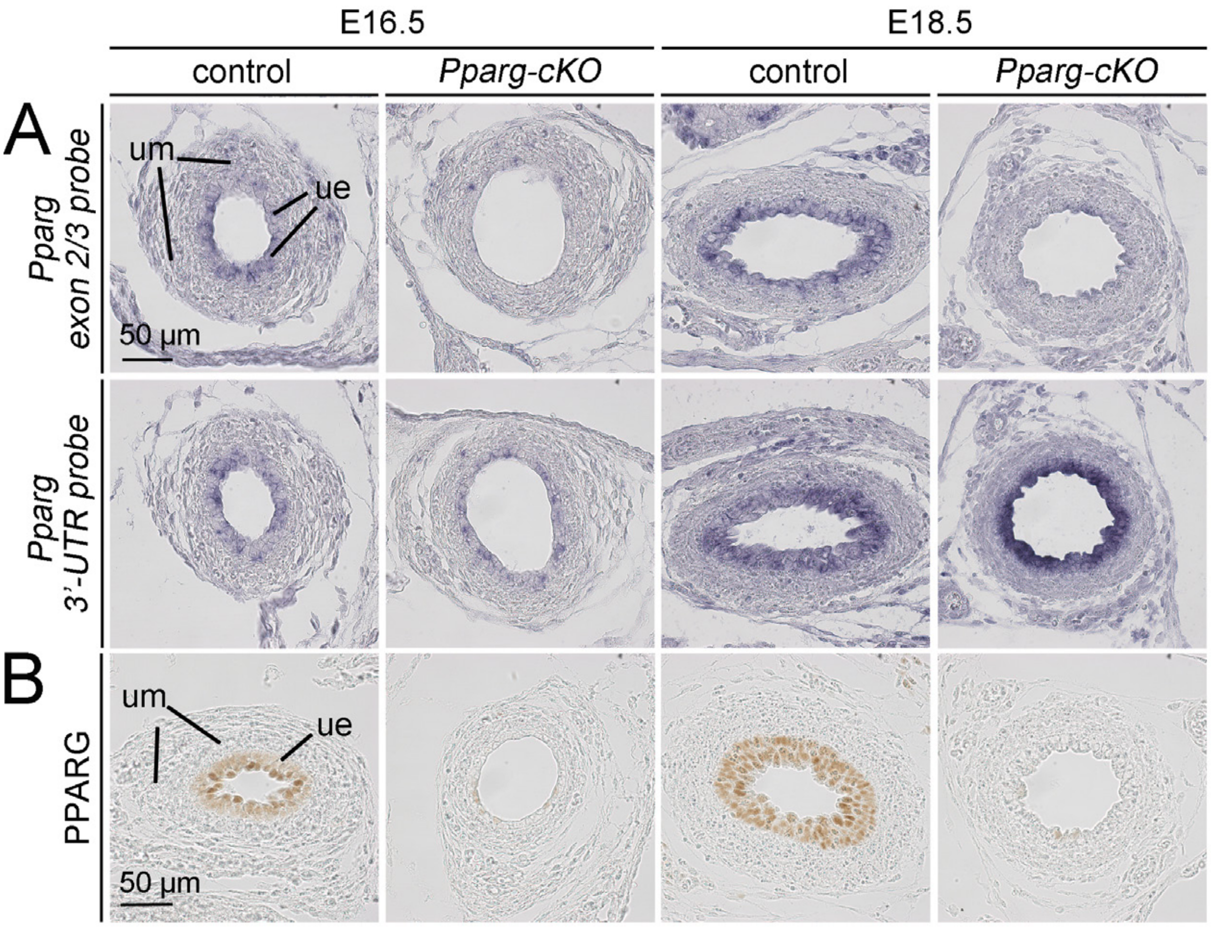

Fig. S1. The *Pax2-cre* driver line mediates conditional deletion of PPARG in the ureteric epithelium. (A) RNA *in situ* hybridization analysis of *Pparg* expression on transverse sections of the proximal ureter region of control and *Pparg-cKO* ureters at E16.5 and E18.5 using probes against exon2/3 of the *Pparg* mRNA and the 3'-untranslated region (UTR) (reference: NM011146.3). Note that the exon2/3 probe does not detect a message in the ureteric epithelium of *Pparg-cKO* embryos at E16.5 and E18.5, whereas the 3'-UTR probe detects a mutant *Pparg* mRNA with an increased expression in the mutant at E18.5. This indicates that a stable *Pparg* transcript lacking the exon2/3 region is upregulated in the mutant urothelium at this stage. (B) Immunohistochemical analysis of PPARG protein on transverse sections of the proximal region of control and *Pparg-cKO* ureters at E16.5 and E18.5 shows that no functional PPARG protein is made in the mutant. *n*=8 for each stage, genotype and assay. ue, ureteric epithelium; um, ureteric mesenchyme.

Development • Supplementary information

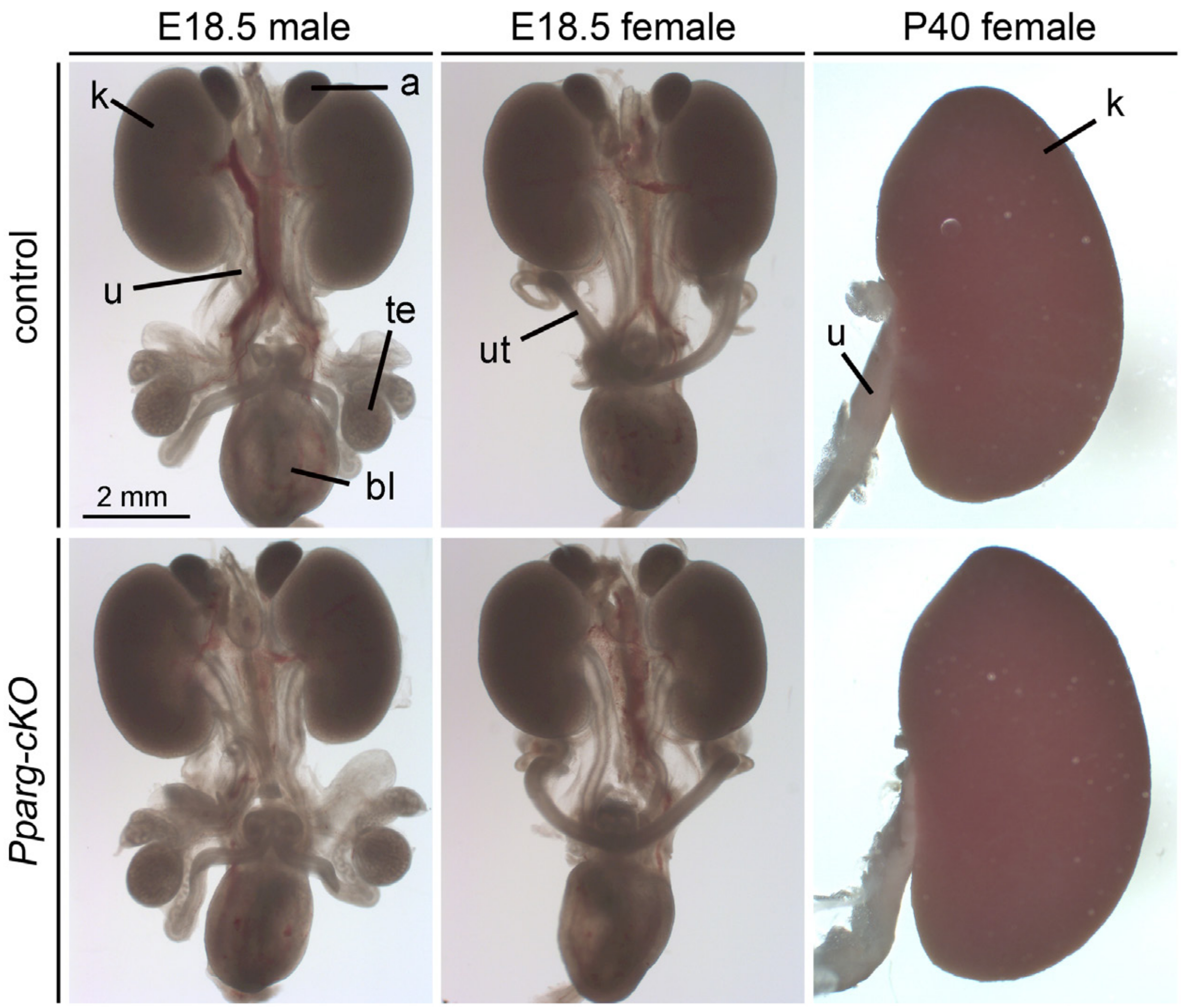

Fig. S2. The urogenital system of *Pparg-cKO* mice is morphologically unaffected. Morphology of a whole urogenital system of an E18.5 male and female control and *Pparg-cKO* embryo, and of a kidney with ureter of a female P40 control and *Pparg-cKO* mouse. $n \geq 3$ for all stages and genotypes. a, adrenal; bl, bladder; k, kidney; te, testis; u, ureter; ut, uterus.

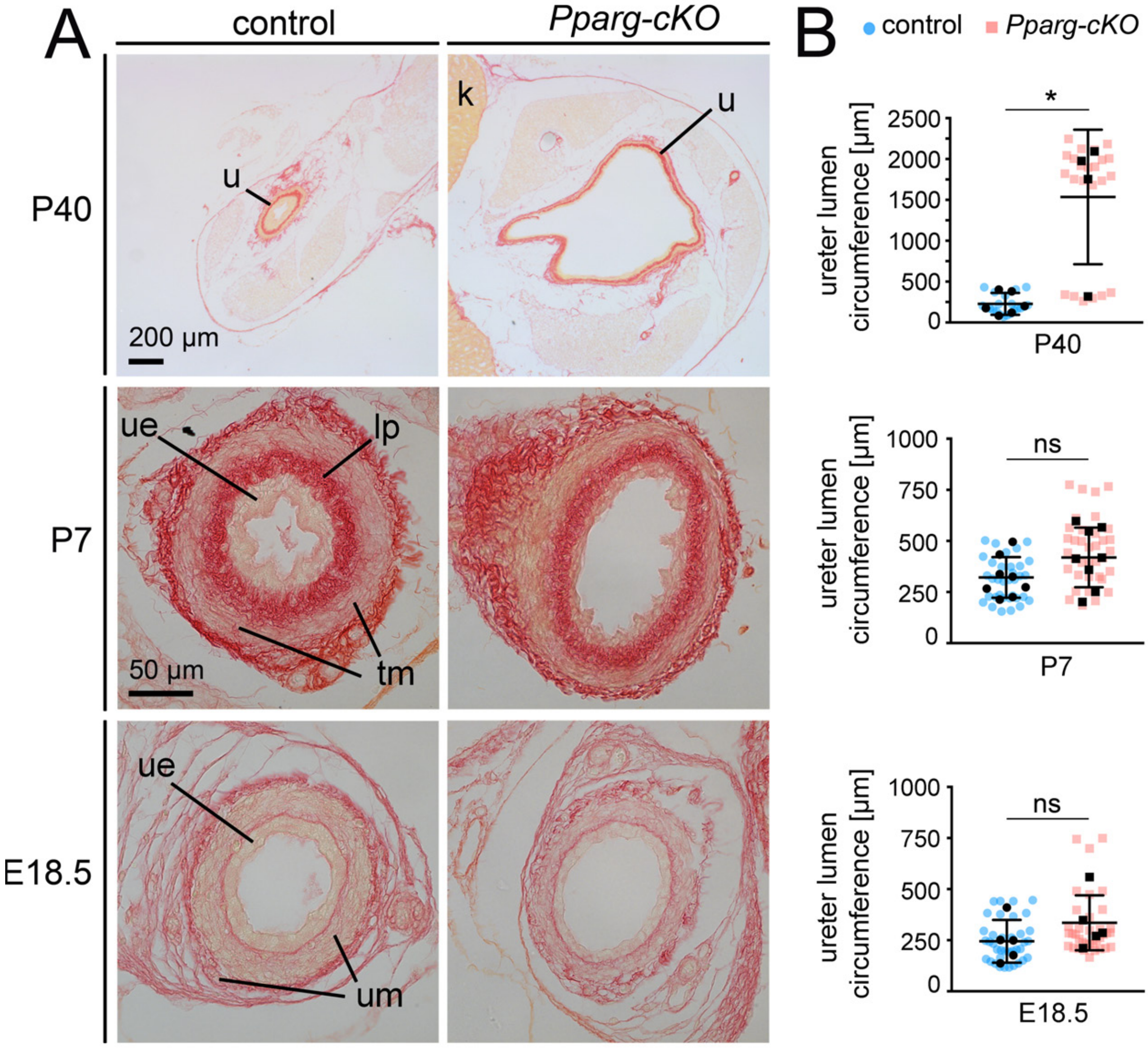

Fig. S3. The luminal circumference is increased in P40 *Pparg-cKO* ureters. (A,B) Histological analysis by Sirius Red staining (A) and quantification of the circumference of the ureteral lumen in µm (B). The luminal circumference is significantly increased at P40 but not at E18.5 and P7 in *Pparg-cKO* ureters. Values are expressed as mean±sd. Two-tailed Student's t-test or two-sided Welch's t-test. ns, non-significant; **P*<0.05. Individual sections are presented as color-coded data points (blue dots for controls, red squares for *Pparg-cKO*). For source data and statistics see Table S2. k, kidney; lp, lamina propria; tm, tunica muscularis; u, ureter; ue, ureteric epithelium; um, ureteric mesenchyme.

Development • Supplementary information

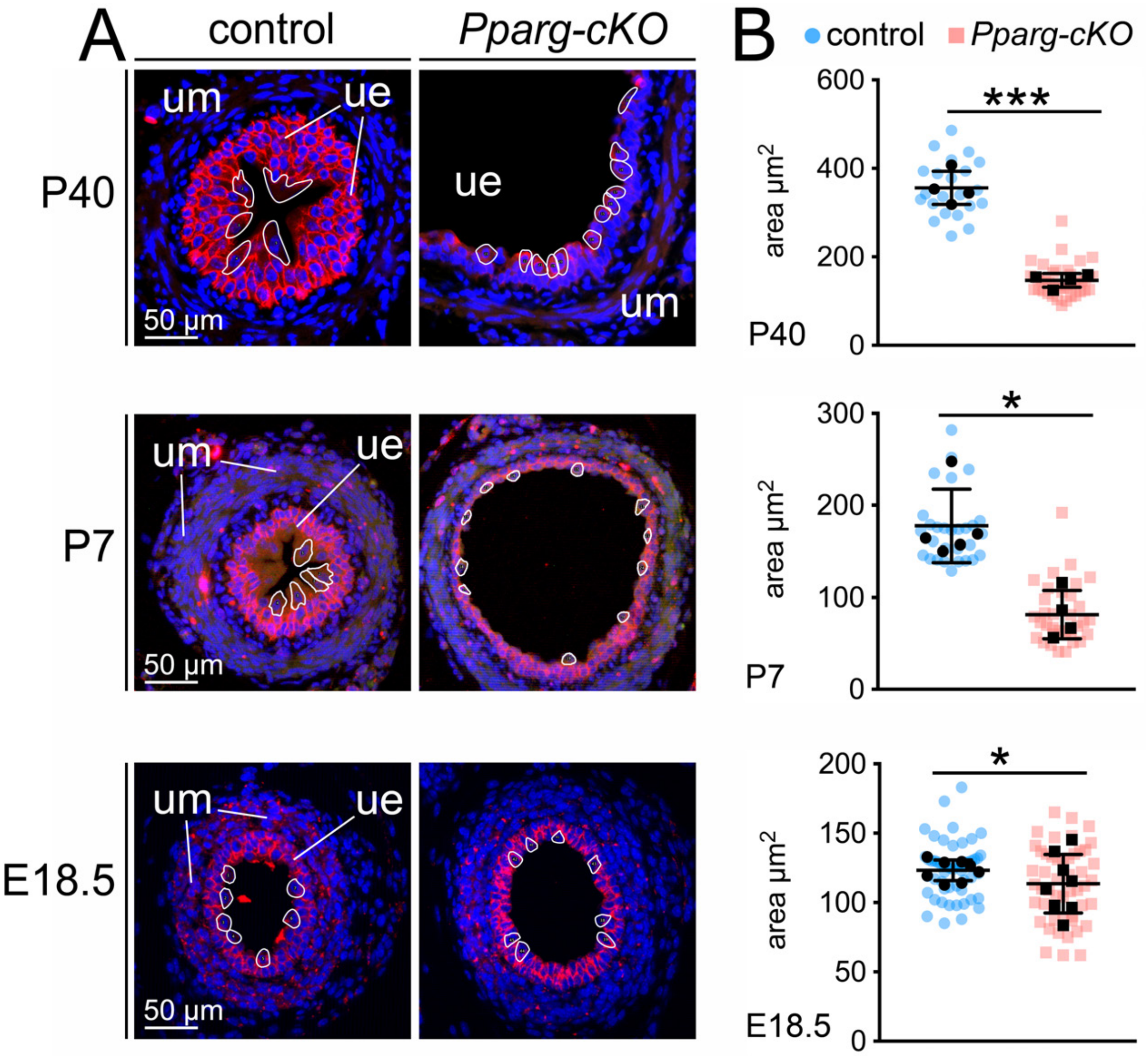

Fig. S4. The size of luminal cells is decreased in *Pparg-cKO* ureters at E18.5, P7 and P40. (A,B) Immunofluorescence analysis on sections of the proximal ureter region for CDH1 (A), and quantification of the area of luminal cells as a surrogate for size (B). White lines encircle the circumference of luminal cells as detected by staining of CDH1 in the basolateral membrane and background fluorescence of the cytoplasm. The size of luminal cells is significantly decreased in *Pparg-cKO* ureters at P40, P7 and E18.5. Values are expressed as mean±sd. Two-tailed Student's t-test, or two-sided Welch's t-test, or Mann-Whitney test. **P*<0.05; ****P*<0.001. Individual areas are presented as color-coded data points (blue dots for controls, red squares for *Pparg-cKO*). For source data and statistics see Table S3. ue, ureteric epithelium; um, ureteric mesenchyme.

Development • Supplementary information

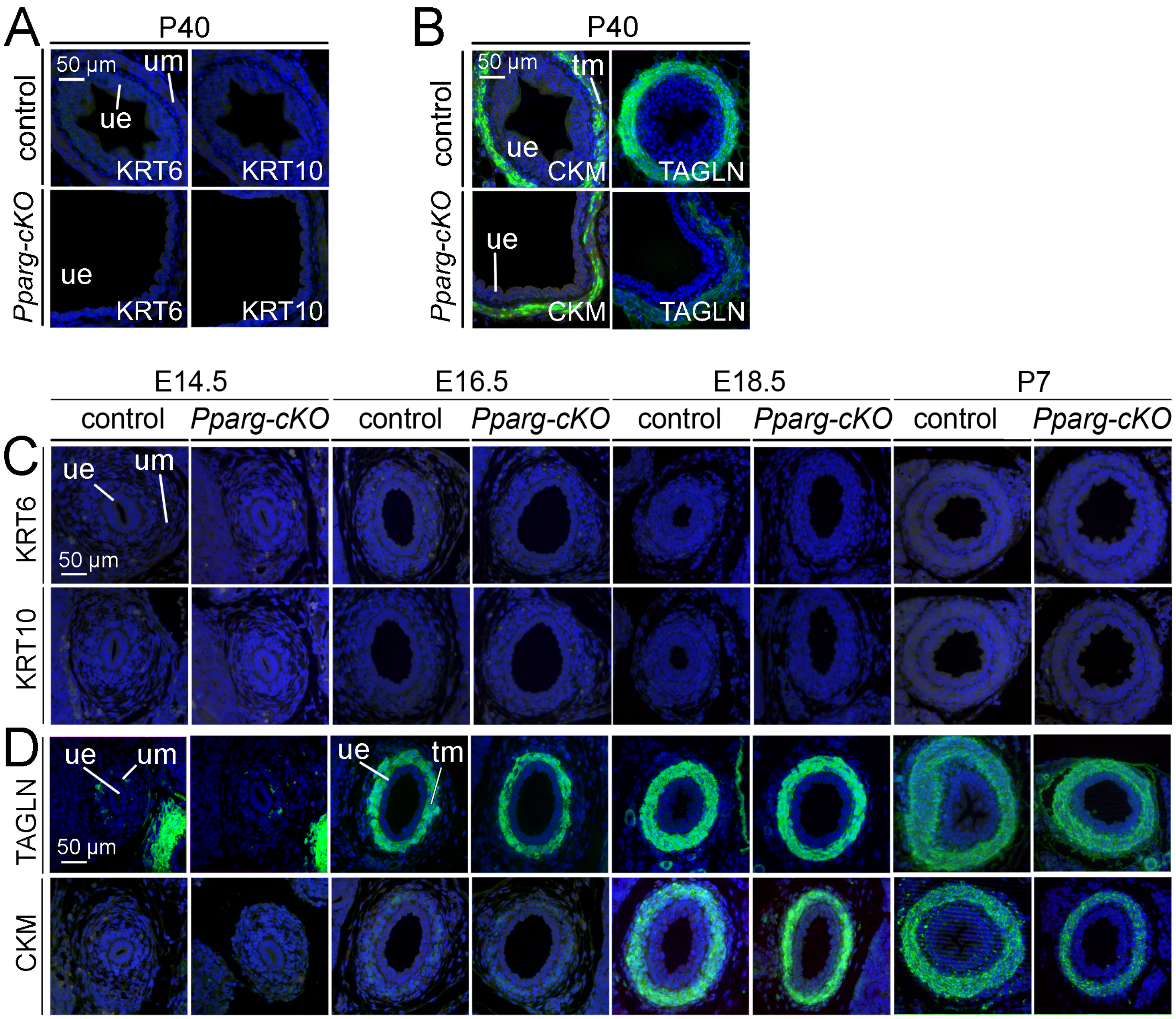

Fig. S5. Expression of squamous epithelial and smooth muscle cell markers is not changed in *Pparg-cKO* ureters during development. (A-D) Immunofluorescence analysis of the squamous epithelial markers KRT6 and KRT10 (A,C) and of the smooth muscle cell markers CKM and TAGLN (B,D) on transverse sections of the proximal ureter of control and *Pparg-cKO* embryos at P40 (A,B) and at E14.5, E16.5, E18.5 and P7 (C,D). $n \geq 3$ for each stage, genotype and probe. tm, tunica muscularis; ue, ureteric epithelium; um, ureteric mesenchyme.

Development • Supplementary information

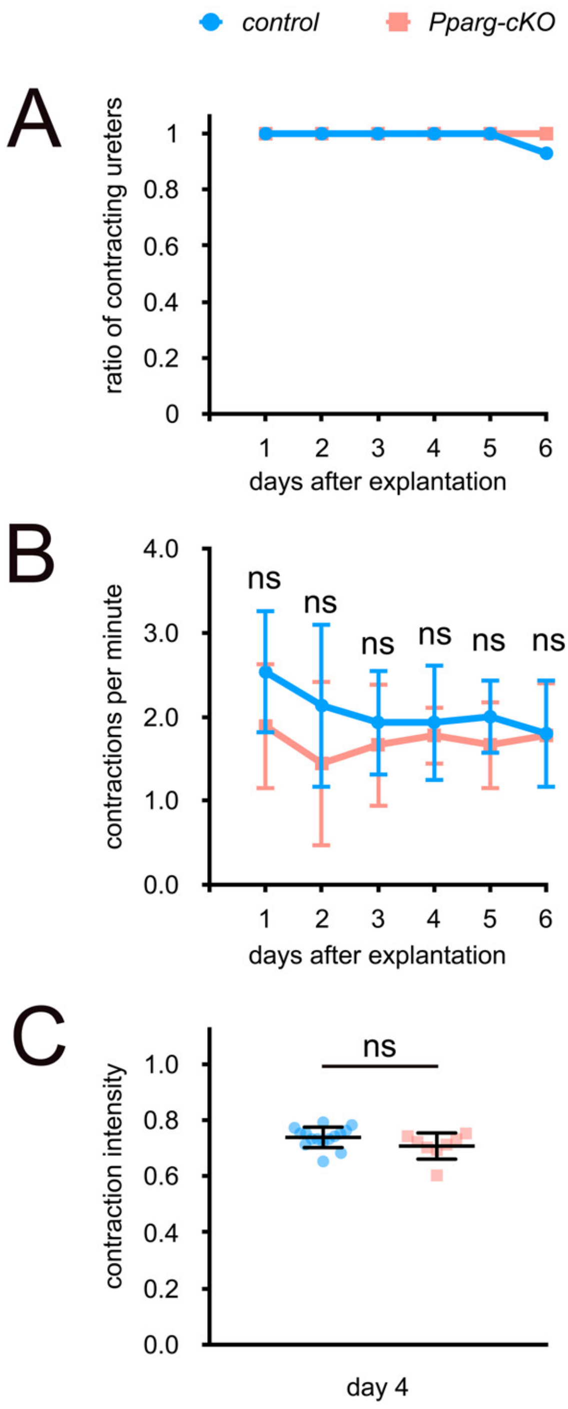

Fig. S6. The peristaltic activity is unaltered in explant cultures of P0 *Pparg-cKO* ureters. Control (n=14) and *Pparg-cKO* (n=7) ureters were explanted at P0 and cultured for 6 days. (A) Graph showing the ratio of contracting to non-contracting ureters at the indicated time-points of the culture. (B) Graph of the contraction frequency (per min). Values are shown as mean±s.d. (C) Quantification of the contraction intensity at culture day 4. Values are expressed as mean±sd. Mann-Whitney test. ns, non-significant. Individual ureters are presented as color-coded data points (blue dots for controls, red squares for *Pparg-cKO*). For source data and statistics see Table S4.

Development • Supplementary information

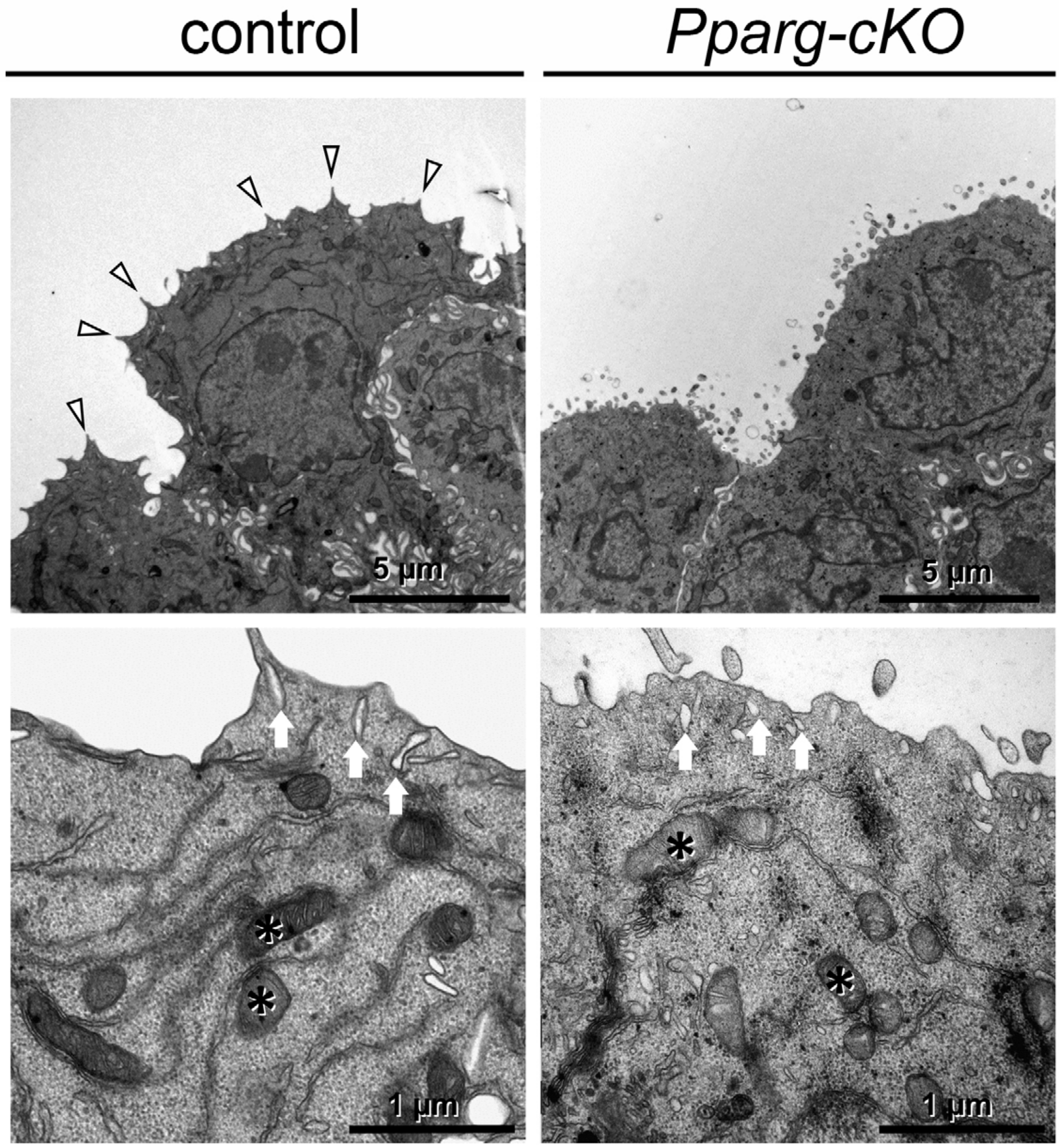

Fig. S7. The cells of the luminal cell layer in *Pparg-cKO* ureters do not show mitochondrial defects. Transmission electron microscopy of the ureter of an E18.5 control and *Pparg-cKO* embryo. In the control ureter, hinges protrude from the apical surface (arrowheads, upper images), separating urothelial plaques. Fusiform vesicles are found underneath the plasma membrane (white arrows, lower images). In most of the luminal cells in the *Pparg-cKO* ureter, the hinges are absent and membrane vesicles cover the surface. The fusiform vesicles are smaller and oval-shaped. The mitochondrial endowment appears unaffected (lower images, asterisks).

Development • Supplementary information

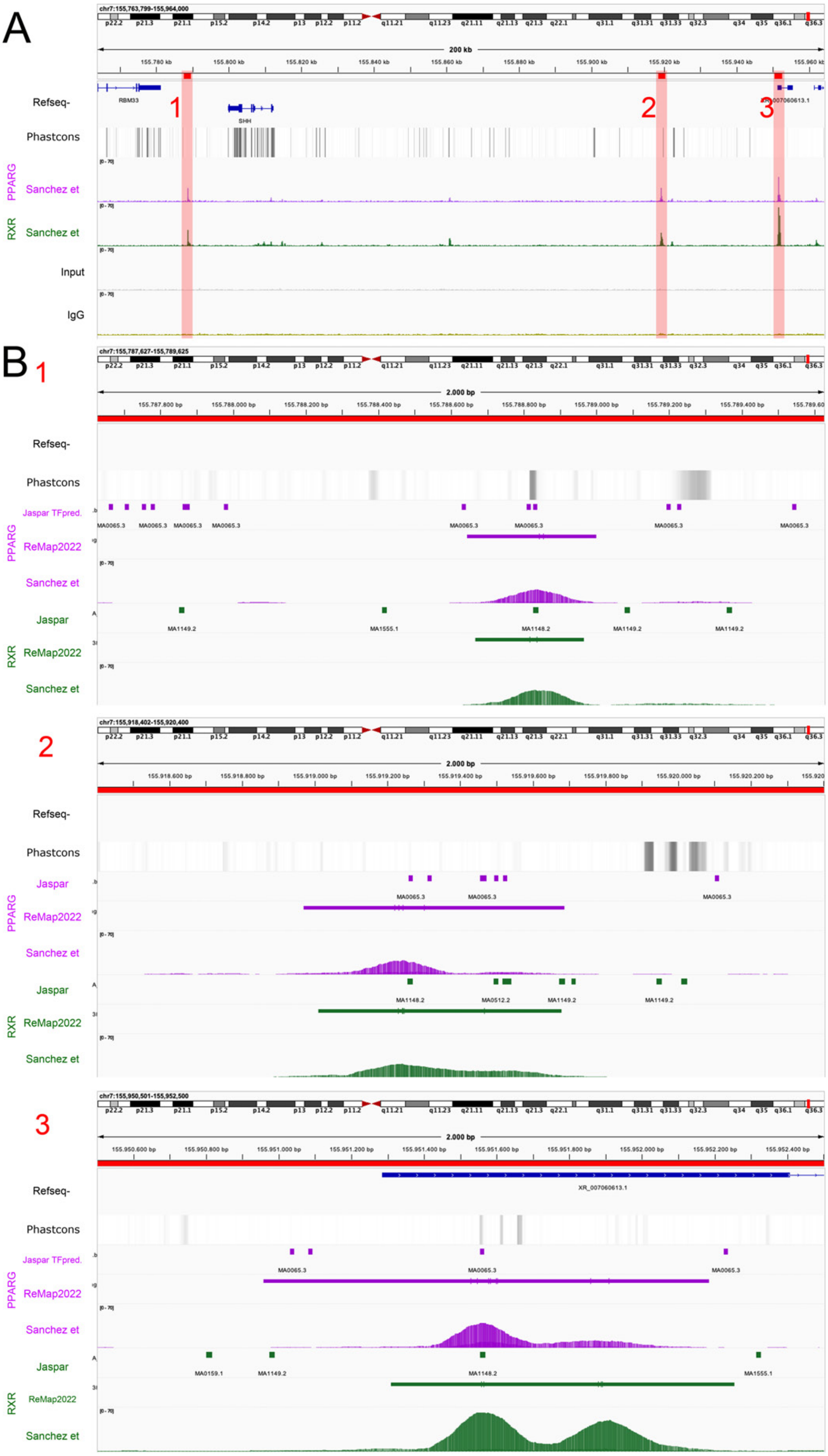

Development • Supplementary information

Fig. S8. PPARG binding sites in a genomic region encompassing the *Shh* locus. (A) Shown is a 200 kbp genomic region containing the *Shh* locus with the intron-exon structure of Refseq genes in blue. Red boxes highlight three regions of around 2 kbp that contain binding peaks for PPARG and RXR. The DNA conservation of this region between twenty species was visualized as a density plot (PhastCons 20 way). The ChIP sequencing results by Sanchez et al. are shown in purple bar graphs for PPARG and in green for RXR. The input control lane is shown in grey and the IgG control in yellow. The data range of the y-axis for the ChIP sequencing results is always scaled to 70 reads maximum. (B) Higher magnification of the 2 kbp regions of the red boxes (1,2,3) shown in (A). The DNA conservation of this region between twenty species was visualized as a density plot (PhastCons 20 way). Predicted Jaspar transcription factor binding motives (Jaspar TFpred) for PPARG in this region are shown as purple rectangles and the respective Matrix ID is indicated. Comparison to available public PPARG ChIP-seq data was performed by using the ReMap Atlas of regulatory regions (ReMap2022) labelling the identified peak regions with purple bars. The ChIP sequencing results by Sanchez et al. are shown in purple bar graphs for PPARG. Predicted Jaspar transcription factor binding motives (Jaspar TFpred) for RXR in this region are shown as green rectangles and the respective Matrix ID is indicated. Comparison to available public RXR ChIP-seq data was performed by the ReMap Atlas of regulatory regions (ReMap2022) labelling the identified peak regions with green bars. The ChIP sequencing results by Sanchez et al. are shown in green bar graphs for RXR.

Development • Supplementary information

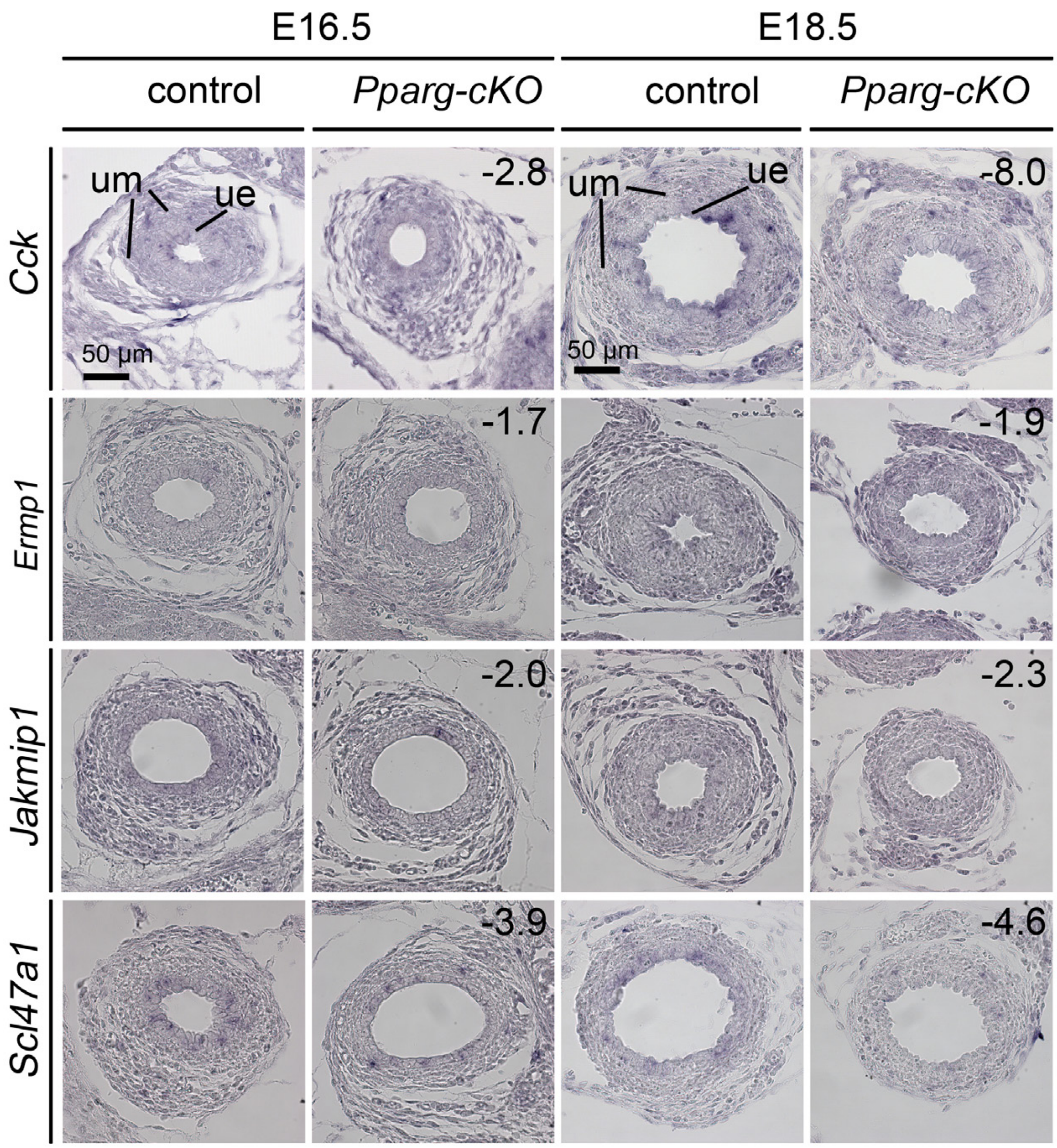

Fig. S9. RNA *in situ* hybridization analysis of genes with altered expression in microarrays of E16.5 and E18.5 *Pparg-cKO* ureters. Shown are RNA *in situ* hybridization analyses of transverse sections of the proximal ureter region of control and *Pparg-cKO* embryos at E16.5 and E18.5 for expression of genes, which were downregulated in *Pparg-cKO* microarrays. Numbers in the upper right corner relate to average fold change in the microarrays of E16.5 and E18.5 *Pparg-cKO* ureters. Probes, genotypes and fold change in the microarray are as indicated. $n \geq 3$ for each probe and genotype. ue, ureteric epithelium; um, ureteric mesenchyme.

Development • Supplementary information

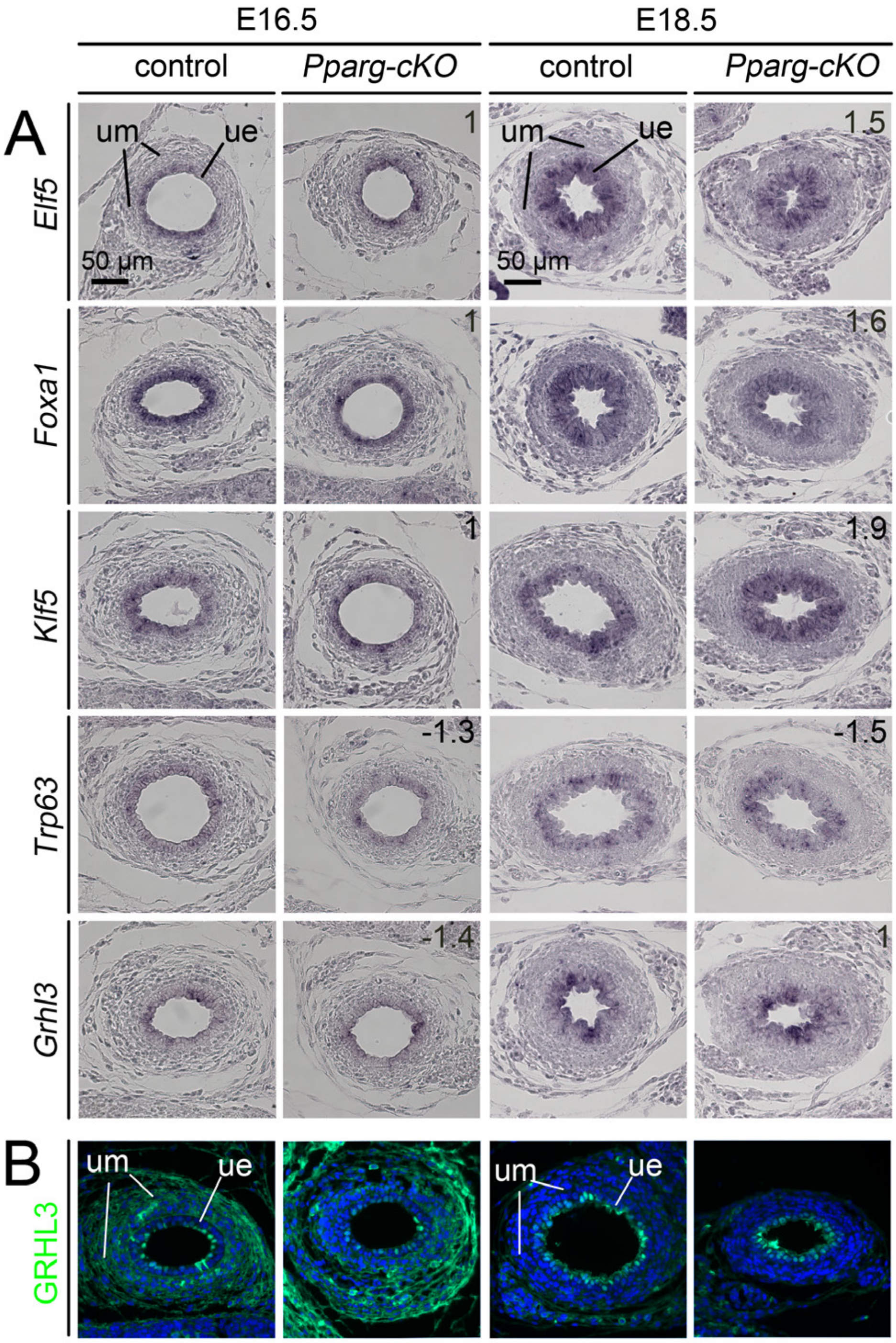

Fig. S10. Expression analysis of transcription factor genes/proteins in *Pparg-cKO* ureters at E16.5 and E18.5. (A,B) RNA *in situ* hybridization analysis for genes encoding transcription factors involved in urothelial development (A), and immunofluorescence of GRHL3 expression (B) on transverse sections of the proximal ureter region of control and *Pparg-cKO* embryos at E16.5 and E18.5. Numbers in the upper right corner (in A) relate to average fold change in the microarrays of E16.5 and E18.5 *Pparg-cKO* ureters. Probes and genotypes are as indicated. *n*≥3 for each probe and genotype. ue, ureteric epithelium; um, ureteric mesenchyme.

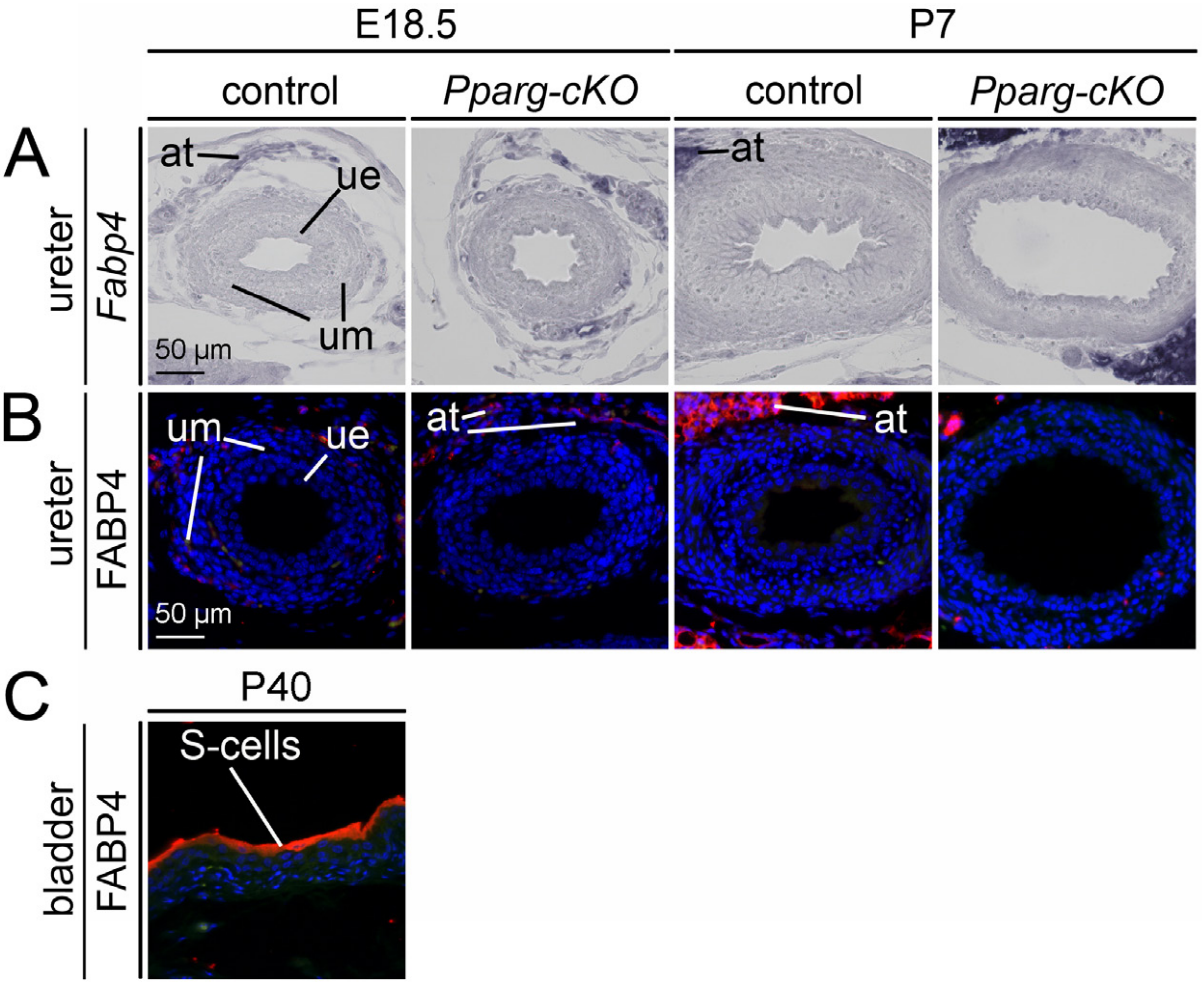

Fig. S11. Expression analysis of *Fabp4*/FABP4 in *Pparg-cKO* ureters. (A) RNA *in situ* hybridization analysis on transverse sections of the proximal ureter region of control and *Pparg-cKO* embryos at E18.5 and P7 for expression of *Fabp4*. Note that *Fabp4* expression is detected in adipose tissue surrounding the ureter but not in the urothelium. (B,C) Immunofluorescence analysis on transverse sections of the proximal ureter region of control and *Pparg-cKO* embryos at E18.5 and P7 (B) and on sagittal sections of the bladder of an adult P40 wildtype mouse (C) for expression of FABP4. Note that FABP4 expression is detected in adipose tissue surrounding the ureter and in S cells in the urothelium of the mature bladder but not in the urothelium of the ureter. Stages and genotypes are as indicated. $n \geq 3$ for each probe and genotype. at, adipose tissue; ue, ureteric epithelium; um, ureteric mesenchyme.

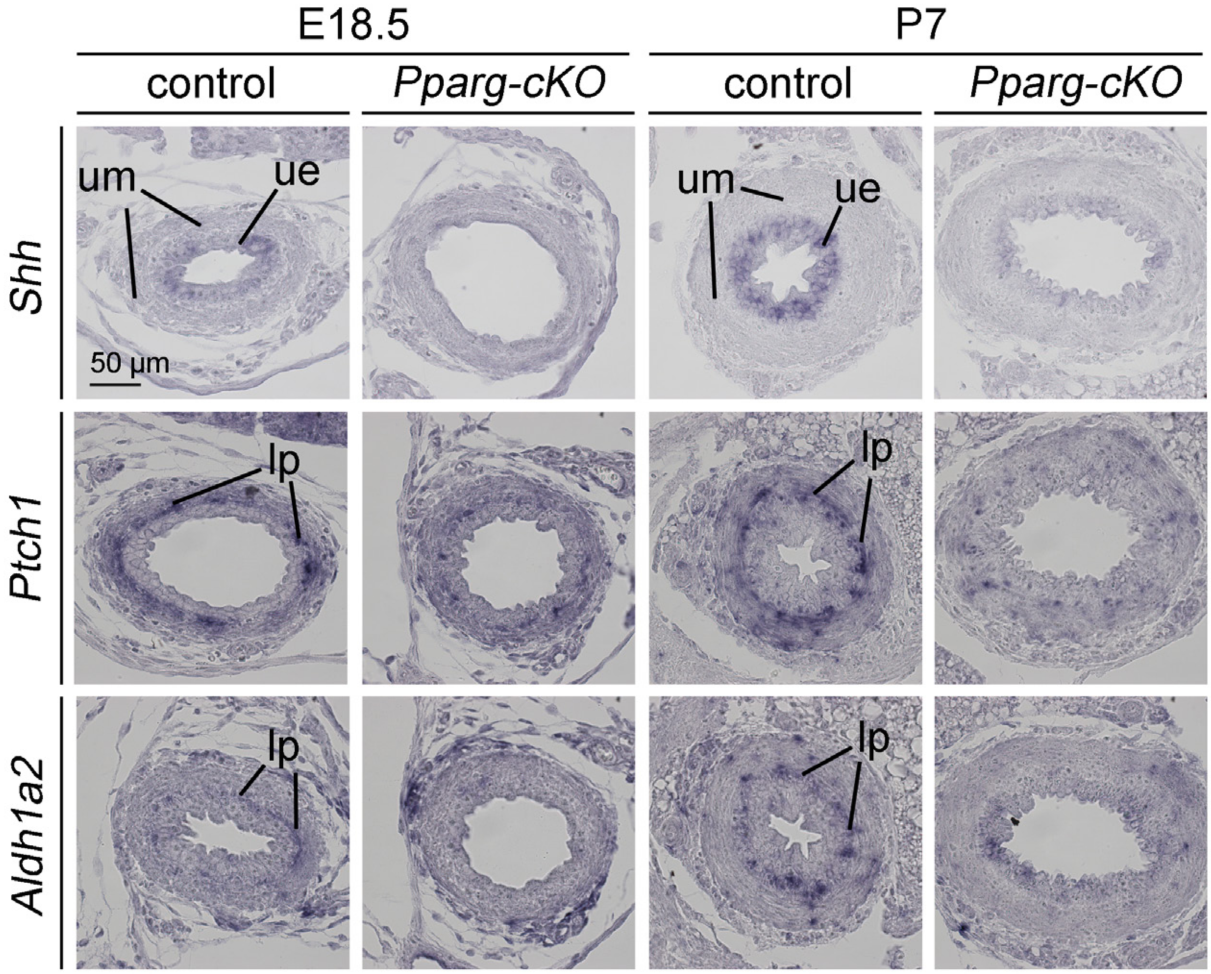

Fig. S12. SHH signaling is reduced in *Pparg-cKO* ureters. Shown are RNA *in situ* hybridization analyses of transverse sections of the proximal ureter region of control and *Pparg-cKO* mice at E18.5 and P7 for expression of *Shh*, *Ptch1* - a direct target of SHH signaling - and *Aldh1a2*. In the control, *Shh* is expressed in the urothelium whereas *Ptch1* and *Aldh1a2* are expressed in a patchy pattern in the peri-urothelial cells of the ureter, i.e. in the lamina propria. In *Pparg-cKO* ureters, *Shh* expression is lost, *Ptch1* is strongly reduced and *Aldh1a2* is not detectable in the lamina propria. Probes and genotypes are as indicated. $n \geq 3$ for each probe and genotype. lp, lamina propria; ue, ureteric epithelium; um, ureteric mesenchyme.

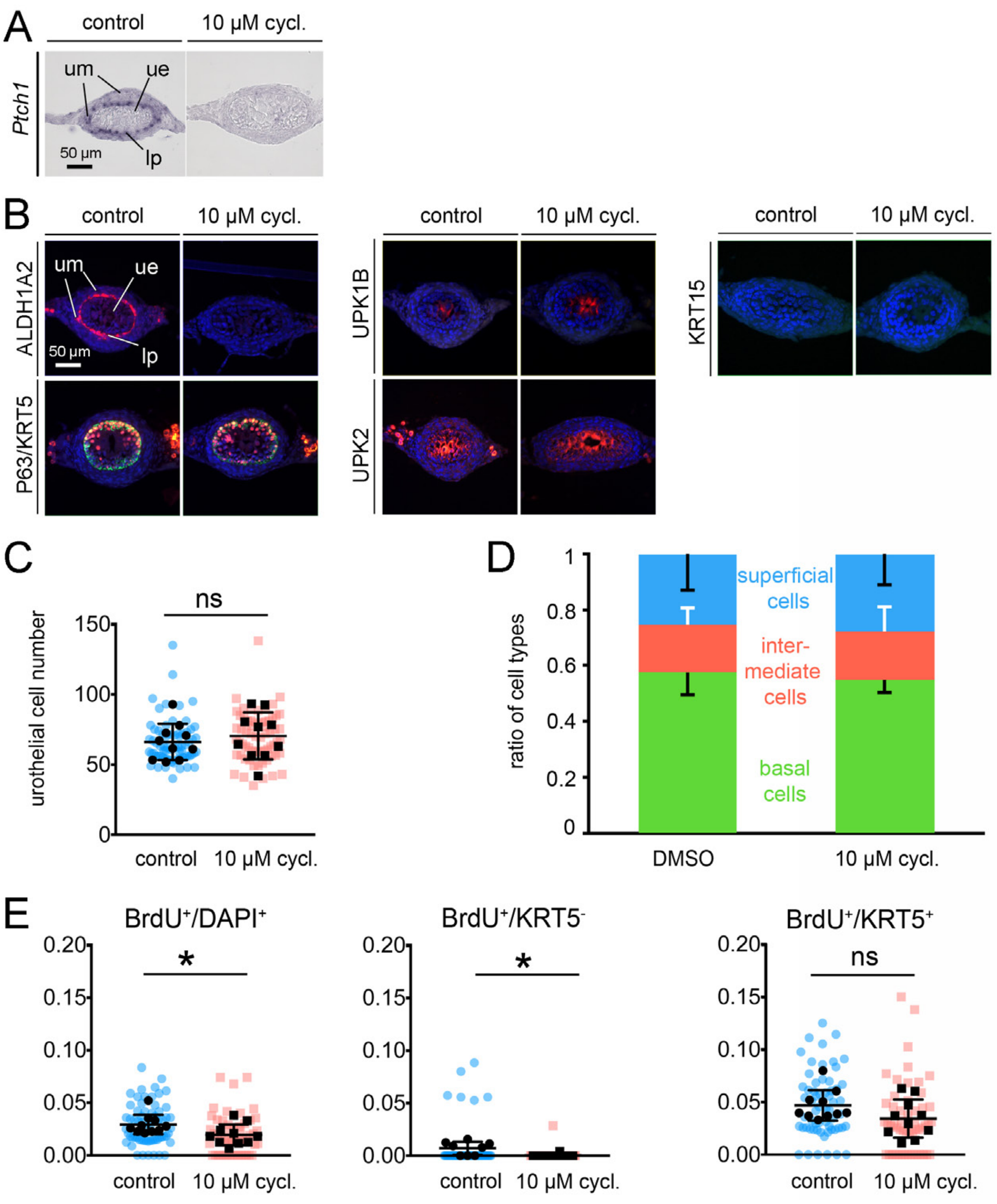
A
control
10 μM cycl.
Ptch1
um
ue
lp
50 μm
B
control
10 μM cycl.
ALDH1A2
P63/KRT5
UPK1B
UPK2
KRT15
C
ns
urothelial cell number
150
100
50
0
control
10 μM cycl.
D
ratio of cell types
1
0.8
0.6
0.4
0.2
0
superficial cells
inter-mediate cells
basal cells
DMSO
10 μM cycl.
E
BrdU+/DAPI+
BrdU+/KRT5-
BrdU+/KRT5+
*
ns
0.20
0.15
0.10
0.05
0.00

Fig. S13. Inhibition of SHH signaling in E18.5 ureter explant cultures does not affect urothelial development. (A) RNA *in situ* hybridization analysis on sections of proximal wildtype ureters explanted at E18.5 and cultured for 6 days in the absence or presence of 10 µM cyclopamine (cycl.) for expression of the target gene of SHH signaling, *Ptch1*. (B) Immunofluorescence analysis on sections of proximal wildtype ureters explanted at E18.5 and cultured for 6 days both in the absence and presence of 10 µM cyclopamine (cycl.) for markers of the lamina propria (ALDH1A2), B cells and I cells (P63), S cells (UPK1B, UPK2), and a regenerative response (KRT15). (C-E) Quantification of the overall urothelial cell number (C), of the ratio of luminal, intermediate and basal cells based on the immunofluorescence analysis for markers of B cells (KRT5), B cells and I cells (P63) and luminal cells (unstained) (D), and of proliferation by the BrdU assay in all urothelial cells ($BrdU^+/DAPI^+$), in non-basal/ luminal cells ($BrdU^+/KRT5^-$) and in basal cells ($BrdU^+/KRT5^+$) (E). Values are displayed as mean±sd. Two-tailed Student's t-test with Welch’s correction. ns, non-significant; **P*<0.05. Individual sections are presented as color-coded data points (blue dots for controls, red squares for 10 µM cyclopamine). For source data and statistics see Table S14.

Table S1. Genotype frequencies of animals derived from the breeding of *Pax2-cre/+;PParg*$^{fl/+}$ males and *Pparg*$^{fl/fl}$ *females* follows a Mendelian ratio.

Available for download at
https://journals.biologists.com/dev/article-lookup/doi/10.1242/dev.204324#supplementary-data

Table S2. Quantification of the circumference of the tubular lumen in control and *Pparg-cKO* ureters at E18.5, P7 and P40.

Available for download at
https://journals.biologists.com/dev/article-lookup/doi/10.1242/dev.204324#supplementary-data

Table S3. Quantification of the size of cells in the luminal cell layer in control and *Pparg-cKO* ureters at E18.5, P7 and P40.

Available for download at
https://journals.biologists.com/dev/article-lookup/doi/10.1242/dev.204324#supplementary-data

Table S4. Statistics on the peristaltic activity of explants of P0 control and *Pparg-cKO* ureters cultured for 6 days.

Available for download at
https://journals.biologists.com/dev/article-lookup/doi/10.1242/dev.204324#supplementary-data

Table S5. Cell proliferation analysis in the urothelium of control and *Pparg-cKO* ureters at E18.5 and P7.

Available for download at
https://journals.biologists.com/dev/article-lookup/doi/10.1242/dev.204324#supplementary-data

Table S6. Genes with increased expression in microarrays of *Pparg-cKO* ureters at E16.5.

Available for download at
https://journals.biologists.com/dev/article-lookup/doi/10.1242/dev.204324#supplementary-data

Table S7. Genes with decreased expression in microarrays of *Pparg-cKO* ureters at E16.5.

Available for download at
https://journals.biologists.com/dev/article-lookup/doi/10.1242/dev.204324#supplementary-data

Table S8. Genes with increased expression in microarrays of *Pparg-cKO* ureters at E18.5.

Available for download at
https://journals.biologists.com/dev/article-lookup/doi/10.1242/dev.204324#supplementary-data

Table S9. Genes with decreased expression in microarrays of *Pparg-cKO* ureters at E18.5.

Available for download at
https://journals.biologists.com/dev/article-lookup/doi/10.1242/dev.204324#supplementary-data

Table S10. Overlap of genes with increased expression in *Pparg-cKO* ureters at E16.5 and E18.5.

Available for download at
https://journals.biologists.com/dev/article-lookup/doi/10.1242/dev.204324#supplementary-data

Table S11. Overlap of genes with decreased expression in *Pparg-cKO* ureters at E16.5 and E18.5.

Available for download at
https://journals.biologists.com/dev/article-lookup/doi/10.1242/dev.204324#supplementary-data

Table S12. Functional annotations of genes with increased expression in microarrays of both E16.5 and E18.5 *Pparg-cKO* ureters.

Available for download at
https://journals.biologists.com/dev/article-lookup/doi/10.1242/dev.204324#supplementary-data

Table S13. Functional annotations of genes with decreased expression in microarrays of both E16.5 and E18.5 *Pparg-cKO* ureters.

Available for download at
https://journals.biologists.com/dev/article-lookup/doi/10.1242/dev.204324#supplementary-data

Table S14. Inhibition of SHH signaling in E18.5+6d ureter explant cultures does not affect urothelial development.

Available for download at
https://journals.biologists.com/dev/article-lookup/doi/10.1242/dev.204324#supplementary-data

Table S15. Restoration of SHH signaling partially rescues urothelial defects in explant cultures of E18.5+6d *Pparg-cKO* ureters.

Available for download at
https://journals.biologists.com/dev/article-lookup/doi/10.1242/dev.204324#supplementary-data

Table S16. Genes with increased expression in microarrays of P0 *Pparg-cKO* ureters cultured for 8 days.

Available for download at
https://journals.biologists.com/dev/article-lookup/doi/10.1242/dev.204324#supplementary-data

Table S17. Genes with decreased expression in microarrays of P0 *Pparg-cKO* ureters cultured for 8 days.

Available for download at
https://journals.biologists.com/dev/article-lookup/doi/10.1242/dev.204324#supplementary-data

Table S18. Functional annotations of genes with increased expression in microarrays of P0 *Pparg-cKO* ureters cultured for 8 days.

Available for download at
https://journals.biologists.com/dev/article-lookup/doi/10.1242/dev.204324#supplementary-data

Table S19. Functional annotations of genes with increased expression in microarrays of P0 *Pparg-cKO* ureters cultured for 8 days.

Available for download at
https://journals.biologists.com/dev/article-lookup/doi/10.1242/dev.204324#supplementary-data

Table S20. Genes with increased expression in microarrays of P0 *Pparg-cKO* ureters cultured for 8 days that show responsiveness to purmorphamine treatment.

Available for download at
https://journals.biologists.com/dev/article-lookup/doi/10.1242/dev.204324#supplementary-data

Table S21. Genes with decreased expression in microarrays of P0 *Pparg-cKO* ureters cultured for 8 days that show responsiveness to purmorphamine treatment.

Available for download at
https://journals.biologists.com/dev/article-lookup/doi/10.1242/dev.204324#supplementary-data

Table S22. Functional annotations of genes with increased expression in microarrays of P0 *Pparg-cKO* ureters cultured for 8 days that show responsiveness to purmorphamine treatment.

Available for download at
https://journals.biologists.com/dev/article-lookup/doi/10.1242/dev.204324#supplementary-data

Table S23. Functional annotations of genes with decreased expression in microarrays of P0 *Pparg-cKO* ureters cultured for 8 days that show responsiveness to purmorphamine treatment.

Available for download at
https://journals.biologists.com/dev/article-lookup/doi/10.1242/dev.204324#supplementary-data

Table S24. RT-qPCR analysis of gene expression.

Available for download at
https://journals.biologists.com/dev/article-lookup/doi/10.1242/dev.204324#supplementary-data

Table S25. List of primers used in this study.

Available for download at
https://journals.biologists.com/dev/article-lookup/doi/10.1242/dev.204324#supplementary-data

Table S26. List of primary and secondary antibodies used in this study.

Available for download at
https://journals.biologists.com/dev/article-lookup/doi/10.1242/dev.204324#supplementary-data
