# Supplementary material for: PPARG contributes to urothelial integrity in the murine ureter by activating the expression of Shh and superficial cell-specific genes
Source: Development. 2025 Apr 17;152(8):dev204324. doi: 10.1242/dev.204324 (PMC12045629; doi:10.1242/dev.204324)
Supplement: Supplementary file 1 [file develop-152-204324_review_history.pdf]

PPARG contributes to urothelial integrity in the murine ureter by activating the expression of Shh and of superficial cell-specific genes

Carsten Rudat, Philipp Straube, Jan Hegermann, Mark-Oliver Trowe, Hauke Thiesler, Herbert Hildebrand, Lisa Witt and Andreas Kispert
DOI: 10.1242/dev.204324

Editor: Liz Robertson

Review timeline
Original submission: 13 August 2024
Editorial decision: 1 October 2024
First revision received: 3 February 2025
Accepted: 5 March 2025

Original submission

First decision letter

MS ID#: dev.204324

MS TITLE: PPARG contributes to urothelial integrity in the murine ureter by activating the expression of Shh and of superficial-cell specific genes

AUTHORS: Carsten Rudat, Philipp Straube, Jan Hegermann, Mark-Oliver Trowe, Lisa Witt and Andreas Kispert

Dear Dr Kispert,

I have now received all the referees' reports on the above manuscript, and have reached a decision. The referees' comments are appended below, or you can access them online: please go to:

As you will see, the referees express considerable interest in your work, but have some significant criticisms and recommend a substantial revision of your manuscript before we can consider publication. If you are able to revise the manuscript along the lines suggested, which may involve further experiments, I will be happy receive a revised version of the manuscript. Your revised paper will be re-reviewed by one or more of the original referees, and acceptance of your manuscript will depend on your addressing satisfactorily the reviewers' major concerns. Please also note that Development will normally permit only one round of major revision. If it would be helpful, you are welcome to contact us to discuss your revision in greater detail. Please send us a point-by-point response indicating your plans for addressing the referees' comments, and we will look over this and provide further guidance.

Please attend to all of the reviewers' comments and ensure that you clearly highlight all changes made in the revised manuscript. Please avoid using 'Tracked changes' in Word files as these are lost in PDF conversion. I should be grateful if you would also provide a point-by-point response detailing how you have dealt with the points raised by the reviewers in the 'Response to Reviewers' box. If you do not agree with any of their criticisms or suggestions please explain clearly why this is so.

Reviewer 1

© 2025. Published by The Company of Biologists under the terms of the Creative Commons Attribution License (https://creativecommons.org/licenses/by/4.0/).

Reviewer 1: Summary of the Advance Made in This Paper and its Potential Significance to the Field:

The pathways that govern ureteral urothelium development and maintenance are incompletely understood. PPARG is known to regulate a program of superficial cell differentiation, but this pathway has not been thoroughly investigated in the ureteric epithelium. Here, Rudat et al. investigate the role of PPARG in the development and maintenance of ureteral urothelium. The authors used conditional genetic deletion of PPARG to functionally evaluate the role of PPARG in ureteral development/differentiation. Special stains, immunofluorescent localization, RNA in situ hybridization, and electron microscopy were used to evaluate ureteral expression and ureteral urothelium ultrastructure. Using microarray analyses the authors reveal that PPARG regulates a program of superficial cell structural gene expression and basal/intermediate cell Shh expression to cooperatively maintain ureteral urothelial integrity. Finally, using ureteral explants, Shh signaling was pharmacologically restored, and rescued/partially rescued the phenotypic defects in Pparg-cKO ureters. This lab has immense expertise in ureteral development, and the data presented are sound. This reviewer has offered the following critiques aimed at improving this manuscript:

Given that Weiss et al described 1) urothelial abnormalities (differentiation and basal cell proliferation) and hydroureter in Pparg-cKOs, and 2) Shh expression was impacted by Pparg loss, it seems the value added by this work is measured by Shh rescue in ureteral explants.

Suggestions to Authors:

Figure 1:
2nd sentence in section titled, "PPARG is differentially expressed in urothelial cells of the ureter." Consider clarifying the sentence that ends with "until" E18.5. It is unclear whether Pparg is expressed at elevated until E18.5, since the next timepoint evaluated was adult, and in the adult the figure legend states "Lack of Pparg detection at P40 likely reflects difficulty in preserving the mRNA at this (P40) stage." Furthermore, perhaps Pparg remains high through a yet to be defined neonatal period. Additionally, PPARG expression at P7 would be helpful in this figure, since this timepoint is important later in the manuscript.

Figure S1:
The text states "Using RNA in situ hybridization, we found increased expression of a Pparg transcript, which lacks the exon2/3 region (reference transcript: NM011146.3), in the UE of Pax2-cre/+;Ppargfl/fl (Pparg-cKO) embryos at E16.5 and E18.5." The figure suggests that unlike at E18.5, the E16.5 Pparg-cKO does not appear to have increased Pparg (3'UTR probe). Furthermore, the figure shows that the using the exon2/3 probe, there is a decrease Pparg in both cKOs. Clarification is required in the text, and quantification must be performed to claim the reported increase.

Figure 2:
How does increased tubular diameter differ from hydroureter, and what does "ureteral tissue was not flattened but partially folded" mean? What portion of the mice developed this tubular dilatation? Text states "strongly increased tubular diameter" - How was tubular diameter measured? How does this phenotype differ from the hydroureter in Hoxb7Cre;Pparg-cKO described by Weiss et al 2013?

Second to last sentence in section of section titled, "Loss of Pparg affects urothelial differentiation." The impact of Pparg deletion on SMC's and lamina propria is unclear. ALDH1A2 and ACATA2 (+TAGLN and CKM) are reportedly inhomogenous, and slightly decreased respectively. Consider quantifying using WB or RT-qPCR. (see Fig 6 comments for more details).

Last sentence of section titled, "Loss of Pparg affects urothelial differentiation." The data presented in Figure 2 alone does not lead to the conclusion that B-cells have undergone a regenerative response. Expression of Krt14 and Krt15 are associated with a regenerative response in bladder urothelium, as cited. Please clarify or save the "regenerative process/response" statement for the figures where regeneration is quantified.

Figure 3:

© 2025. Published by The Company of Biologists under the terms of the Creative Commons Attribution License (https://creativecommons.org/licenses/by/4.0/).

Would be helpful if there were specific references to Pparg-cKO compared to controls for more of the data in Figure 3 throughout the text for this figure. It was unclear at first whether I was looking at controls over time or looking at the difference in cKO compared to control over time in the text.

"Expression of KRT5 started at E16.5 in a few basal cells and labeled ALL cells of this layer from E18.5 as in the control." The KRT5 Pparg-cKO panel shows fewer KRT5+ basal cells than control, or at the least shows reduced KRT5 in basal cells at this time.

Images do not support the conclusion that "the expression of NP63 was not affected until E16.5."

KRT14 appears to be detected in most, but KRT15 does not appear to be detected in most. B-cells at P7 in Pparg-cKO. Instead, an abnormal presence of K14/K15 at this stage is a sufficient statement.

Conclusion that mutant urothelium engages in regenerative response at postnatal stages is inappropriate given increased expression of K14/K15 alone in Figure 3 (as I indicated for Fig 2 as well). Figure 4 gets at the regenerative response.

Figure S4:
Provide context for why the ultrastructural analysis was performed - ie explain that evidence of urothelial plaque is hallmark of urothelial differentiation in S cells. The arrowheads appear to be pointing at the "hinge" regions, while the plaques would be the intervening regions between the hinges in this scalloped structure. The importance for why the authors annotate mitochondria and comment on mitochondrial endowment being unaffected is unclear as it stands - clarify in the text. Furthermore, it might be helpful to annotate the fusiform/discoid vesicles and comment on their presence in control v cKO.

Figure 4:
Consider replacing TUNEL images from Panel A with Ki67 images. Consider adding TUNEL images to supplemental data. Otherwise Figure 4 could include both TUNEL images and Ki67 images.

Figure 6:
KRT15 is reported to be "unaltered in the mutant." KRT15 appears to be rescued in the image (reduced), yet the graph shows puromorphamine treated cKO have higher K15/DAPI ratio than the puromorphamine treated control.

Given the absence of an S cell marker (KRT20), and the patchy appearance of UPK1B and UPK2, it is difficult to appreciate how the puromorphamine treated Pparg-cKOs have more S-cells. This highlights the need to use IF for descriptive phenotyping and instead apply a different quantitative method. Greater than 20% S cell population in either mutant seems pretty high.

What does ACTA2 expression look like in this rescue? In my opinion, your Shh rescue presents an opportunity. This data in this paper and the figure in Weiss et al seem to reveal a decrease in ACTA2/SMA expression - which would make sense given the impact of Shh in Pparg-cKOs in both (Weiss et al shows an increase in SHH by IF [P1] while your microarray show Shh goes down [E16, E18]). Does purmorphamine not only rescue the urothelial phenotype but promote SMC fate/organization in these Pparg-cKOs? If you look at ACTA2 in this experiment, perhaps your analysis could explore IF-A expression intensity if you don't use RT-qPCR or WB. I realize the overarching theme of this manuscript is 'urothelial integrity', but given stromal-ALDH1A2 is included in this figure, it makes sense to also look at SMC-ACTA2 (as in Fig 2/3).

Restoration of Krt20+ S cells, or urothelial plaque by TEM?

Figure S10:
It is unclear why the authors conclude that "individual loss of SHH signaling in wildtype ureters does not affect urothelial DEVELOPMENT at POSTNATAL stages, when E18 ureteral explants are used for this assay. Instead, it would make more sense to conclude that SHH signaling does not affect urothelial 'maintenance' (since wild type ureters are being harvested at E18 and subjected to SHH signaling inhibition after a program of differentiation ensued). Otherwise, to address the role of

© 2025. Published by The Company of Biologists under the terms of the Creative Commons Attribution License (https://creativecommons.org/licenses/by/4.0/).

SHH in ureteral urothelium developmentally relevant context, ie would be important to harvest ureters for explants prior to the engagement of urothelial differentiation (E14/E16?), which is a timepoint of urothelial maturation that may also precede endogenous SHH signaling activity?!

Overall Critiques:
The definition of B, I and S cells is unclear. In the case of I v S cells, KRT20 expression would enable this important distinction. Clear definitions of the urothelial subsets is especially important given the quantification of the resultant urothelial phenotypes is based on microscopy and FIJI/ImageJ generated cell counts. FACS analyses was indicated in the Author Contributions, but not mentioned in the methods/results section. An unbiased FACS analysis would certainly strengthen the findings.

Similarly, small cells/cell size are mentioned a few times, but cell size is not quantified (or shown at higher magnification). While it is obvious that Pparg-cKO abrogates S cell formation, and S-cells are large, this makes perfect sense. However, given the absence of KRT20 marker, and the absence of large "umbrella cell" phenotype, it is difficult to appreciate how S cells were quantified here, especially since UPKs are expressed by I and S cells.

Would be helpful if there were specific references to figure panels for more of the results section throughout the body of the manuscript. Figure 1 is great, but Figure 2 and Figure 3 panel C covers a lot of diverse (ranging from urothelium 'superficial' and urothelium 'basal' to lamina propria and smooth muscle) data. There are many other sections where the absence of specific references to figure panels or cKO v control makes it difficult to find the right supporting data.

RT-qPCR/WB would strengthen this reviewer's confidence in the data analysis. While immunofluorescent localization provides necessary localization data, instances where the micrographs don't match the reported graphs or authors observations expose considerable criticisms. When possible, quantitative assays should be used.

The rationale for why the authors changed the timepoints for some of the microarray/explant validation or rescue experiments isn't stated.

The relationship between ureteral diameter and "tissue stiffness" at the end of the results section is unclear, especially as the authors point out that rescue of ureter diameter and tissue stiffness after mentioning enrichment of keratinization and cornification GO terms. This statement may be better suited for the discussion, and could then be further expanded given 1) the fact that defects in urothelial differentiation (UPK+ superficial cells) can result in loss of integrity/dilatation, and 2) the changes in stroma and smooth muscle reported in Pparg-cKOs may contribute to ureteral dilatation.

Prolonged proliferation doesn't fully explain hydroureter. Although in extreme cases, aberrant proliferation can cause luminal occlusion/obstruction and secondary hydroureter - this would also result in fairly significant hydronephrosis - which was not reported here. Instead consider discussing models with Upk-deficiencies that lead to urothelial plaque defects and loss of urothelial integrity that facilitate dilatation hydroureter/hydronephrosis (Carpenter et al 2016, Kong et al 2004, Hu et al 2000). These models not only lack Upk's, but also demonstrate compensatory basal cell regeneration (K14 expansion and proliferation, and in one case elevated SHH) which coincide with organ dilatation.

Does aberrant ureteral development/maturation functionally impact the ureter? Peristalsis/permeability? Could these (permeability) be evaluated in the rescue experiment? Otherwise, do the S-cells from the rescue experiment develop an apical urothelial plaque (TEM)?

Further discussion on GRHL3 as the alternative input for luminal differentiation program would be great.
1) Grhl3 is known to bind to the Upk2 promoter, but the discussion mentions Upk1b and Upk3b as being the Upks that are down-reglated but not absent in the Pparg-cKOs (not UPK2).
2) It would be interesting to see this discussion explore why Grhl3 is reduced in Pparg-deficient bladder urothelium (Liu et al 2019), but Grhl3 is not PPARg regulated in this model in ureteral urothelium, and GRHL3 was not explored.

© 2025. Published by The Company of Biologists under the terms of the Creative Commons Attribution License (https://creativecommons.org/licenses/by/4.0/).

<u>Reviewer 2</u>

Advance summary and potential significance to field

Overall: This report adds mechanistic detail to the existing knowledge that PPARG is involved in urothelial differentiation; specifically it shows that PPARG acts via shh in basal and intermediate cell types but probably by different paths in superficial cells. The work is done well and the figures are of high quality. The question will be of interest to a rather narrow readership, and as the authors correctly point out at the top of page 4, quite a lot is already known about PPARG in urothelial differentiation and the main advance here is to put shh in the pathway. Again, as the authors point out on page 4, the main interest will be to the cancer community (urothelial carcimonas). Therefore, while I think this paper is worth publishing (after minor corrections), I am not convinced that Development is the best place for it. This is not a comment on quality (I refuse to play those games - to me a paper is either worth publishing or is not, and 'rankings' of journals are irrelevant to my recommendations), but about core interests of the journal's readership. I would have thought the urology or cancer journal communities would be more interested and result in more citations and conference invitations and collaborations for the authors. But if the authors really want to reach a developmental community with this very specialized topic, fine.

Comments for the author

I liked the paper but I do recommend some minor changes:

Abstract: please define PPARG at its first use (ie give the full name).

Abstract: '2-layered urothelium' is ambiguous (can be read as meaning the whole thing is doubled): can you say 'urothelium with only two cell layers, not the usual....'?

Abstract: the sentence starting 'Moleculat analyses' would be clearer if it it were re-ordered to say 'Molecular analyses of fetal stages showed....'

Abstract: in the sentence about pharmacological manipulation, make clear that the reduction of ureter width is towards the normal width (not from normal to too-small).

(apologies if the above comments seem pedantic, but especially in the era of AI-assisted searches, it is really important that abstracts are not written in a way that can be read with the opposite of the intended meaning).

General: please standardize on whether you have spaces after the closing parenthesis in phrases like (S-)Cells {you sometimes have (S-) Cells and sometimes (S-)Cells}.

Intro paragraph 2: 'choreographed' seems a strange word (who is the choreographer meant to be?). Do you just mean 'reproducible' or 'standard' or 'stereotypic'? Also in this paragraph, I advise making clear that in the ureteric bud, 'distal' means furthest from the kidney: in lung etc., the convention is opposite, and this regularly causes confusion in readers who are not renal/urology specialists. Last paragraph page 3 - 'paramount' seems a very odd word but I cannot work out what word you really want; maybe just 'important'.

Results paragraph 1 - in Fig 1, as far as I can see, you did not look at transverse sections of embryos (as you write) but transverse sections of ureters. You need to add 'ureters of' ahead of 'mice'.

Legend to Fig 1 - why is mRNA preservation harder in the adult? Please could you explain this (in the results text accompanying Fig 1 rather than the legend, I suggest). I wonder if a quick qRTPCR is warranted just to check that the apparent fall of mRNA but not of protein really is an artefact of preservation, and not a real change in the relative stabilities of the mRNA and of the protein in adult life. If the latter is true, it would be a shame to have missed it in this paper! (I am not 'insisting' on this change as a reviewer, just recommending).

© 2025. Published by The Company of Biologists under the terms of the Creative Commons Attribution License (https://creativecommons.org/licenses/by/4.0/).

Results end of page 5 - I recommend you explain just a bit more about hydroureter for readers who are not urologists (at least, if you go ahead with this being in Development rather than a urological journal).

I like Fig 2D! - very useful.

Text describing Fig 4B (in the results section) - you use phrases like 'increased proliferation' in a study that involves a time-course. Please make clear that you mean increased in comparison with control, not increased in comparison with earlier times, which is a perfectly natural way of reading the word 'increased'. (assuming you do mean in comparison with controls - if you really do mean with respect to time as well, explain that carefully). This comment applies to several sentences in the paragraph.

'To unbiasedly identify' need to be be "To identify, in an unbiased manner, ". Also, in this paragraph, please justify the thresholds you used for microarray analysis. What happens if you use different ones? (Convince the reader you did not just fiddle about with thresholds until you saw something that you liked).

Final sentence of the paragraph on page 8 (before the next heading). Be careful about the claim - you don't know that PPARG controls the expression of these genes directly. All you know is that its presence/ absence affects whether or not they are expressed.

Discussion: I strongly advise deleting 'master' from the subheading; it binds you to a particular view of biology that is unnecessary and unhelpful, and gives critics a target at which to shoot. Just 'regulator' says all you need to say.

<u>Reviewer 3</u>

Advance summary and potential significance to field

The manuscript by Rudat et al. thoroughly examines the consequences of Pparg deletion in the mouse ureter. They show that Pparg regulates the formation/maintenance of the ureter in a Shh-dependent and a Shh-independent fashion. The experiments are well executed and clearly support the authors conclusions. There are just a few critiques that should be addressed before I can support publication in Development.

1. The authors should provide images for the KI67 staining (in particular in co-staining with Krt5) in the main figure. If they need space, they can move the images of the apoptosis to the Supplemental Material, as these don't show an effect.

2. The basal Krt15 expression in Fig. 6B is not really visible. Thus, it really does not support the claim of the authors. More convincing images need to be provided. Similarly, it is unclear, why the authors in their statistical analysis of the Krt15 data (Supplement al Table S12) used inconsistent statistical parameters (nonparametric vs. parametric normality, equal vs. unequal variance) and two-sided Mann-Whitney instead of the two-sided unpaired t-test used for all the other comparisons. Please expand on this.

3. The microarray analysis of the explanted ureters detected decreased expression of Keratins such as Krt6b, Krt16, Krt17, yet no validation of these data has been shown. The authors need to do so, to further support their data interpretation. The same should be done for some of the partially 'rescued' S-cell markers.

4. The authors published a couple of years ago the deletion of the Shh mediator Smoothened (Bohnenpoll et al., 2017). While the authors cite the paper, it is surprising that the authors never explicitly compare the results of the present study (which highlights the role of Pparg in regulating Shh expression), with the outcome of this study. Similarly, Bohnenpoll et al. (2017) identified Foxf1 as a major regulator for ureter development, but its expression is not examined in the present manuscript. This should be rectified.

© 2025. Published by The Company of Biologists under the terms of the Creative Commons Attribution License (https://creativecommons.org/licenses/by/4.0/).

5. The authors should also discuss in more detail why Inhibition of SHH signaling by cyclopamine in ureter explant cultures does not affect urothelial development similarly as in the Pparg-deficient mice, while hyperactivation actually rescues a much substantial aspect of the phenotype.

First revision

Author response to reviewers' comments

Response to Reviewers

Reviewer 1:

Summary of the Advance Made in This Paper and its Potential Significance to the Field
* The pathways that govern ureteral urothelium development and maintenance are incompletely understood. PPARG is known to regulate a program of superficial cell differentiation, but this pathway has not been thoroughly investigated in the ureteric epithelium. Here, Rudat et al. investigate the role of PPARG in the development and maintenance of ureteral urothelium. The authors used conditional genetic deletion of PPARG to functionally evaluate the role of PPARG in ureteral development/differentiation. Special stains, immunofluorescent localization, RNA in situ hybridization, and electron microscopy were used to evaluate ureteral expression and ureteral urothelium ultrastructure. Using microarray analyses the authors reveal that PPARG regulates a program of superficial cell structural gene expression and basal/intermediate cell Shh expression to cooperatively maintain ureteral urothelial integrity. Finally, using ureteral explants, Shh signaling was pharmacologically restored, and rescued/partially rescued the phenotypic defects in Pparg-cKO ureters. This lab has immense expertise in ureteral development, and the data presented are sound.
>> We very much thank the reviewer for his/her appreciation of our work. <<

* This reviewer has offered the following critiques aimed at improving this manuscript:
Given that Weiss et al described 1) urothelial abnormalities (differentiation and basal cell proliferation) and hydroureter in Pparg-cKOs, and 2) Shh expression was impacted by Pparg loss, it seems the value added by this work is measured by Shh rescue in ureteral explants.
>> Weiss et al provided an initial analysis of Pparg-mutant mice. They showed that classical S cell markers were reduced and that basal cells proliferated. However, there was no attempt to further characterize the cellular and molecular changes in the mutant at all. Weiss et al reported “hydroureter” in 10% of the mutants at different postnatal stages but did not analyze this abnormality - this leaves the possibility that the “hydroureter” is in fact not caused by increased hydrostatic pressure. Moreover, Weiss et al. provided very unclear statements on expression of Shh mRNA and SHH protein, which did not allow the conclusion that Shh expression is (completely) lost. Hence, we think that our analysis largely extends the initial descriptive work by Weiss et al., and provides detailed molecular analysis of control of S cell differentiation and the functional involvement of Shh in the phenotypic changes in the *Pparg*-mutant ureter, and hence, in urothelial development. <<

Suggestions to Authors:
* Figure 1:
2nd sentence in section titled, "PPARG is differentially expressed in urothelial cells of the ureter." Consider clarifying the sentence that ends with "until" E18.5. It is unclear whether Pparg is expressed at elevated until E18.5, since the next timepoint evaluated was adult, and in the adult the figure legend states "Lack of Pparg detection at P40 likely reflects difficulty in preserving the mRNA at this (P40) stage." Furthermore, perhaps Pparg remains high through a yet to be defined neonatal period. Additionally, PPARG expression at P7 would be helpful in this figure, since this timepoint is important later in the manuscript.
>> We agree with the reviewer that the Pparg/PPARG expression analysis would benefit from

© 2025. Published by The Company of Biologists under the terms of the Creative Commons Attribution License (https://creativecommons.org/licenses/by/4.0/).

an extended analysis of ureters at postnatal stages. Therefore, we have added P0, P7 and P14 ureters to better cover the stages until P40. Moreover, we have optimized the fixation protocol for postnatal ureters to preserve *Pparg* mRNA integrity. We now show that *Pparg* mRNA is strongly expressed in the urothelium from E16.5 to P40, and that PPARG protein levels are higher in luminal (S) cells at these stages. <<

*Figure S1:
The text states "Using RNA in situ hybridization, we found increased expression of a Pparg transcript, which lacks the exon2/3 region (reference transcript: NM011146.3), in the UE of Pax2-cre/+;Ppargfl/fl (Pparg-cKO) embryos at E16.5 and E18.5." The figure suggests that unlike at E18.5, the E16.5 Pparg-cKO does not appear to have increased Pparg (3'UTR probe). Furthermore, the figure shows that the using the exon2/3 probe, there is a decrease Pparg in both cKOs. Clarification is required in the text, and quantification must be performed to claim the reported increase.
>> The reviewer is right that the text erroneously stated that the 3' UTR probe detects increased expression of a Pparg transcript at E16.5. Moreover, we did not mention the result of the 3' UTR probe in the main text (but only in the legend to Figure S1). This might have caused confusion. To clarify the *Pparg* expression analysis in the mutant setting, we describe our findings in an extended and thus, hopefully, in a more comprehensive manner in the revised text. We now write: "Using the exon2/3 region and the 3'-UTR of a *Pparg* cDNA (reference transcript: NM011146.3) as probes in RNA *in situ* hybridization analysis, we detected in the UE of E16.5 and E18.5 *Pax2-cre/+;Pparg$^{fl/fl}$ (Pparg-cKO)* embryos, expression of a *Pparg* mRNA lacking the exon2/3 region. At E18.5, the expression of the mutant transcript was increased compared to the control, indicating a compensatory upregulation (Fig. S1A). Importantly, immunohistochemical analysis using a monoclonal antibody raised against the N-terminal PPARG region detected a protein at E16.5 and E18.5 in the control but not in the mutant UE (Fig. S1B). This is consistent with the prediction that cre-mediated deletion of exon2/3 from the floxed *Pparg* allele results in a truncated protein lacking the N-terminal region, including parts of the DNA- binding domain, and thus a non-functional protein (He et al., 2003)."
We further elaborated this in the legend to Figure S1.
The statement that the *Pparg* transcript - as detected by the 3-UTR probe - is increased in *Pparg-cKO* ureters at E18.5 is supported by the microarray analysis of *Pparg-cKO* ureters at this stage. Together with the fact, that the floxed allele of *Pparg*, which we used in our experiments, has been used and described many times before, we deem that RT-PCR would not provide additional insight but lead to an unnecessary consumption of animals. <<

* Figure 2:
How does increased tubular diameter differ from hydroureter, and what does "ureteral tissue was not flattened but partially folded" mean? What portion of the mice developed this tubular dilatation? Text states "strongly increased tubular diameter" - How was tubular diameter measured? How does this phenotype differ from the hydroureter in Hoxb7Cre;Pparg-cKO described by Weiss et al 2013?
>> We have rephrased our wording to provide (hopefully) a better description of the histology of Pparg-cKO ureters at P40. Moreover, we have determined the luminal circumference on transverse sections to provide a quantification for the statement that the tubular lumen is increased (new Figure S3, Table S2). The text now reads: "However, histological analysis of *Pparg-cKO* ureters at P40 revealed a greatly enlarged tubular lumen with an irregular outline (Fig. 2A, Fig. S3, Table S2)."
Weiss et al. mentioned hydroureter formation in approximately 10% of their mutant animals. However, they reported that the other 90% of the adult animals had a ureter with a starfish pattern of the urothelium. Looking at their images (Weiss et al., 2014; Figures S4 and S5), it seems likely that the term "starfish" described the same phenotypic change, that we also observed, namely the increased ureteral lumen with an irregular course of the urothelium.
Please note that we removed the sentence on hydroureter from the result section. We think that a comparison to the Weiss study and hydroureter formation better fits to the discussion. We therefore have added a small section at the end of the discussion on the morphological changes in the Pparg-cKO ureter, and their possible cause. <<

© 2025. Published by The Company of Biologists under the terms of the Creative Commons Attribution License (https://creativecommons.org/licenses/by/4.0/).

* Second to last sentence in section of section titled, "Loss of Pparg affects urothelial differentiation." The impact of Pparg deletion on SMC's and lamina propria is unclear. ALDH1A2 and ACATA2 (+TAGLN and CKM) are reportedly inhomogenous, and slightly decreased respectively. Consider quantifying using WB or RT-qPCR. (see Fig 6 comments for more details).
>> Pparg-cKO ureters have a greatly enlarged tubular lumen. We think that the weaker expression of SMC markers at this stage is secondary to luminal widening and possibly tissue stretching. The expression of ALDH1A2 is rather normal, we deleted the statement that the expression in the mutant is changed.
Given the large increase of the tubular lumen and urothelial circumference in *Pparg- cKO* ureters, it doesn't really make sense to quantify SMC and LP differentiation. Please note that at early stages (until P7) SMCs markers are normal. Moreover, we have performed peristalsis assays with cultured explants of P0 ureters, and found normal peristaltic activity (new Fig. S6). This confirms that SMCs changes observed at P40 do not reflect a primary requirement of Pparg in this tissue, but are secondary in nature. In contrast, Aldh1a2 expression starts delayed which relates to reduced SHH signaling as discussed in the text. <<

* Last sentence of section titled, "Loss of Pparg affects urothelial differentiation." The data presented in Figure 2 alone does not lead to the conclusion that B-cells have undergone a regenerative response. Expression of Krt14 and Krt15 are associated with a regenerative response in bladder urothelium, as cited. Please clarify or save the "regenerative process/response" statement for the figures where regeneration is quantified.
>> Thanks for this suggestion. We have changed the last sentence of this section to "In conclusion, P40 *Pparg-cKO* ureters have a 2-layered urothelium with undifferentiated luminal cells and differentiated B cells. The latter ectopically express KRT14 and KRT15 (Fig. 2D, schematic). The SMC layer also appears to be affected, possibly related to the dilation of the ureteral lumen.“ <<

*Figure 3:
Would be helpful if there were specific references to Pparg-cKO compared to controls for more of the data in Figure 3 throughout the text for this figure. It was unclear at first whether I was looking at controls over time or looking at the difference in cKO compared to control over time in the text.
>> Sorry for poor referencing. We have added numerous references to Pparg-cKO and control mice both in the text relating to "onset and progression of urothelial defects" and in all other sections in the revised version of our manuscript. <<

* "Expression of KRT5 started at E16.5 in a few basal cells and labeled ALL cells of this layer from E18.5 as in the control." The KRT5 Pparg-cKO panel shows fewer KRT5+ basal cells than control, or at the least shows reduced KRT5 in basal cells at this time.
>> To support our statement that KRT5 expression is unaffected in the mutant urothelium at E18.5, we have replaced the old KRT5 Pparg-cKO image for a more representative one in Fig. 3C and write in text:
"KRT5 expression was not affected in the urothelium of the *Pparg-cKO* ureter: expression started in a few basal cells at E16.5, labeled about half of the cells in this layer at E18.5, and was found in all B cells at P7 as in the control (Fig. 3C)."

* Images do not support the conclusion that "the expression of NP63 was not affected until E16.5."
>> We respectfully disagree with the reviewer that expression of ΔNP63 is affected at E14.5. In fact, ΔNP63 expression is activated normally at E14.5 in the *Pparg-cKO* ureter, and the number of ΔNP63+ cells is unchanged compared to the control. <<

* KRT14 appears to be detected in most, but KRT15 does not appear to be detected in most. B-cells at P7 in Pparg-cKO. Instead, an abnormal presence of K14/K15 at this stage is a sufficient statement.
>> To accommodate this concern of the reviewer, we now write in the revised version: "Expression of both KRT14 and KRT15 was ectopically activated in B cells of the *Pparg-cKO* ureter at P7." <<

© 2025. Published by The Company of Biologists under the terms of the Creative Commons Attribution License (https://creativecommons.org/licenses/by/4.0/).

* Conclusion that mutant urothelium engages in regenerative response at postnatal stages is inappropriate given increased expression of K14/K15 alone in Figure 3 (as I indicated for Fig 2 as well). Figure 4 gets at the regenerative response.
>> We have changed our conclusion. It now reads: "We conclude that in the *Pparg-cKO* ureter, the urothelium fails to activate S-cell differentiation at E16.5 and generate I-cells for further stratification from E18.5, but ectopically expresses KRT14 and KRT15 at postnatal stages (Fig. 3D, schematic)." <<

* Figure S4:
Provide context for why the ultrastructural analysis was performed - ie explain that evidence of urothelial plaque is hallmark of urothelial differentiation in S cells. The arrowheads appear to be pointing at the "hinge" regions, while the plaques would be the intervening regions between the hinges in this scalloped structure. The importance for why the authors annotate mitochondria and comment on mitochondrial endowment being unaffected is unclear as it stands - clarify in the text. Furthermore, it might be helpful to annotate the fusiform/discoid vesicles and comment on their presence in control v cKO.
>> We have rephrased the section on the ultrastructural analysis to accommodate the concern of the reviewer. It now reads:
"Given a previous report that loss of *Pparg* in the bladder urothelium affects mitochondrial structure in the luminal layer, we performed an ultrastructural analysis at E18.5. In the *Pparg-cKO* ureter, the mitochondrial endowment appeared unaffected. However, consistent with impaired S-cell differentiation, hinges, known to separate urothelial plaques, were absent from the surface, which was instead covered with a large number of small vesicular bodies. In addition, fusiform vesicles, that are a compartment for intracellular transport of urothelial plaques, were smaller and oval-shaped in the *Pparg-cKO* (Fig. S7). <<

* Figure 4:
Consider replacing TUNEL images from Panel A with Ki67 images. Consider adding TUNEL images to supplemental data. Otherwise Figure 4 could include both TUNEL images and Ki67 images.
>> New Figure 4 now includes both TUNEL and Ki67/KRT5 images. <<

* Figure 6:
KRT15 is reported to be "unaltered in the mutant." KRT15 appears to be rescued in the image (reduced), yet the graph shows puromorphamine treated cKO have higher K15/DAPI ratio than the puromorphamine treated control.
>> Since the KRT15 staining is relatively weak in cultures ureters, we have added arrowheads in Figure 6B to mark the KRT15$^+$ cells in the urothelium. There are seven cells labeled in the untreated mutant, and four cells labeled in the purmorphamine- treated mutant ureter, suggesting that there is a "rescue" of KRT15 ectopia. The quantification tells that the ratio of KRT15 to DAPI$^+$ cells is indeed unchanged or slightly increased. While this seems counter-intuitive at first glance, it is easily
explained by the fact that there are many more cells in the untreated mutant urothelium than in the treated one. <<

* Given the absence of an S cell marker (KRT20), and the patchy appearance of UPK1B and UPK2, it is difficult to appreciate how the puromorphamine treated Pparg-cKOs have more S-cells. This highlights the need to use IF for descriptive phenotyping and instead apply a different quantitative method. Greater than 20% S cell population in either mutant seems pretty high.
>> The images for UPK1B and UPK2 staining show that S-cell differentiation is compromised in the *Pparg-cKO* mutant (the few residual cells may relate to non-recombined precursors). S-cell differentiation is not rescued by purmorphamine treatment.
In (D) we quantified the number of cells in the different layers to determine whether I cells were rescued after purmorphamine treatment in the mutant urothelium. For this, we did not count "S-cells" using a differentiation marker such as UPK or KRT20 since we have shown that differentiated S cells are largely absent in the mutant. Instead,

© 2025. Published by The Company of Biologists under the terms of the Creative Commons Attribution License (https://creativecommons.org/licenses/by/4.0/).

we used localization and lack of KRT5 and P63 expression to count the number of luminal cells. Accordingly, intermediate cells were quantified as $KRT5^{-}P63^{+}$ cells, and basal cells as $KRT5^{+}P63^{+}$ cells.
Please note that we have replaced the term superficial cell by luminal cell in Figure 6D, and changes the wording in the figure legend to describe this assay.
Hence, the mutant urothelium does not have 20% differentiated S cells, but 25-30% of all urothelial cells are luminal cells. <<

* What does ACTA2 expression look like in this rescue? In my opinion, your Shh rescue presents an opportunity. This data in this paper and the figure in Weiss et al seem to reveal a decrease in ACTA2/SMA expression - which would make sense given the impact of Shh in Pparg-cKOs in both (Weiss et al shows an increase in SHH by IF [P1] while your microarray show Shh goes down [E16, E18]). Does purmorphamine not only rescue the urothelial phenotype but promote SMC fate/organization in these Pparg-cKOs? If you look at ACTA2 in this experiment, perhaps your analysis could explore IF-A expression intensity if you don't use RT- qPCR or WB. I realize the overarching theme of this manuscript is 'urothelial integrity', but given stromal-ALDH1A2 is included in this figure, it makes sense to also look at SMC-ACTA2 (as in Fig 2/3).

>> Our analysis showed that expression of SMC markers is reduced in the Pparg- cKO ureter at P40. Our analysis of earlier stages (E16.5 to P7) showed normal SMC differentiation. To ensure that peristaltic activity is not compromised in the mutant, we determined the peristaltic activity of P0 ureter explants cultured for 6 days. We observed normal onset, frequency and intensity of contractions in *Pparg-cKO* ureters (new Figure S6). This shows that epithelial loss of *Pparg* does not impact on
structural components of the peristaltic machinery (SMCs and pacemaker cells) and their physiology at fetal and early postnatal stages. We therefore think that reduced SMC investment in in P40 *Pparg-cKO* ureters is secondary to ureteral dilatation at this stage. In fact, it is well known that dilatation causes dedifferentiation of SMCs, see UUO models!
Since SMC differentiation and peristaltic activity is not affected in the *Pparg-cKO* ureter at fetal and early postnatal stages, it does not make sense to look at changes of SMCs after purmorphamine treatment.
We would like to stress that Shh signaling plays different roles in ureter development. From E12.5 to E14.5 it is required for growth and to switch on the differentiation programs of the ureter. After E18.5/P0 it is required to induce lamina propria development (please refer to our latest paper (Development 2025, dev.204214), but is not uniquely required for the maintenance of SMCs. Indirectly, as we show in this paper, SHH signaling also impinges on the maintenance/regeneration of the urothelial cell types.

* Restoration of Krt20+ S cells, or urothelial plaque by TEM?
>> We have shown that S-cell differentiation is not restored in the mutant urothelium by purmorhamine treatment to a level that we would see large luminal cells positive for UPKs (Figure 6B). Our profiling experiments show that expression of S cell differentiation genes is upregulated (unexpectedly) but this does not lead to formation of differentiated S cells. We therefore think that TEM would not provide more insight. <<

* Figure S10:
It is unclear why the authors conclude that "individual loss of SHH signaling in wildtype ureters does not affect urothelial DEVELOPMENT at POSTNATAL stages, when E18 ureteral explants are used for this assay. Instead, it would make more sense to conclude that SHH signaling does not affect urothelial 'maintenance' (since wild type ureters are being harvested at E18 and subjected to SHH signaling inhibition after a program of differentiation ensued). Otherwise, to address the role of SHH in ureteral urothelium developmentally relevant context, ie would be important to harvest ureters for explants prior to the engagement of urothelial differentiation (E14/E16?), which is a timepoint of urothelial maturation that may also precede endogenous SHH signaling activity?!
>> To avoid confusion, we have changed this sentence to:
“This indicates that the individual loss of SHH signaling in wildtype ureters does not affect urothelial development and/or maintenance at late fetal and early postnatal stages”.

© 2025. Published by The Company of Biologists under the terms of the Creative Commons Attribution License (https://creativecommons.org/licenses/by/4.0/).

We would like to point out, though, that urothelial and ureter development does not finish at E18.5. In fact, I cells expand after birth and S-cells increase in size. <<

* Overall Critiques:
The definition of B, I and S cells is unclear. In the case of I v S cells, KRT20 expression would enable this important distinction. Clear definitions of the urothelial subsets is especially important given the quantification of the resultant urothelial phenotypes is based on microscopy and FIJI/ImageJ generated cell counts.
>> We had pointed out in the text for Figure 2 that urothelial cell types in the mouse ureter can be discriminated by the combinatorial expression of KRT5, P63 and UPKs. Since this might have been unclear, we now added a section in the introduction stating this more explicitly. It reads:
"In addition to shape and location, urothelial cells can be molecularly distinguished by the combinatorial expression of cytokeratin 5 (KRT5), an isoform of the transcription factor p63 (ΔNP63) and UPKs. B cells are KRT5^{+}ΔNP63^{+}UPK1B^{-}, I cells are KRT5^{-} ΔNP63^{+}UPK1B^{+}, and S cells are KRT5^{-}ΔNP63^{-}UPK1B^{+}, with levels of UPK1B being significantly lower in I cells than in S cells (Bohnenpoll et al., 2017a; Gandhi et al., 2013)."
We would like to point out that KRT20 is a great marker for S cells in the bladder. In the mouse ureter, KRT20 only stains adult S cells reliably.
We again like to stress that cell counts for differentiated S cells were not done in this study. We counted luminal cells by their localization and absence of P63 and Krt5 expression. <<

* FACS analyses was indicated in the Author Contributions, but not mentioned in the methods/results section. An unbiased FACS analysis would certainly strengthen the findings.
>> Sorry, mentioning of FACS in the author contribution was an error. We have removed it. Moreover, there are no cell surface markers available, which would allow FACS analysis of urothelial cells in the ureter. <<

*. Similarly, small cells/cell size are mentioned a few times, but cell size is not quantified (or shown at higher magnification). While it is obvious that Pparg-cKO abrogates S cell formation, and S-cells are large, this makes perfect sense. However, given the absence of KRT20 marker, and the absence of large "umbrella cell" phenotype, it is difficult to appreciate how S cells were quantified here, especially since UPKs are expressed by I and S cells.
>> In the revised version of our manuscript, we have quantified the size of luminal cells in control and *Pparg-cKO* ureters by measuring the area of these cells on transverse sections stained with CDH1. The data confirm the impression that the size of luminal cells is significantly reduced at E18.5, P7 and P40 (see new Figure S4, Table S3). We would like to stress that we measured cells of the luminal layer, which are easy to detect by location and CDH1 expression. Due to the obvious lack of S cell markers in the mutant urothelium, we do not designate them as S cells but as luminal cells. <<

* Would be helpful if there were specific references to figure panels for more of the results section throughout the body of the manuscript. Figure 1 is great, but Figure 2 and Figure 3 panel C covers a lot of diverse (ranging from urothelium 'superficial' and urothelium 'basal' to lamina propria and smooth muscle) data. There are many other sections where the absence of specific references to figure panels or cKO v control makes it difficult to find the right supporting data.
>> We have added a lot more references to the genotypes and the figures in the revised version of the manuscript. We very much hope that this improves the readability. <<

* RT-qPCR/WB would strengthen this reviewer's confidence in the data analysis. While immunofluorescent localization provides necessary localization data, instances where the micrographs don't match the reported graphs or authors observations expose considerable criticisms. When possible, quantitative assays should be used.
>> We are very disappointed that the reviewer does not fully trust our data analysis. We have taken exquisite care that all our assays are performed with technical expertise and optimization, that the relevant number of independent specimens were analyzed, that the data were quantified when possible and interpreted in a careful manner. The size of our

© 2025. Published by The Company of Biologists under the terms of the Creative Commons Attribution License (https://creativecommons.org/licenses/by/4.0/).

supplemental data gives testimony to this.
If in one or two circumstances the images were not carefully chosen to be representative, we apologize. We have changed them.
We have newly added quantifications for the ureteral lumen, the size of luminal cells and the peristaltic activity of *Pparg-cKO* ureters as requested from the reviewer(s). Moreover, we have performed RT-qPCR analysis to confirm the findings from the transcriptional profiling of the rescue experiments.
As mentioned above, it doesn't make sense to quantify protein expression by WB when the mutant ureter is dilated (Figure 2). The immunofluorescence analysis of onset and progression of the defects (analysis of E14.5 to P7) (Figure 3) in mandatory because we do not want to know overall expression changes but cell-type specific changes. We have validated the microarray profiling (a quantitative assay!!) with RNA in situs since we want information on cell type specificity (Figure 5).
Finally, I would also like to indicate that animal experiments should obey the 3R rule. One should not perform experiments that have a very high animal consumption but provide only incremental gain of knowledge. <<

* The rationale for why the authors changed the timepoints for some of the microarray/explant validation or rescue experiments isn't stated.
>> During the course of the experimental work for this manuscript, the legislation for animal protection changed in Germany and Lower Saxony, respectively. While isolation of fetuses both of wildtype and mutant mice was possible without permission before, it is now required to apply for permission with the authorities. Since these applications are lengthy, and the work of the students should not be jeopardized, we decided to use P0 instead E18.5 ureters for some experiments.
Since the two stages are close together, we assume that the experimental results are unaffected. We now mention this fact in the "Results" section… <<

* The relationship between ureteral diameter and "tissue stiffness" at the end of the results section is unclear, especially as the authors point out that rescue of ureter diameter and tissue stiffness after mentioning enrichment of keratinization and cornification GO terms. This statement may be better suited for the discussion, and could then be further expanded given 1) the fact that defects in urothelial differentiation (UPK+ superficial cells) can result in loss of integrity/dilatation, and 2) the changes in stroma and smooth muscle reported in Pparg-cKOs may contribute to ureteral dilatation.
>> Thanks for this advise. We have deleted the sentence "This may explain the rescue of the ureteral diameter and thus tissue stiffness of *Pparg-cKO* ureters by purmorphamine treatment" from the result section in the revised version of our manuscript. Instead, we discuss these findings with respect to ureteral dilatation/hydroureter formation in the new (last) section in the discussion. <<

* Text
Prolonged proliferation doesn't fully explain hydroureter. Although in extreme cases, aberrant proliferation can cause luminal occlusion/obstruction and secondary hydroureter - this would also result in fairly significant hydronephrosis which was not reported here. Instead consider discussing models with Upk-deficiencies that lead to urothelial plaque defects and loss of urothelial integrity that facilitate dilatation hydroureter/hydronephrosis (Carpenter et al 2016, Kong et al 2004, Hu et al 2000). These models not only lack Upk's, but also demonstrate compensatory basal cell regeneration (K14 expansion and proliferation, and in one case elevated SHH) which coincide with organ dilatation.
>> Thanks for this important advice. To avoid confusion, we decided to omit the term hydroureter in the result section related to Figure 2. Instead, as suggested by the reviewer, we have added a new section to the end of the discussion, where we discuss ureteral dilatation (Hydroureter) in *Pparg-cKo* ureter, its late temporal occurrence and its possible causes, also in the light of the finings in the Upk mutants. It reads: "Similar to a previously reported conditional knockout model, *Pparg-cKO* mice developed progressive bilateral ureteral dilatation with parenchymal thinning by P40 (Weiss et al., 2013). The hydroureter, as this condition is known, was not caused by SMC or pacemaker defects, as the expression of SMC markers was normal and peristalsis was unaffected in the perinatal stages. We also found no evidence of physical obstruction along the ureter and the vesicoureteric junction as a

© 2025. Published by The Company of Biologists under the terms of the Creative Commons Attribution License (https://creativecommons.org/licenses/by/4.0/).

possible cause.
In “classical” hydroureter, the accumulation of urine leads to a uniform expansion of the luminal circumference and a tissue thinning. In *Pparg-cKO* ureters, the luminal circumference was uneven and folded, arguing against a fluid-mediated dilatation. Since the expression of numerous keratins and basal cell proliferation was greatly increased postnatally, it is possible that proliferative urothelial expansion is a cause and not a consequence of the luminal dilatation. However, lack of differentiated S cells may contribute to dilatation by disrupting urothelial integrity, widening of the ureteral orifice and causing VUR, as observed in mice lacking *Upk*s (Carpenter et al., 2016; Hu et al., 2000; Kong et al., 2004)”. <<

* Does aberrant ureteral development/maturation functionally impact the ureter? Peristalsis/permeability? Could these (permeability) be evaluated in the rescue experiment? Otherwise, do the S-cells from the rescue experiment develop an apical urothelial plaque (TEM)?
>> Our marker analysis (Fig. 3C) showed that SMC markers are normally expressed in the Pparg-cKO ureter at E16.5 to P7. To ensure that peristaltic activity is not compromised in the mutant, we determined the peristaltic activity of P0 ureter explants cultured for 6 days. We observed normal onset, frequency and intensity of contractions in *Pparg-cKO* ureters (new Figure S6). This shows that epithelial loss of *Pparg* does not impact on structural components of the peristaltic machinery (SMCs and pacemaker cells) and their physiology at fetal and early postnatal stages. We therefore think that reduced SMC investment in P40 Pparg-cKO ureters is secondary to ureteral dilatation at this stage.
We did not perform further analysis of the structural integrity of the mutant ureter (including permeability assays and TEM), since our study was aimed to identify the (direct) molecular targets of this transcription factor in the ureteral urothelium. Although such assays would be interesting with respect to the dilatation of adult Pparg-cKO ureters, we deem them beyond the scope of the paper. However, we have added a section to the “discussion”, where we discuss the morphological change of the mutant ureter, and its possible causes (see above). <<

* Further discussion on GRHL3 as the alternative input for luminal differentiation program would be great. 1) Grhl3 is known to bind to the Upk2 promoter, but the discussion mentions Upk1b and Upk3b as being the Upks that are down-reglated but not absent in the Pparg-cKOs (not UPK2). 2) It would be interesting to see this discussion explore why Grhl3 is reduced in Pparg-deficient bladder urothelium (Liu et al 2019), but Grhl3 is not PPARg regulated in this model in ureteral urothelium, and GRHL3 was not explored.
>> In the original version of our manuscript, we showed that Grhl3 expression is not changed in Pparg-cKO ureter at E16.5 and E18.5. We now also show that GRHL3 protein is unchanged (new Figure S10B). We have expanded our discussion on GRHL3 as an additional input in luminal differentiation as suggested by the reviewer. It now reads: “The expression of some S cell specific genes, including *Sptssb*, *Upk1b*, *Upk3b a*nd *Fa2h,* was reduced but not absent in the *Pparg-cKO* ureter, suggesting alternative activating inputs in expression of these genes. A strong candidate is the transcription factor GRHL3. In mice with a systemic loss of *Grhl3*, the cells of the luminal layer of the bladder epithelium (the ureter was not analyzed) were small and did not express UPKs and KRT20. Transcriptional profiling revealed strongly reduced expression of *Upk2* and *Snx31* (a gene involved in endosomal recycling), whereas expression of other *Upks* and S cell markers was less affected(Yu et al., 2009). In *Pparg-cKO* ureters, the expression of *Upk2* and *Snx31* was only slightly affected at E16.5 and E18.5. Importantly, the expression of *Grhl3*/GRHL3 was normal in the *Pparg-cKO* urothelium at these stages. These results suggest that PPARG and GRHL3 are independently required for S cell differentiation in the developing ureter and bladder. Some S cell specific genes (*Upk2*, *Snx31)* may be preferentially activated by GRHL3, some by PPARG (*Krt20*, *Hsd17b2*), while (most?) others (e.g. *Upk3b*) may be co-regulated by both transcriptional activators. In mice with a conditional loss of *Pparg* in the bladder epithelium, *Grhl3* expression was reduced in an RNA-Seq analysis of adult mutant bladders (Liu et al., 2019). As *Grhl3* expression has not been validated at mRNA or protein level in adults, or at fetal stages where secondary changes can be excluded, it remains unclear whether PPARG acts upstream of *Grhl3* in the bladder urothelium.
<<

© 2025. Published by The Company of Biologists under the terms of the Creative Commons Attribution License (https://creativecommons.org/licenses/by/4.0/).

Reviewer 2:

SUMMARY OF THE ADVANCE MADE IN THIS PAPER AND ITS POTENTIAL SIGNIFICANCE TO THE FIELD

* Overall: This report adds mechanistic detail to the existing knowledge that PPARG is involved in urothelial differentiation; specifically it shows that PPARG acts via shh in basal and intermediate cell types but probably by different paths in superficial cells. The work is done well and the figures are of high quality. The question will be of interest to a rather narrow readership, and as the authors correctly point out at the top of page 4, quite a lot is already known about PPARG in urothelial differentiation and the main advance here is to put shh in the pathway. Again, as the authors point out on page 4, the main interest will be to the cancer community (urothelial carcimonas). Therefore, while I think this paper is worth publishing (after minor corrections), I am not convinced that Development is the best place for it. This is not a comment on quality (I refuse to play those games - to me a paper is either worth publishing or is not, and 'rankings' of journals are irrelevant to my recommendations), but about core interests of the journal's readership. I would have thought the urology or cancer journal communities would be more interested and result in more citations and conference invitations and collaborations for the authors. But if the authors really want to reach a developmental community with this very specialized topic, fine.

>> We thank the reviewer for taking the time to evaluate our manuscript. We are happy that the reviewer appreciates the quality and value of our work.
It is always a critical question which journal to choose for submission. Considering the possible impact of our work for the cancer field, a cancer journal would have been a possibility. However, we think that our work deals with basic molecular mechanisms of urothelial development, which is more relevant to the “developmental biology” community. Moreover, we think that we are not experts enough to establish cancer models, which reviewers in a cancer journal would have probably asked for.
<<

SUGGESTIONS TO AUTHORS

* I liked the paper but I do recommend some minor changes: Abstract: please define PPARG at its first use (ie give the full name).

>> We now define Pparg in the abstract.
“Here, we show that mice with a conditional deletion of the transcription factor gene peroxisome proliferator activated receptor gamma *(Pparg*) in the ureteric epithelium …” <<

*Abstract: '2-layered urothelium' is ambiguous (can be read as meaning the whole thing is doubled): can you say 'urothelium with only two cell layers, not the usual....'?

>> We have paraphrased this sentence. It now reads: “… …. have a dilated ureter at postnatal stages with a urothelium consisting of a layer of undifferentiated luminal cells and a layer of proliferating basal cells” <<

* Abstract: the sentence starting 'Molecular analyses' would be clearer if it were re-ordered to say 'Molecular analyses of fetal stages showed....'

>> We reordered the sentence as suggested by the reviewer. <<

* Abstract: in the sentence about pharmacological manipulation, make clear that the reduction of ureter width is towards the normal width (not from normal to too-small).

>> To accommodate the concern of the reviewer, we now write: “Pharmacological activation of SHH signaling in explant cultures of *Pparg*-deficient perinatal ureters reduced ureteral width and urothelial cell number to normal”. <<

* (apologies if the above comments seem pedantic, but especially in the era of AI- assisted searches, it is really important that abstracts are not written in a way that can be read with the opposite of the intended meaning).

>> We very much appreciate the corrections of the reviewer. As non-native speakers we are not always aware of the subtleties of the English language. <<

* General: please standardize on whether you have spaces after the closing parenthesis in

© 2025. Published by The Company of Biologists under the terms of the Creative Commons Attribution License (https://creativecommons.org/licenses/by/4.0/).

phrases like (S-)Cells {you sometimes have (S-) Cells and sometimes (S-)Cells}.
>> We have changed these abbreviations throughout the text and now coherently write: superficial (S) cells, intermediate (I) cells, basal (B) cells. <<

* Intro paragraph 2: 'choreographed' seems a strange word (who is the choreographer meant to be?). Do you just mean 'reproducible' or 'standard' or 'stereotypic'? Also in this paragraph, I advise making clear that in the ureteric bud, 'distal' means furthest from the kidney: in lung etc., the convention is opposite, and this regularly causes confusion in readers who are not renal/urology specialists.
>> We have rephrased the sentence to avoid possible confusion. It now reads: "This urothelial cytoarchitecture derives from highly coordinated proliferation, patterning and differentiation processes that act on epithelial progenitors (the cloaca in case of the bladder, the stalk region of the ureteric bud in case of the ureter) starting in the mouse around embryonic day (E) 10.5." <<

* Last paragraph page 3 - 'paramount' seems a very odd word but I cannot work out what word you really want; maybe just 'important'.
>> Thanks for this advice. We have replaced "paramount" by "important". <<

* Results paragraph 1 - in Fig 1, as far as I can see, you did not look at transverse sections of embryos (as you write) but transverse sections of ureters. You need to add 'ureters of' ahead of 'mice'.
>> As suggested by the reviewer, we have rephrased this sentence to:
"…we performed RNA *in situ* hybridization analysis on transverse ureter sections of wild-type mice at different prenatal and postnatal stages." <<

* Legend to Fig 1 - why is mRNA preservation harder in the adult? Please could you explain this (in the results text accompanying Fig 1 rather than the legend, I suggest). I wonder if a quick qRTPCR is warranted just to check that the apparent fall of mRNA but not of protein really is an artefact of preservation, and not a real change in the relative stabilities of the mRNA and of the protein in adult life. If the latter is true, it would be a shame to have missed it in this paper! (I am not 'insisting' on this change as a reviewer, just recommending).
>> We apologize for the technical insufficiency to perform RNA in situ hybridization analysis of Pparg expression on sections of adult ureters. In fact, postnatal ureters get increasingly tenacious making tissue fixation and preservation of the cellular mRNA difficult. However, we worked hard and optimized the fixation of the tissue, managing to preserve mRNA to perform RNA in situ hybridization for *Pparg*. Extending our previous analysis of postnatal stage-ureters (as recommended by reviewer 1), we now show that *Pparg* mRNA is expressed throughout the urothelium at all postnatal stages analyzed. <<

* Results end of page 5 - I recommend you explain just a bit more about hydroureter for readers who are not urologists (at least, if you go ahead with this being in Development rather than a urological journal).
>> Since reviewer 1 asked for a quantification of the ureteral lumen in *Pparg-cKO* ureters at P40, and a clarification of the shape with respect to hydroureter, we have performed a quantification of the luminal circumference (new Fig. S3A), and have rephrased this section: "However, histological analysis of *Pparg-cKO* ureters at P40 revealed a greatly enlarged tubular lumen with an irregular outline (Fig. 2A, Fig. S3, Table S2)."
We have decided to delete the reference to hydroureter in the result section. Instead, we have added a new section in the discussion on the ureteral dilatation in *Pparg- cKO* mice and their possible causes. <<

* I like Fig 2D! - very useful.
>> Thanks a lot! <<

* Text describing Fig 4B (in the results section) - you use phrases like 'increased proliferation' in a study that involves a time-course. Please make clear that you mean increased in comparison with control, not increased in comparison with earlier times, which is a perfectly natural way of reading the word 'increased'. (assuming you do mean in comparison with controls - if you really do mean with respect to time as well, explain that carefully). This

© 2025. Published by The Company of Biologists under the terms of the Creative Commons Attribution License (https://creativecommons.org/licenses/by/4.0/).

comment applies to several sentences in the paragraph.
>> The pressure to stay in the word limit, made us shorten sentences. We apologize if this caused unclear relations between the genotypes and the phenotypic changes. We have rephrased the paragraph on the proliferation, as well as other phenotypic descriptions to heal this. We hope that things become clearer. In this section, we now write:
„Proliferation was detected by the Ki67 antigen. In the control ureter, urothelial proliferation reached approximately 10% at E18.5, and decreased to 1% at P7. In the *Pparg-cKO* urothelium, proliferation was 15% at E18.5 and 8% at P7. The increased proliferation in the mutant compared to the control urothelium at E18.5 was caused by an increased proliferation in the luminal (KRT5$^-$) cells. At P7, luminal cells were quiescent in both mutant and control urothelium, but proliferation of (KRT5$^+$) cells in the basal layer was greqatly increased to 12% in the mutant urothelium compared to 1% in the control urothelium (Fig. 4B-D, Table S5). <<

To unbiasedly identify' need to be "To identify, in an unbiased manner, ".
>> We have changed the sentence as suggested by the reviewer to: "To identify, in an unbiased manner, the ". <<

* Also, in this paragraph, please justify the thresholds you used for microarray analysis. What happens if you use different ones? (Convince the reader you did not just fiddle about with thresholds until you saw something that you liked).
>> The thresholds are frequently used by us and others to sort out background noise and to detect robust changes in microarray data sets.
To better explain our threshold values we now write:
"Using an intensity threshold of 100 to reduce expression noise and fold changes of at least 1.5 to detect robust expression changes in the two individual arrays" <<

* Final sentence of the paragraph on page 8 (before the next heading). Be careful about the claim - you don't know that PPARG controls the expression of these genes directly. All you know is that its presence/ absence affects whether or not they are expressed.
>> To acknowledge the concern of the reviewer, we have rephrased the sentence to: "We conclude that *Pparg* is required for the expression of a unique set of genes in the developing urothelium of the ureter, namely *Shh* and a few other genes in the cells of the basal layer and a large number of S-cell specific genes in the luminal cells". <<
Discussion: I strongly advise deleting 'master' from the subheading; it binds you to a particular view of biology that is unnecessary and unhelpful, and gives critics a target at which to shoot. Just 'regulator' says all you need to say.
>>We have deleted "master" from the subheading in the revised discussion.

Reviewer 3:

* The manuscript by Rudat et al. thoroughly examines the consequences of Pparg deletion in the mouse ureter. They show that Pparg regulates the formation/maintenance of the ureter in a Shh-dependent and a Shh-independent fashion. The experiments are well executed and clearly support the authors conclusions. There are just a few critiques that should be addressed before I can support publication in Development.
>> We would like to thank the reviewer for taking the time to evaluate our manuscript. We are happy about his/her appreciation for the quality and value of our work. <<

* 1. The authors should provide images for the KI67 staining (in particular in co- staining with Krt5) in the main figure. If they need space, they can move the images of the apoptosis to the Supplemental Material, as these don't show an effect.
>> New Figure 4 now includes both TUNEL and Ki67/KRT5 images. <<

* 2. The basal Krt15 expression in Fig. 6B is not really visible. Thus, it really does not support the claim of the authors. More convincing images need to be provided.
>> We disagree with the reviewer that the KRT15 staining is not visible. It is a weak expression, but as we think, clearly visible.
To make it easier for the reader and reviewer, we have marked the KRT15+ cells by arrowheads in the revised version of Figure 6. <<

© 2025. Published by The Company of Biologists under the terms of the Creative Commons Attribution License (https://creativecommons.org/licenses/by/4.0/).

* Similarly, it is unclear, why the authors in their statistical analysis of the Krt15 data (Supplement al Table S12) used inconsistent statistical parameters (nonparametric vs. parametric normality, equal vs. unequal variance) and two-sided Mann-Whitney instead of the two-sided unpaired t-test used for all the other comparisons. Please expand on this.
>> We did not use inconsistent statistical parameters, but followed a scheme, which we have outlined in the methods section.
We always compared two groups for statistical analysis to choose the optimal test for the present datasets.
We followed this consistent scheme:
To decide whether or not the single groups follow a normal distribution of the measured data, we performed the Shapiro-Wilk test.
(Especially, counting 0% KRT15+ cells in the wildtype situation results in a non-parametric distribution of this parameter. This is why it is not recommended to compare this data to others by using a two-sided unpaired t-test. Instead, we employed the Mann-Whitney test, which uses a rank based strategy and therefore is not in need of a parametric distribution.)
If the Shapiro-Wilk test rejected the parametric distribution, we used the non-parametric Mann-Whitney test.
With both datasets being parametric, we further tested for variance using the F-test.
If they followed a F-distribution, a t-test was possible. If they did not, a t-test with Welch's correction was performed.
Thus, we now write in the methods (statistics) section:
„To compare two groups with normal distribution and equal variances the unpaired two-sided t-test was applied. The two-sided Welch's t-test was used if two groups with normal distribution had unequal variances. The two-sided Mann-Whitney U-test was used to compare two groups with non-parametric distribution.“ <<

* 3. The microarray analysis of the explanted ureters detected decreased expression of Keratins such as Krt6b, Krt16, Krt17, yet no validation of these data has been shown. The authors need to do so, to further support their data interpretation. The same should be done for some of the partially 'rescued' S-cell markers.
>> As suggested by the reviewer we have performed RT-qPCR analysis to confirm the data from the transcriptional profiling of the rescue experiment. We have added them as new a new panel in Figure 7 (Fig. 7G-I). RT-qPCR analysis confirmed the microarray data for *Hhip, Krt14, Krt15* and all S cell genes. For *Krt6, Krt16* and *Krt17*, we found inconsistent results, possibly due to low levels of expression.
For this reason, we toned down the statements on tissue stiffening.

* 4. The authors published a couple of years ago the deletion of the Shh mediator Smoothened (Bohnenpoll et al., 2017). While the authors cite the paper, it is surprising that the authors never explicitly compare the results of the present study (which highlights the role of Pparg in regulating Shh expression), with the outcome of this study. Similarly, Bohnenpoll et al. (2017) identified Foxf1 as a major regulator for ureter development, but its expression is not examined in the present manuscript. This should be rectified.
>> In fact, we published a couple of years ago a paper, where we analyzed the phenotypic consequences of the loss of Smo in the early undifferentiated ureteric mesenchyme. We showed that SHH signaling in the early undifferentiated UM regulates via Foxf1 and Bmp4 in the UM the coordinated proliferation and differentiation of the epithelial and mesenchymal primordia. We also showed that Foxf1 is required in the undifferentiated ureteric mesenchyme for epithelial and mesenchymal differentiation.
The current analysis deals with the later role of SHH after epithelial and mesenchymal differentiation have occurred. This is a completely independent scenario. In fact, we published a couple of days ago that SHH signaling is required at early postnatal stages for lamina propria development - but not for SMC development. Foxf1 expression reoccurs at very low levels in the LP starting from P0 but its role is unclear.
Due to the strict word limit, the completely different function of the SHH-FOXF1-BMP4 axis in early ureter development, and the fact that SMC development is not primarily affected by loss of *Pparg,* we did not discuss the earlier work. <<

5. The authors should also discuss in more detail why Inhibition of SHH signaling by

© 2025. Published by The Company of Biologists under the terms of the Creative Commons Attribution License (https://creativecommons.org/licenses/by/4.0/).

cyclopamine in ureter explant cultures does not affect urothelial development similarly as in the Pparg-deficient mice, while hyperactivation actually rescues a much substantial aspect of the phenotype.

>> Due to the different comments of the reviewers we have largely rewritten the discussion. In fact, we believe that PPARG-mediated SHH expression in the postnatal ureter is not required for urothelial maintenance, but is relevant for urothelial regeneration once functional S cells are lost. Please read our expanded section on this point. <<

Second decision letter

MS ID#: dev.204324R1

MS TITLE: PPARG contributes to urothelial integrity in the murine ureter by activating the expression of Shh and of superficial-cell specific genes

AUTHORS: Carsten Rudat, Philipp Straube, Jan Hegermann, Mark-Oliver Trowe, Hauke Thiesler, Herbert Hildebrand, Lisa Witt and Andreas Kispert

Dear Dr Kispert,
I am happy to tell you that your manuscript has been accepted for publication in Development, pending our standard publication integrity checks.

Reviewer 1:

SUGGESTIONS TO AUTHORS

author revisions and defense are acceptable

Reviewer 2: The authors have considered all of my suggestions and have responded to them in an appropriate manner. I have no more concerns.

Reviewer 3

Advance summary and potential significance to field

The authors have appropriately addressed the comments raised in the initial review. While a more extended discussion of some of the points raised/responded would be helpful, the strict word limit unfortunately does not allow this. I now support publication.

© 2025. Published by The Company of Biologists under the terms of the Creative Commons Attribution License (https://creativecommons.org/licenses/by/4.0/).
